# Modeling Kinetics and Thermodynamics of Guest Encapsulation into the [M_4_L_6_]^12–^ Supramolecular Organometallic Cage

**DOI:** 10.1021/acs.jcim.1c00348

**Published:** 2021-09-10

**Authors:** Gantulga Norjmaa, Pietro Vidossich, Jean-Didier Maréchal, Gregori Ujaque

**Affiliations:** † Departament de Química and Centro de Innovación en Química Avanzada (ORFEO-CINQA), Universitat Autònoma de Barcelona, Cerdanyola del Valles, 08193 Cerdanyola del Vallès, Catalonia, Spain; ‡ Laboratory of Molecular Modeling and Drug Discovery, Istituto Italiano di Tecnologia, Via Morego 30, 16163 Genova, Italy

## Abstract

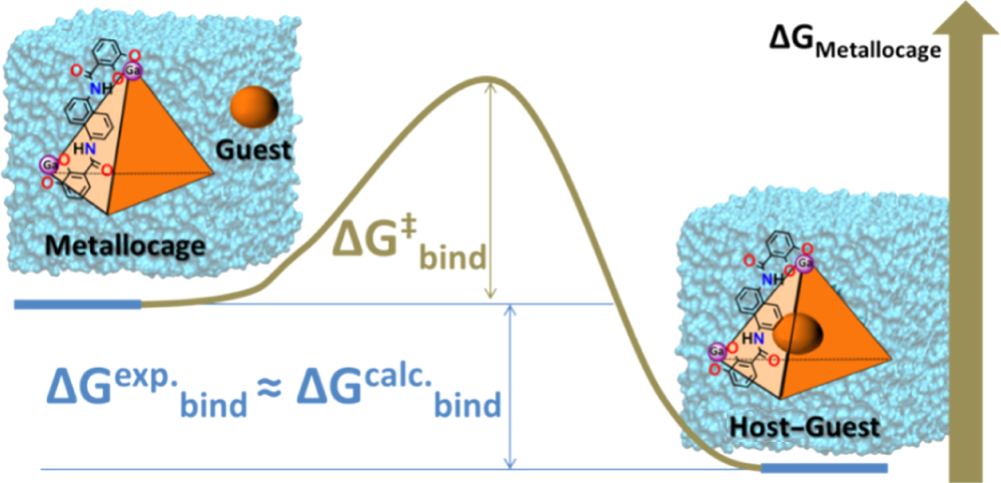

The encapsulation of molecular guests into supramolecular hosts is a complex molecular recognition process in which the guest displaces the solvent from the host cavity, while the host deforms to let the guest in. An atomistic description of the association would provide valuable insights on the physicochemical properties that guide it. This understanding may be used to design novel host assemblies with improved properties (i.e., affinities) toward a given class of guests. Molecular simulations may be conveniently used to model the association processes. It is thus of interest to establish efficient protocols to trace the encapsulation process and to predict the associated magnitudes Δ*G*_bind_ and Δ*G*_bind_^⧧^. Here, we report the calculation of the Gibbs energy barrier and Gibbs binding energy by means of explicit solvent molecular simulations for the [Ga_4_L_6_]^12–^ metallocage encapsulating a series of cationic molecules. The Δ*G*_bind_^⧧^ for encapsulation was estimated by means of umbrella sampling simulations. The steps involved were identified, including ion-pair formation and naphthalene rotation (from L ligands of the metallocage) during the guest’s entrance. The Δ*G*_bind_ values were computed using the attach–pull–release method. The results reveal the sensitivity of the estimates on the force field parameters, in particular on atomic charges, showing that higher accuracy is obtained when charges are derived from implicit solvent quantum chemical calculations. Correlation analysis identified some indicators for the binding affinity trends. All computed magnitudes are in very good agreement with experimental observations. This work provides, on one side, a benchmarked way to computationally model a highly charged metallocage encapsulation process. This includes a nonstandard parameterization and charge derivation procedure. On the other hand, it gives specific mechanistic information on the binding processes of [Ga_4_L_6_]^12–^ at the molecular level where key motions are depicted. Taken together, the study provides an interesting option for the future design of metal–organic cages.

## 1 Introduction

Host–guest interactions play an essential role in supramolecular chemistry, a rapidly growing field of research.^1^ Among the hosts developed, supramolecular organometallic cages (SOCs) play a prominent role.^2,3^ SOCs, also named metal–organic cages (MOCs) or metallocages, are supermolecules generated by self-assembly of metal ions or clusters with organic ligands.^4−8^ They have been extensively studied because of their interesting structures that account with a variety of topologies and well-defined shapes at the nanoscale. The capability for tailoring the cavities of the SOCs has led to develop a rich host–guest chemistry with many applications such as recognition of specific molecular targets,^9^ the purification of product mixtures,^10^ gas sorption,^11,12^ biomedicine,^13^ and catalysis.^5^

The rules governing the specificity and strength of molecular binding events for these systems are complex. Among key questions is the exact contribution of the different physicochemical variables involved in the process of encapsulation. From the thermodynamic point of view, the process is governed by the Gibbs energy associated with the encapsulating process. However, a molecular description of the process considering the flexibility of the cage, size, and shape complementarities, in between others, is highly desired.

**Scheme 1:**
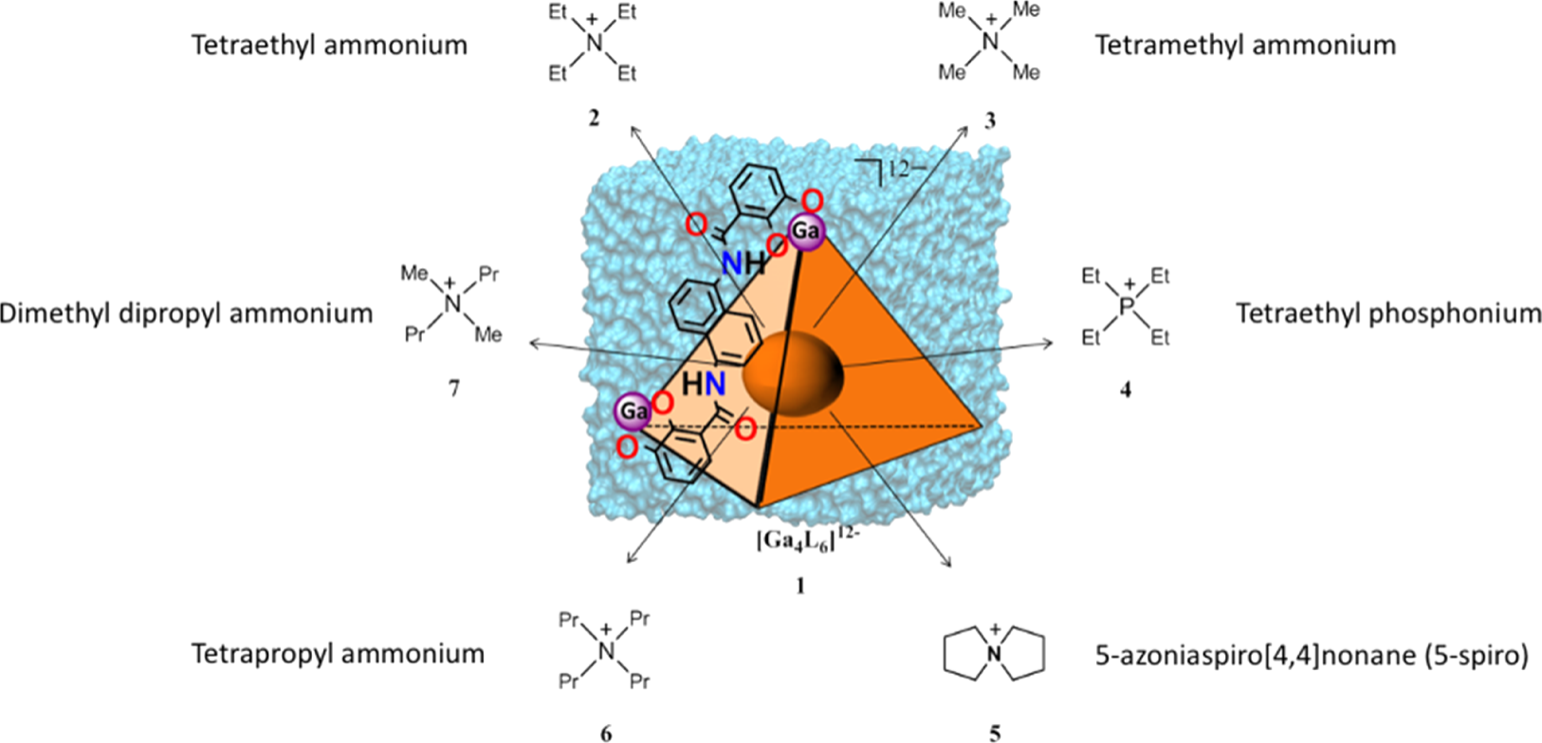
Chemical Structures of the [Ga_4_L_6_]^12–^ Metallocage (the Host) and Cationic Ligands (the Guests) Used in this Work

Among the families of SOCs that are receiving considerable attention, the self-assembled tetrahedral metallocage, [Ga_4_L_6_]^12–^ (**1**, Scheme 1), developed by Raymond and co-workers has shown versatility to encapsulate cationic guests and perform host–guest catalysis.^5^ Recent studies by our group and others have demonstrated that computational chemistry can have major implications on understanding the mechanism of metallocage-supported catalysis.^14−19^ So far, the works performed were based on quantum chemistry approaches combined with limited conformational sampling performed via molecular dynamics (MD) (either based on force fields or quantum approaches).^14,15,17−20^ These works were focused at decoding the particularities of the host–guest catalytic mechanism compared to the reaction taking place in solution. Among the most striking results was the identification that the few solvent molecules that access the host cavity are fundamental for the catalytic effect provided by the supramolecular assembly. In these studies, however, another fundamental aspect in host–guest chemistry—the encapsulation process—was not addressed.

**Scheme 2:**
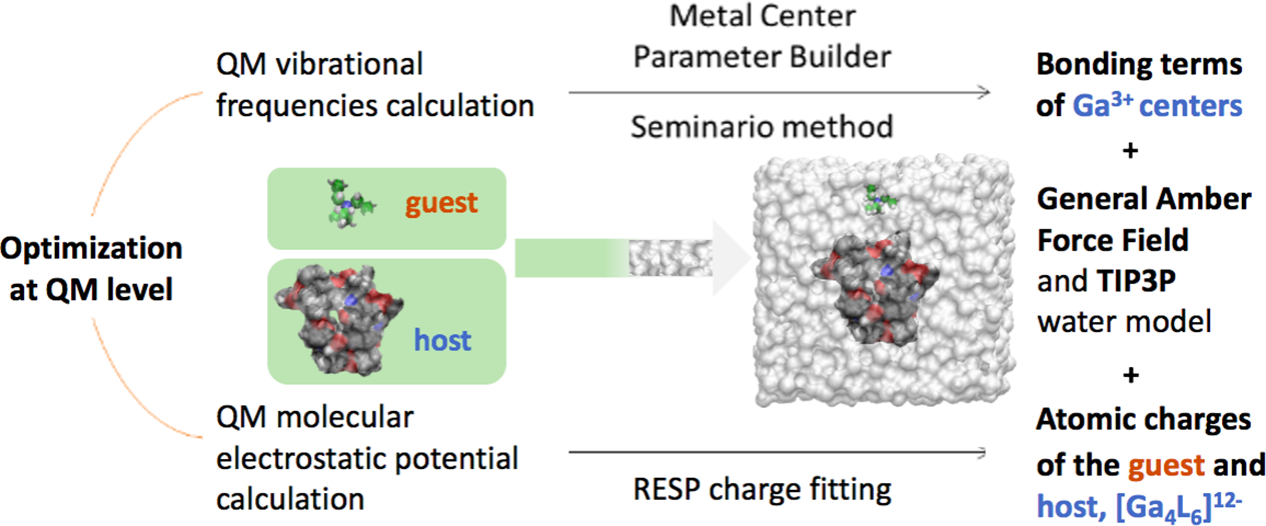
General Force Field Parameterization Procedure for Host and Guest Molecules Investigated in This Study

The experiments on guest-exchange dynamics showed that the [Ga_4_L_6_]^12–^ SOC deforms to enlarge an entrance for the passage of the guest, instead of transiently breaking a metal–organic ligand bond.^21,22^ Previous MM3 calculations in the gas phase in combination with experiments of the guest displacement from the cage have been reported, although these calculations are based on constrained searches on the potential energy surface that do not account for the full flexibility of the system.^23^ However, uncovering the dynamics and structural fluctuations of these supramolecular assemblies in solution is fundamental to properly describe the process and for the rational design of novel cages.^24^

When it comes to simulate binding processes by computational means, techniques allowing extensive conformational samplings are necessary, something unsustainable in a quantum mechanics-based framework whose computational cost is still demanding. In many fields, classical MD simulations, in combination with enhanced sampling techniques, proved successful for the calculation of binding Gibbs energies.^19,25−29^ In principle, classical MDs offers an excellent opportunity to study host–guest binding processes at the atomic scale and evaluate their thermodynamics profiles. However, the application of classical MD to SOCs is not free of challenges. One of the most important points is to establish an appropriate protocol in terms of atomic parameterization and conformational sampling. Derivation of metal-compatible force fields is still one of the most complicated steps in parameterization of atomic force fields.^30−33^ Regarding the encapsulation process, there are only very recent works addressing this issue.^34,35^

Here, we report a study aimed at both, establishing a computational framework valid to calculate both Gibbs binding energies (Δ*G*_binding_) and Gibbs energy barriers (Δ*G*_binding_^⧧^) associated with the host–guest encapsulation process, as well as providing an atomistic description for the encapsulation process itself. To do so, we take advantage of the experimental data available on the [Ga_4_L_6_]^12–^ metallocage (**1**) (Scheme 1), which we used as a benchmarking system. We computed the binding Gibbs energies of several cationic guests into **1** and described the encapsulation process at the molecular level in aqueous solution using MD simulations and subsequent statistical analysis. Furthermore, the potential of mean force (PMF) for the encapsulating process was also investigated. The atomistic description of this process revealed very interesting motions in the metallocage and rearrangements in the system.

## 2 Computational Details

### 2.1 Derivation of Force Field Parameters

The supramolecular metallocage, **1**, contains a Ga^3+^ metal center on each vertex of the tetrahedron. A bonded scheme was employed to describe these centers, according to which the interactions between the metal and its ligands were modeled using harmonic potentials for bonds and angles (the AMBER force field functional form), while rotations around metal–ligand bonds were not explicitly included (as is customarily done for organometallic systems). The parameters were derived using the Python-based metal center parameter builder (MCPB)^36^ developed by Merz and co-workers and based on Seminario’s method.^37^ The parameters of the bonding terms for the organic moieties of the metallocage and for the guests were taken from the general AMBER force field (gaff).^38,39^ Van der Waals parameters were obtained from the optimized potentials for liquid simulations force field (OPLS),^40,41^ except those of the metal (Ga^3+^) taken from the universal force field (UFF).^42^ Atomic charges were computed by fitting the molecular electrostatic potential computed at the quantum mechanics (QM) level (either in the gas phase or including an implicit solvent representation—see the Results and Discussion section) according to the restrained electrostatic potential (RESP) method.^43^ To this aim, we optimized the full metallocage and each ligand at the density functional theory (DFT) level, as described below. The antechamber^44^ program was used to assign atom types and to derive atomic charges (Scheme 2).

### 2.2 Quantum Chemical Calculations

DFT geometry optimizations were performed with the B3LYP-D3 functional^45−47^ (including three body correction terms) using the Gaussian 09 program.^48^ The SDD pseudopotential^49^ and related basis set complementing with a set of d polarization functions^50^ were used for gallium, while the 6-31G(d) basis set^51^ was used for all other atoms. Optimizations were performed separately for the metallocage and each ligand, both in the gas phase and using the SMD solvation model^50^ to account for solvation effects. Binding energy estimates at the QM level account for thermal effects via the quasi-rigid-rotor-harmonic-oscillator (quasi-RRHO) approach.^52^ The standard state correction^53^ (1.9 kcal/mol to each of the compounds) was also included.

The cavity volumes for the metallocage were obtained using the CAVER Analyst 1.0 software tool^54^ employing the default probe radii. The molecular surface areas of the metallocage were calculated using UCSF Chimera.^55^

### 2.3 Molecular Simulations

MD simulations were performed using the CUDA version of the pmemd program from the AMBER 16 package.^56^ The MD simulation box, of size 46 × 49 × 47 Å and treated under periodic boundary conditions, contains metallocage **1**, the guest, 11 potassium counterions, and ∼2500 water molecules (TIP3P).^57^ The simulations were performed at constant temperature (298.15 K, using a Langevin thermostat) and pressure (1 bar, using a Monte Carlo barostat^58^). A cutoff of 9 Å was used for nonbonded interactions, with long-range electrostatics interactions accounted by the PME method.^59^ A time step of 2 fs was used in plain MD simulations, whereas for Gibbs energy calculations, different time steps were used according to recommendations from the literature.

The absolute Gibbs energies of binding (Δ*G*_bind_) of each ligand were computed using the attach–pull–release (APR) method.^60^ This method allows for precise calculations of supramolecular host–guest binding, and may thus also be used to evaluate and optimize force fields.^60^ Three dummy atoms were used to setup the restraints required by the APR method, together with two atoms of the guest and three of the host (see Figure S12). The distance force constant was set to 5.0 kcal/(mol Å^2^), whereas the angle and torsional force constants were set to 100.0 kcal/(mol rad^2^). The distance between one atom of the guest and one dummy atom placed at the bottom of the metallocage was increased by 0.4 Å from 6 Å (guest inside the cage) to 24 Å (guest outside) during the simulation. For each simulated window, equilibration and accumulation included 500,000 minimization cycles, 1 ps NVT at 10 K, 100 ps heating to 298.15 K, 250 ps NPT equilibration, and 2.5–25 ns NPT production (depending on the standard error of the mean of the restraint forces). Hydrogen mass repartitioning allowed for a time step of 4 fs. Snapshots from APR simulation are shown in Figure S7.

The relative Gibbs energies of binding (ΔΔ*G*_bind_) were computed using alchemical transformations^61^ of guest **2** into guests **3**, **4**, **6**, and **7** (ligand **5** was not considered here because of the spiro substituents). The transformations were performed for the bound and unbound states using 12 values of the alchemical coupling parameter (0.00922, 0.04794, 0.11505, 0.20634, 0.31608, 0.43738, 0.56262, 0.68392, 0.79366, 0.88495, 0.95206, and 0.99078). The windows were sampled sequentially (starting from λ = 0.00922), performing 10 ns NPT simulations each. Since SHAKE was not applied during the alchemical transformation, a time step of 1 fs was used. ΔΔ*G*_bind_ was estimated by thermodynamic integration. Averages of ∂*H*/∂λ for each window were computed on uncorrelated data after removing the initial nonequilibrated part of the time series.^62^

The PMF for the binding of guest **2** was computed via umbrella sampling.^63^ The distance between the center of mass (COM) of the host (defined by the four Ga atoms) and the COM of the guest was selected as the reaction coordinate. A total of 38 windows (spaced by 0.4 Å) were used to cover the range between 0.7 and 15.1 Å (one further window was added in the transition-state region). A harmonic potential with a force constant of 5.0 kcal/mol was applied to sample configurations in each window. The production time was 2.5 ns for each window, and before the production run, every window was minimized and equilibrated as described above for the APR method.^60^ A time step of 4 fs was used. The weighted histogram analysis (WHAM) method^64^ was used to reconstruct the Gibbs energy profile.

## 3 Results and Discussion

The supramolecular tetrahedral metallocage **1** (Scheme 1) offers a particularly interesting system for benchmarking computational protocols for the encapsulation of ligands into metallocages. Indeed, ^1^H NMR spectroscopy experiments led to valuable observations in binding trends. The values for binding Gibbs energies show that metallocage **1** preferentially encapsulates NEt_4_^+^, **2** (−6.2 kcal/mol^9^ and/or −6.3 kcal/mol),^65^ over its smaller NMe_4_^+^, **3** (−3.4 kcal/mol)^10^ and larger NPr_4_^+^, **6** (−2.8 kcal/mol^9^ and/or −4.3 kcal/mol)^65^ counterparts.

The first step in the computational study of guest encapsulation in **1** was centered on NEt_4_^+^ and consisted in assessing an optimal parameterization procedure for the different molecular entities compatible with the AMBER force field. Once the benchmark on this system was performed, the same protocol was applied for the calculation of binding Gibbs energies of other cationic guests, which we compared to experimental data. Next, the Gibbs energy profile for the whole encapsulation process was investigated.

### 3.1 Parameterization for Binding Gibbs Energy Calculations: NEt_4_^+^ ⊂ [Ga_4_L_6_]^12–^

**Figure 1:**
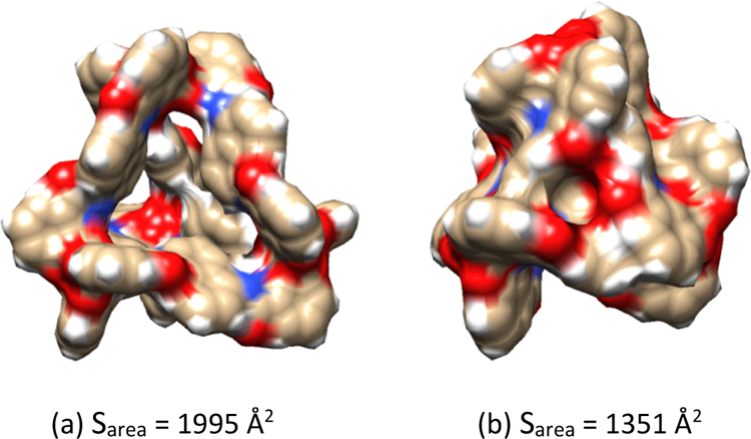
DFT-optimized geometries of the metallocage, **1**, (a) in the gas phase and (b) in the implicit water solvent.

The metallocage under study is highly charged, [Ga_4_L_6_]^12–^. One of the first questions in terms of modeling is to assess how the presence of the solvent could affect the physicochemical properties of the metallocage and how this could influence the step of force field parameterization. We started by optimizing the geometry of the metallocage at the QM level. We observed significant differences between vacuum and continuum aqueous solvent conditions in terms of size and shape (Figure 1). For instance, the surface area of the metallocage is 1995 Å^2^ in the gas phase, whereas it is 1351 Å^2^ in the implicit water solvent. Thus, accounting for solvent effects provokes a shrinking of the metallocage, accompanied by a decrease of the host cavity.

This observation prompted the question on the influence of the medium on the derived parameters for the Ga^3+^ centers. To answer it, we systematically derived the force field parameters from both the gas phase and implicit solvent calculations and checked the effect of the host and the guest parameterizations on the computed Gibbs binding energy (Table 1). As an informative additional calculation, we also provide the binding Gibbs energy obtained with the AMBER-BCC charges for the guest, whose calculated binding Gibbs energy is −8.2 ± 0.6 kcal/mol (entry 9 of Table 1). The binding Gibbs energies are calculated to be between −2.1 and −11.2 kcal/mol depending on the atomic charges of host and guest when the bonding terms for the Ga^3+^ centers are derived in the gas phase. These values are in a narrower range of −2.5 to −6.3 kcal/mol when the bonding terms for the Ga^3+^ centers are derived in the implicit aqueous solvent.

**Table 1:** Binding Gibbs Energies for **2** ⊂ **1** Computed with the APR Approach Using the Force Field Parameters Derived from the Gas Phase or Continuum Water Solvent Calculations

	bonding terms of Ga^3+^ centers derived in	host charges calculated in	guest charges calculated in	Δ*G*_calc._°	Δ*G*_exp._°
entry 1	vacuum	solvent	solvent	–9.8 ± 0.8	
entry 2		solvent	gas phase	–11.2 ± 0.8	
entry 3		gas phase	gas phase	–2.1 ± 0.5	
entry 4		gas phase	solvent	–4.0 ± 0.6	
entry 5	**solvent**	**solvent**	**solvent**	**–6.3±0.6**	**–6.2**
entry 6		solvent	gas phase	–5.7 ± 0.6	
entry 7		gas phase	gas phase	–2.5 ± 0.6	
entry 8		gas phase	solvent	–2.5 ± 0.5	
entry 9		solvent	AMBER-BCC	–8.2 ± 0.6	

The experimental Gibbs energy of binding of NEt_4_^+^**2** into **1** is −6.21 kcal/mol, as estimated from the equilibrium constants measured at 25 °C in water. Table 1 shows that the calculation of both the charges and bonding parameters from continuum solvent calculations is the one with the better agreement with the experimental value (−6.3 ± 0.6 kcal/mol; entry 5). All other protocols lead to values that differ between 2 and 5 kcal/mol from the experiment (except for entry 6 with less than 0.5 kcal/mol difference). These results confirm the importance of accounting for solvent effects during the parameterization.

The values shown in Table 1 illustrate that for Ga^3+^ bonding parameters derived in vacuum, the effect of host charges derived in the gas phase and solvent in the binding Gibbs energy (ΔΔ*G*_calc._°) is 5.8 kcal/mol for guest charges derived in the solvent (entry 4–entry 1), whereas this effect accounts for 9.1 kcal/mol when guest charges are derived in the gas phase (entry 3–entry 2), respectively. The effect of guest charges, in turn, is 1.4 kcal/mol for host charges derived in the solvent (entry 1–entry 2) and 1.9 kcal/mol for host charges derived in the gas phase (entry 3–entry 4). The effect of host charges on the Δ*G*_calc._° is clearly higher than the effect of guest charges.

The analogous analysis taking Ga^3+^ bonding parameters derived in the solvent shows that the effect of host charges derived in the gas phase and solvent (ΔΔ*G*_calc._°) is 3.8 kcal/mol for guest charges derived in the solvent (entry 8–entry 5), whereas this effect is 3.2 kcal/mol when guest charges are derived in the gas phase (entry 7–entry 6). The effect of guest charges, in turn, is null for host charges derived in the gas phase (entry 8–entry 7) and 0.6 kcal/mol for host charges derived in the solvent (entry 8–entry 5). As in the previous case, the effect of host charges on the Δ*G*_calc._° is larger than the effect of guest charges, but the differences obtained are reduced.

As far as the bonding terms (Δ*G*_calc._°) are concerned, regarding bonding terms for Ga^3+^ calculated in vacuum or the solvent, those obtained in vacuum tend to give lower binding energies (more negative) than their counterparts derived in the solvent (except for the comparison of entry 3 with entry 7). When all parameters are derived in vacuum (entry 3 of Table 1), the computed Gibbs binding energy is −2.1 ± 0.5 kcal/mol, providing an estimate much worse than that obtained when all parameters are derived in the solvent (entry 5, −6.3 ± 0.6 kcal/mol). Indeed, the best agreement with experiment is obtained when all parameters are derived in the solvent. Thus, this is the selected parameterization protocol used in the following part of the study.

At this point, it is worth comparing the above results from simulations with the binding estimate based on quantum chemical calculations. The Δ*G*_binding_ obtained for this system with an implicit SMD solvent at the B3LYP-D3 level is −19.6 kcal/mol, overestimating the strength of binding and suggesting that accounting for an explicit solvent and flexibility are important ingredients for accurate modeling. The DFT-optimized structures are shown in Figure S1. It is worth mentioning that a mixed implicit–explicit solvent method was shown to work properly for complexation of self-assembled capsules in water, but a thorough study to determine the number of solvent molecules to be included is needed.^66^

Overall, based on the results of this part of the study, the APR protocol based on force field parameters (both bonding terms and atomic charges) derived from implicit solvent DFT calculations provides a very good agreement with experimental Gibbs binding energies.

### 3.2 Computing Binding Energies for a Set of Guests

#### 3.2.1 Robustness of the Method

**Figure 2:**
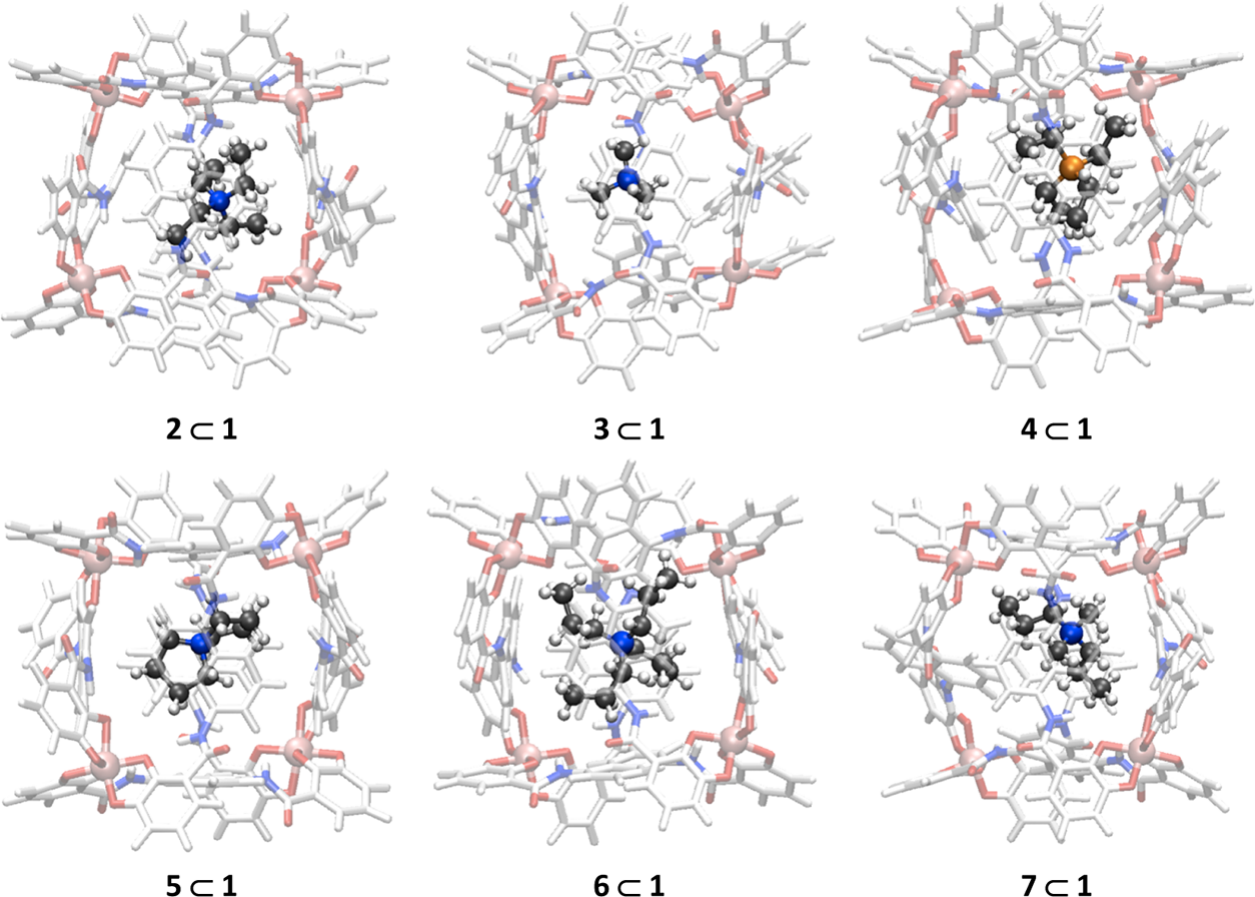
Representative structures of the host–guest complexes considered in this study.

To further assess the suitability of the parameterization procedure characterized in the previous part of the study for a general purpose in supramolecular chemistry, the binding Gibbs energies of five additional cationic guests (**3**, **4**, **5**, **6**, and **7**) with known experimental values were calculated (Figure 2). The same protocol based on the parameters derived according to the scheme described in the previous section was employed.

Calculated and experimental energies of binding are in very good agreement for all guests (Table 2). For complexes **4** ⊂ **1**, **5** ⊂ **1**, and **7** ⊂ **1**, differences are ca. 1.0 kcal/mol, while for **3** ⊂ **1** and **6** ⊂ **1**, they are ca. 2 kcal/mol. It is quite remarkable to reach such quality, indicating that the combination between force field and the sampling protocol is quite robust. Plotting the experimental against the computed values shows that there is good linear relationship between both quantities, although the experimental range is narrower, leading to a slope <1 (Figure S4).

**Table 2:** Binding Gibbs Energies of the Host–Guest Complexes Considered in This Study

host–guest complex	Δ*G*_calc._°	Δ*G*_exp._°^22^	ΔΔ*G*
2 ⊂ 1	–6.28 ± 0.56	–6.21 ± 0.01 (−6.29)^65^	0.07
3 ⊂ 1	–1.15 ± 0.82	–3.42 ± 0.001^65^	2.27
4 ⊂ 1	–8.32 ± 0.63	–6.85 ± 0.03	1.47
5 ⊂ 1	–4.36 ± 0.60	–5.59 ± 0.01^65^	1.23
6 ⊂ 1	–0.62 ± 0.73	–2.75 ± 0.03 (−4.25)^65^	2.13
7 ⊂ 1	–5.86 ± 0.97	–4.80 ± 0.03	1.06

The computation of relative free energies of binding using alchemical transformations is a convenient technique to compare the affinity of ligands for a given receptor.^67−70^ Converting guest **2** into each of the remaining guests, both in solution and encapsulated in the metallocage, we estimated ΔΔ*G*_**2** → **j**_ (**j** = **3**, **4**, **6**, and **7**; see Table S2). The ranking of guests based on the ΔΔ*G* values is in line with the experimental trend. For guests **3** and **6**, the estimates are consistent with the APR results (within statistical errors), whereas for compounds **4** and **7**, the difference in the computed ΔΔ*G* is significant. For guest **4**, the FEP estimate is closer to the experimental data, whereas for guest **7**, the APR estimate is closer to the experimental result. The relative potential energies of binding were calculated, but they fail to reproduce the experimental trend (see Table S2).

Importantly, calculated binding Gibbs energies with APR reflect the same trend between encapsulation of compounds than those found experimentally (Table 2). Thus, the largest binding energies correspond to those species bearing four Et substituents (compounds **2** and **4**), whereas the lowest binding energies correspond to species bearing four Me or Pr substituents (compounds **3** and **6**; the origin for the similar behavior, however, is different for both species, vide infra). The binding energies of compounds **5** and **7**, bearing two cyclic butyl substituents and either two Me and two Pr substituents, respectively, lie in between. Moreover, guests with linear alkyl chains, **2** and **4** (−6.3 and −8.3 kcal/mol) have a higher binding affinity than their cyclic analogue **5** (−4.4 kcal/mol), which is consistent with the experimental observations on relative binding affinities between *n*-alkanes (C5–C8) and their cyclic isomers.^71^ This agreement further validates the computational protocol and suggests that molecular simulations may provide access to the molecular features that control the binding of guests to the SOC host. This is extremely relevant because the energetic behaviors between the different host–guest partners are somehow counterintuitive. Indeed, one would expect some correlation between the size and shape of the substituent such as methyl < ethyl < propyl. The simulations offer therefore a unique opportunity to better understand the molecular grounds of the energetics of encapsulation.

#### 3.2.2 Molecular Grounds for Binding Energy Trends

Owning a computational protocol that could help in defining the relative affinities of different guests to the same metallocage is crucial in supramolecular chemistry and constitutes one of the objectives of this work. Computing reliable binding affinities can be a precious tool for designing supramolecular hosts. Based on previous studies on supramolecular systems,^14,15,18,32,33,65,72^ several variables could relate to such prediction: (1) the cavity volume of the host alone, (2) the volume of the guest, (3) the packing values, that is, the ratio between the volume of the guest in front of the volume of the cavity of the host, and (4) the possible involvement of solvent molecules. It is likely although that the real relationship lies as a combination of these different factors.

**Figure 3:**
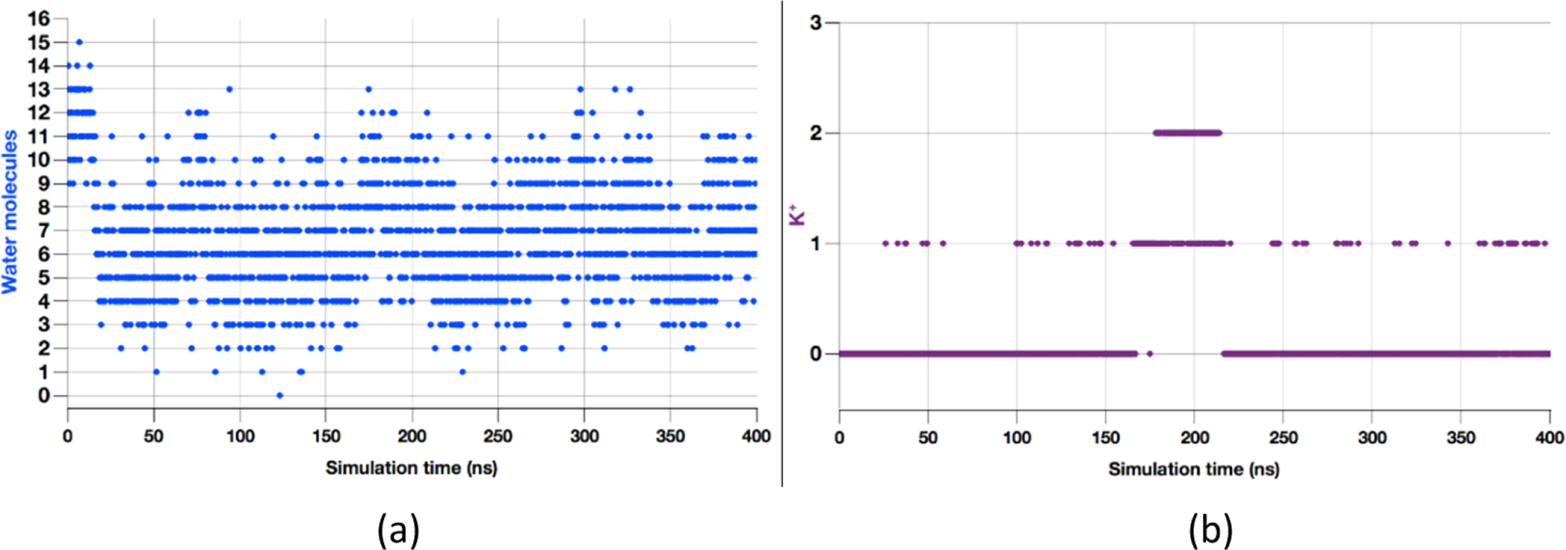
(a) Number of solvent water molecules in the metallocage during classical MD simulation. (b) Number of K^+^ located at a distance < 5 Å from the COM of the metallocage during classical MD simulation.

We started analyzing the metallocage **1** in solution without any guest. The volume for the free state of the cage ranges from void (meaning not even one solvent molecule could fit) to 480 Å^3^. The number of water molecules inside the cavity varies from 0 to 15 (Figure 3a), with an average of 6–9; this result is in agreement with that obtained by AIMD calculations of 9 ± 1 water molecules inside the metallocage.^73^ The number of K^+^ counterions in the close environment of the cage (up to 11 Å from the COM of the cage) mostly fluctuates between 3 and 6 (Figure S1), with up to two K^+^ inside the cavity (Figure 3b). For comparison, in the case of the encapsulation of a substrate, that is, **2** ⊂ **1**, the volume of the cavity fluctuates from ca. 180 to 530 Å^3^, with a mean of 318 ± 51 Å^3^ (Figure S3).

**Figure 4:**
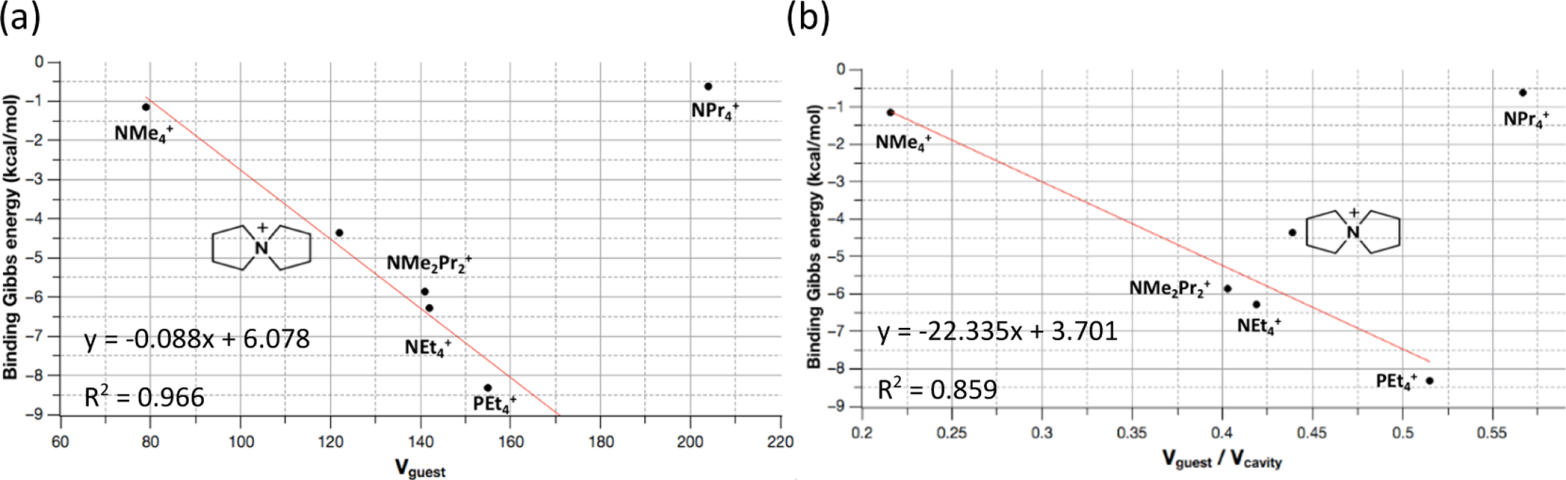
(a) Correlation between binding Gibbs energy and *V*_guest_. (b) Correlation between binding Gibbs energy and *V*_guest_/*V*_cavity_. In both cases, regression excluding compound **6**, NPr_4_^+^.

The large variability of the size of the cavity of the cage clearly indicates a high flexibility of the host. Importantly, these MD simulations suggest that the flexibility of the cage enables adaptation of its cavity to the guest to be encapsulated. Our purpose was to look for a correlation, if any, between some properties of the host and guest and the binding process. Interestingly, no correlation is observed between the binding Gibbs energy and the size of the cavity (the volume of the cavity is calculated for the most populated structure along the dynamics) for the six systems under study in this work (Figure S5). The same occurs when plotting the volume of the cavity and the volume of the guest, meaning that the structuring of the cage was not only a product of the size of the guest alone (Figure S6). The correlation between binding Gibbs energy and volume of the guest is surprisingly good, especially if guest **6** is not considered (*R*^2^ = 0.96 for theoretical values (Figure 4a) and *R*^2^ = 0.79 for experimental ones). The larger the *V*_guest_, the greater (more negative) the Gibbs binding energy. This is accomplished until the volume of the guest overcomes a certain threshold: all the guests fitting the correlation have a volume in the range 80–160 Å^3^, whereas **6** has a volume of ∼200 Å^3^. Further analysis of the **6** ⊂ **1** complex reveals that this guest tends to be partially outside the cavity (Figure S10), thus explaining the reason why the binding energy decreases dramatically despite having a larger volume. Overall, for those guests with a volume below the threshold, *V*_guest_ is an excellent predictive indicator for binding affinity trends. Those with larger volumes are expected to lie partially outside the cavity, consequently having lower binding energies.

The complexing properties were also evaluated as a function of the *V*_guest_/*V*_cavity_ ratio (Figure 4b). Close inspection of the values obtained shows that compound **6** acts also as a clear outlier. Its removal from the set allows an acceptable correlation (*R*^2^ = 0.86, Figure 4b). The best binding affinity is obtained for the encapsulation of **4**, whereas for compound **6**, displaying a similar *V*_guest_/*V*_cavity_ ratio, presents the worse binding affinity. The same reasoning for this guest explained before can be applied here: **6** tends to be partially outside the cavity of the metallocage. This explains why it is the worse binder among those considered for this metallocage. Overall, for those systems below the threshold, the *V*_guest_/*V*_cavity_ ratio, ranging from 0.22 to 0.51, it is also a good predictive indicator of binding affinity trends.

Next, we analyzed the packing coefficient of the guest in the internal cavity of the molecular cage. In order to be able to calculate packing coefficients, the presence of solvent molecules inside the cavity, if any, must be taken also into account. For that, we performed additional 200 ns MD simulation for each of the guests. The analysis of the structures along the MD could identify the average solvent molecules inside the cavity (Table S1): for **3**, there are four water molecules (*V*_guest_ ∼ 80 Å^3^), for **5**, there are three water molecules (*V*_guest_ ∼ 120 Å^3^), for **2**, **4**, and **7**, there are two water molecules (*V*_guest_ ∼ 150 Å^3^), and for **6**, there are no water molecules (*V*_guest_ ∼ 200 Å^3^). The *V*_cavity_, in turn, remains around 300 Å^3^ for all the guests, except for **6**, where the volume rises up to ∼450 Å^3^ (close to the highest volume of 480 Å^3^ found when encapsulating the largest amount of water, vide supra). A comparison among the guests indicates that the larger the *V*_guest_, the lower the number of water molecules inside the cavity. This allows keeping the packing values ranging from 0.55 to 0.63 (Table S1). These values are close to the theoretical value of 0.55 proposed by Rebek,^74^ constituting a rule of the thumb in supramolecular chemistry: the closer the packing to 55%, the better the binding affinity. For the case of **6**, the packing coefficient is 0.45, in line with this guest having the least binding energy.

The results obtained up to here clearly indicate that the APR method provides with very relevant results for the prediction of Δ*G*_binding_. Moreover, among the parameters computed, both *V*_guest_ and *V*_guest_/*V*_cavity_ produce good correlations with binding energies (for guests with a volume lower than a threshold). Thus, these two parameters are potentially useful for virtual screening of the ability of this metallocage to bind a guest. To satisfy Rebek’s rule on the packing coefficient, the solvent molecules present inside the cavity need to be also considered, which can conveniently be obtained by performing explicit solvent MD simulations. Overall, pure volumetric analysis of the guests could be already indicative of the binding affinity with the metallocage. This also highlights that computational approaches, such as the one we tested here, could be a good ally for both metallocage and guest selection and useful for supramolecular design.

### 3.3 Encapsulation of NEt_4_^+^ in the Metallocage: Molecular Insights on Encapsulation Profiles

**Figure 5:**
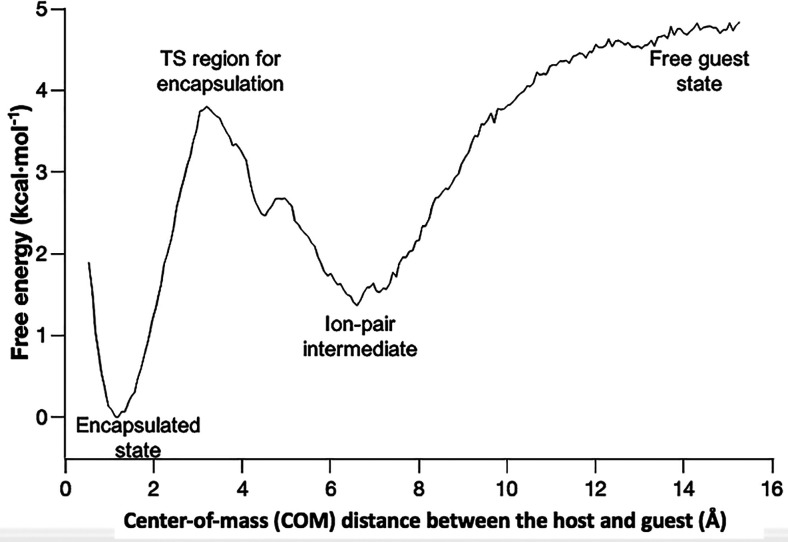
Free energy profile (PMF) of encapsulation process of NEt_4_^+^ by metallocage **1**.

In the view to develop a computational framework able to describe the encapsulation of guest molecules into metallocages, we extended our study to the calculation of the Gibbs energy barriers of the encapsulation process. With this purpose, we used an umbrella sampling approach^63^ and calculated the PMF associated with the entrance of a guest in the metallocage **1**. Such kind of simulations are computationally much more demanding than those to obtain binding Gibbs energies, and we thus decided to focus on the encapsulation of NEt_4_^+^, **2**, in the metallocage (system **2** ⊂ **1**; Figure 5). This approach allows a thorough exploration of the mechanism of guest encapsulation under realistic conditions. The variable selected as the reaction coordinated to analyze the encapsulation process was the distance between the centers of mass of the host and guest (see computational details).

The encapsulation process of guest **2** in a macromolecular host **1** can be segmented in two steps. The first event consists in the formation of an ion-pair complex (Figure 5: ion-pair intermediate) by the association of the cationic guest, NEt_4_^+^, with the metallocage from the two isolated species in solution (Figure 5: free guest state). This event was experimentally detailed by analyzing the host–guest encapsulation equilibria.^75^ In this state, the guest remains on the outer surface of the highly anionic host, [Ga_4_L_6_]^12–^. This process is exothermic due to strong attractive interactions (as observed in the Gibbs energy profile). The second event consists in the penetration of the guest from exterior into the internal cavity of the metallocage (Figure 5: from the ion-pair to encapsulated state). This step is favorable, although with a barrier associated with the entry of the cationic guest into the metallocage.

**Figure 6:**
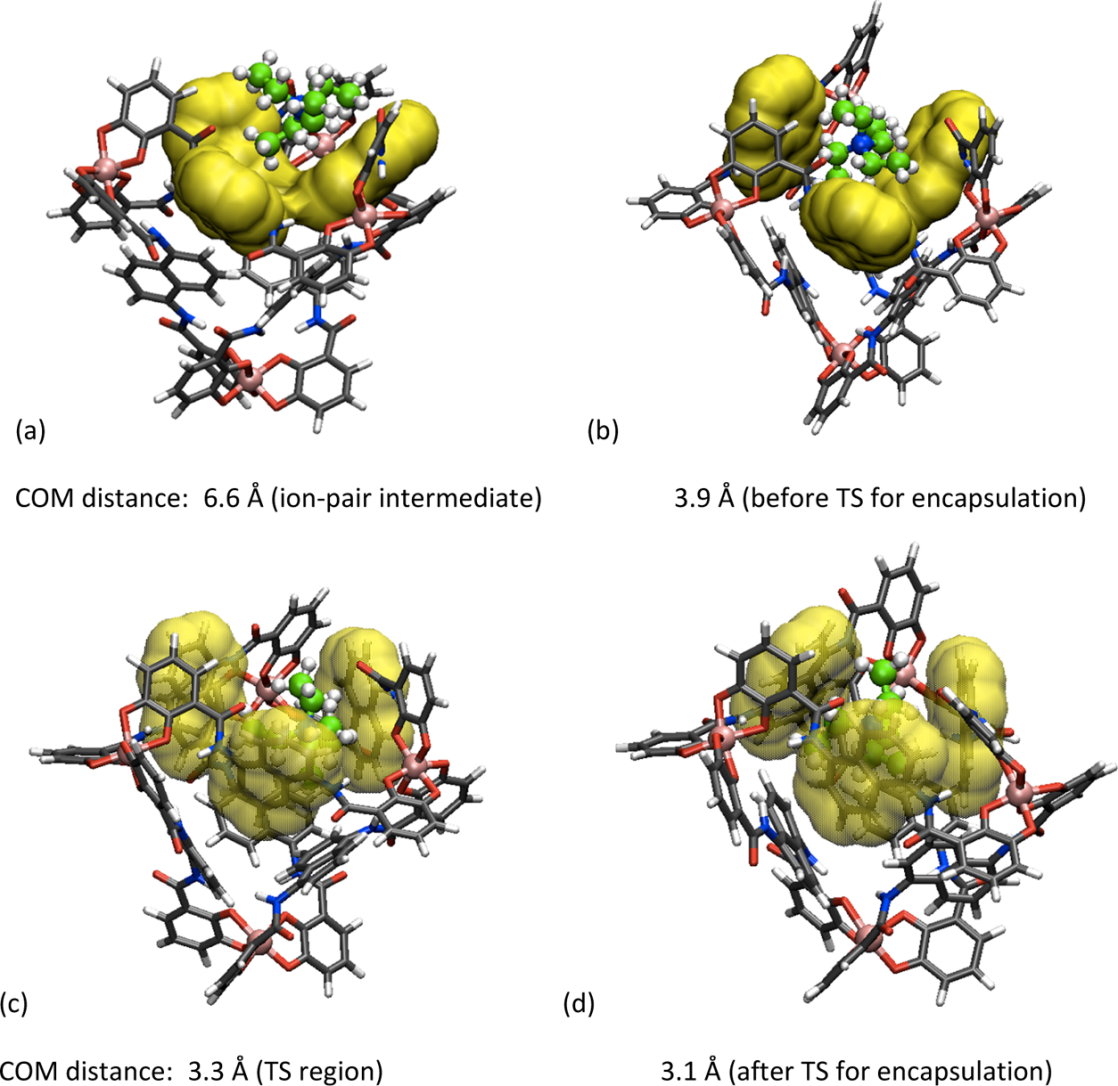
Representative molecular structures of the encapsulation of the guest into the metallocage. The solvent molecules are not shown for clarity. COM = center of mass.

The flexibility of the metallocage has been demonstrated by the large variability of the size of the cavity shown during the MD simulations, as well as by encapsulating guests with different sizes and shapes inside the cavity. During the guest release and encapsulation, the deformation of the metallocage by movements of six naphthalene-based bisbidentate catechol ligands has been proposed, but the process has not been yet described at the atomic level in explicit solvents for any guest. Therefore, we intended to discuss this process in an illustrative way from MD with an explicit solvent and counter ions. The general representation of the process is shown by the most populated molecular structures in Figure 6 (a different view is given in Figure S8).

In the free guest state (Figure 5), at a distance between the COM of the metallocage and the COM of the guest around 15.0 Å, the metallocage and the guest are both fully solvated. The ion-pair intermediate (NEt_4_^+^·[Ga_4_L_6_]^12–^) forms at a distance of ∼6.6 Å between their respective centers of masses (Figure 5). A representative geometry of this intermediate is shown in Figure 6a. In this intermediate, the guest is positioned close to one of the four triangular faces of the tetrahedron host, which is formed by three naphthalene rings of the metallocage and shaped as a “concave cup” (Figure 6a). This is the most stable place for NEt_4_^+^ outside the metallocage in aqueous solution. The interactions between the host and the guest can be described as C–H···π interactions between the C–H bonds of the guest and the naphthalene rings of the metallocage and C–H···O(amide) interactions between the C–H bonds of the guest and the amide oxygen of the metallocage. Moreover, the overall charge is also at play. Interestingly, the internal cavity of the metallocage at this stage is surprisingly small, with a volume lower than 30 Å^3^ (average of the cavity volumes of the three most populated structures). This is due to the fact that the naphthalene rings rotate to accommodate the guest, provoking that the inside cavity shrinks considerably (with no other molecules inside the cavity). Nevertheless, as previously shown, the system is dynamic and this volume fluctuates. The calculated Gibbs energy associated with this ion-pair intermediate state is −3.4 kcal/mol relative to the free guest state (Figure 5); this is in excellent agreement with the estimated value of −3.5 kcal/mol from the experimental observations.^75^

The following step is the encapsulation of the guest from the ion-pair state into the internal cavity of the metallocage (Figure 5: from ion-pair to encapsulated state). When the guest passes through the ion-pair intermediate state to the transition state for encapsulation, the “concave-cup” structure of the face of the metallocage changes in shape by rotational movements of the naphthalene rings of the metallocage. To illustrate this, we selected a point where the distance between the COM of the metallocage and the COM of the guest, NEt_4_^+^, is 3.9 Å (0.6 Å prior to the transition state for encapsulation; Figure 6b). At this point, the cavity of the metallocage is calculated to be 233 Å^3^ (calculated for a representative structure of the most populated conformation; it includes the zone comprised among the three naphthalene rings; Figure S9). This shows that the rotation of the naphthalene rings significantly modifies the accessible cavity of the metallocage.

**Figure 7:**
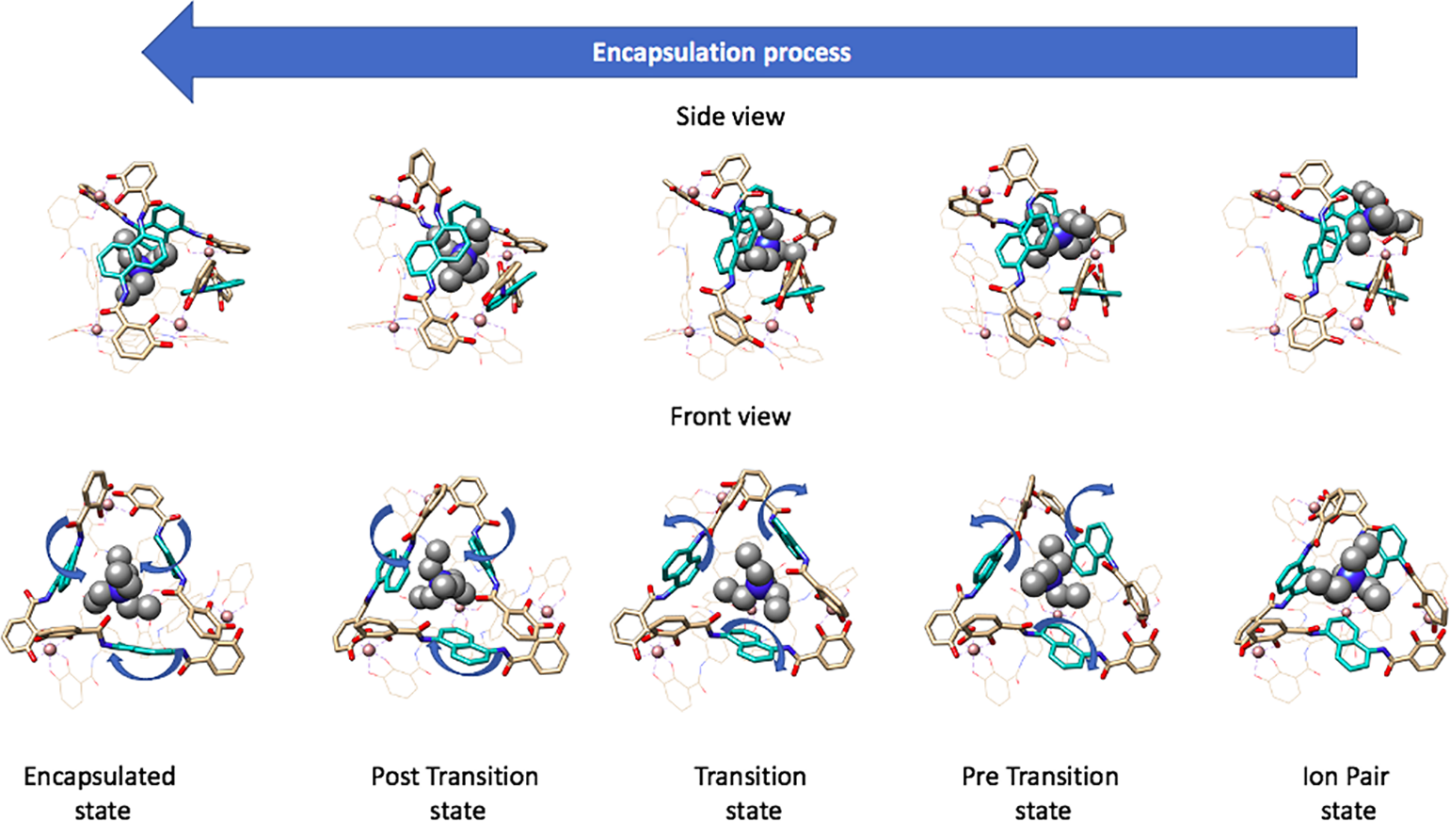
Schematic view of the molecular motions of the encapsulation process from the ion-pair state (right) to encapsulated state (left). Branches of the metallocages involved in controlling guest entrance are represented in stick with the naphthalene groups in cyan. Branches from the opposite side of the cage and with little implication in the transitional motion are represented in wires. The guest molecule is represented in spheres.

The transition-state region for the encapsulation is calculated to be at the distance of around 3.3 Å between the COM of the metallocage and the COM of NEt_4_^+^ (a representative snapshot is shown in Figure 6c). This separation is about half of the distance observed for the ion-pair intermediate. The three naphthalene rings of the metallocage are positioned nearly parallel to each other. They still keep C–H···π interactions and the C–H···O(amide) interactions with NEt_4_^+^ as in the ion-pair intermediate state. The cavity volume of the metallocage is ∼350 Å^3^, indicating that the cavity increases at the transition state. For comparison, we also calculated the volume of the cavity at a distance 3.1 Å after the transition state (Figure 6d), which turned out to be 238 Å^3^, showing that the cavity shrinks significantly after the transition state (Figure S9). At this point, we observed that one of the naphthalene rings of the metallocage rotates in the closing direction of the guest-entering face of the tetrahedron host (Figure 6d). A representation of the whole motion for the encapsulation process is shown in Figure 7.

After the guest passes through the transition state for encapsulation, the encapsulated state is observed at a distance of 1.1 Å between the COM of the metallocage and the COM of NEt_4_^+^. This indicates that the guest does not sit at the center of the cavity, and it relays closer to one of the host walls. In the encapsulated state, the volume of the cavity of the metallocage is 272 Å^3^ (calculated for a representative structure of the most populated conformation). There is a solvent water molecule found inside the cavity of the metallocage along with NEt_4_^+^; the calculated packing coefficient is 58%, which is quite close to Rebek’s 55% rule^74^ and the classical plain MD result (56%). This shows that the cage is able to vary its volume, trying to keep the packing coefficient close to the Rebek rule.

**Figure 8:**
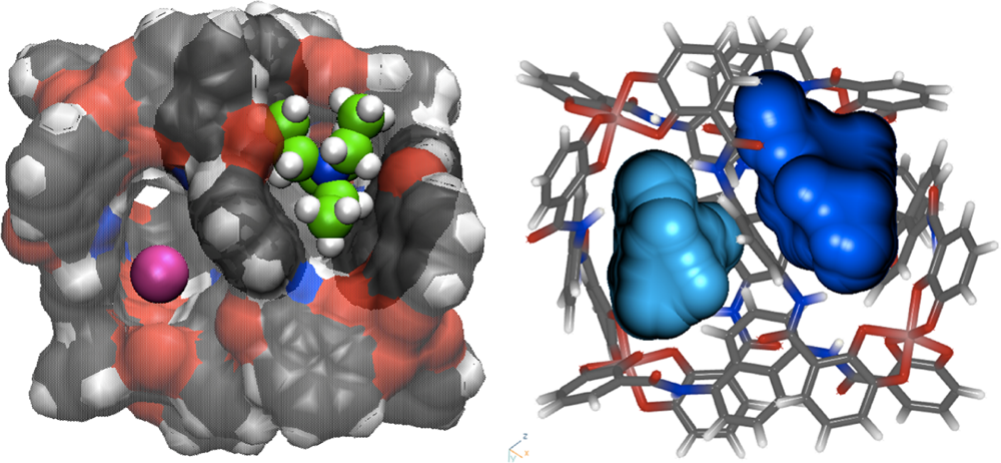
Two different representations of the cavities generated during the encapsulation of NEt^4+^. They are taken from a snapshot of the most populated structure of MD simulations at a COM distance of 5.1 Å. K^+^ in purple.

A closer look on the encapsulation process shows a transient state where the metallocage generates two cavities separated by one of the naphthalene-based ligands. The distance between COMs of the host and guest is 5.1 Å, which is a state between the ion-pair intermediate (6.6 Å) and the transition state (3.3 Å). The two cavities, with volumes of 129 and 188 Å^3^ are shown in Figure 8, in cyan and blue, respectively. Interestingly, one of the cavities (in cyan in Figure 8) includes K^+^ with three solvent water molecules, while the other (in blue in Figure 8) is occupied by the guest, NEt_4_^+^.

The presence of the potassium at this particular step of the encapsulation process prompted us to hypothesize a mechanistic role of this ion and a possible exchange between K^+^ (inside the cage) with the guest (outside the cage). Nevertheless, the analysis of the distribution of the K^+^ cations shows that during most of the simulation time, there are no K^+^ cations inside the cavity (Figure S13) and that the presence of K^+^ in one of the cavities formed during migration of the ligand is more likely to be due to the proximity of the ions from the cage during the simulation. Our work suggests therefore that the mechanism proceeds by a simple encapsulation of the guest to the metallocage more than an ion–ligand exchange process.

The Gibbs energy barrier for encapsulation is calculated to be 2.4 kcal/mol from the ion-pair intermediate (Figure 5), whereas it is 3.8 kcal/mol for the guest releasing from the encapsulated state. The encapsulated state is more stable than the ion-pair intermediate by 1.4 kcal/mol, which is in very good agreement with 2.7 kcal/mol obtained from the experiment.^75^ Moreover, the encapsulation of NEt_4_^+^ within the metallocage is found to be a spontaneous process with a rather low barrier. This is in line with the displacement experiment: encapsulated NMe_4_^+^ in the metallocage **1** is readily displaced by NEt_4_^+^, and the process is too fast to be monitored by ^1^H NMR spectroscopy.^9^

From a computational point of view, the binding Gibbs energy estimated from the PMF (ca. −5.0 kcal/mol) is consistent with the APR result (−6.3 kcal/mol). The small difference is likely due to the different sampling strategies, probably limited in the umbrella sampling simulation at large host–guest separation.

## 4 Conclusions

This study shows that classical MD in an explicit solvent with proper parameters provides the level of accuracy required for analyzing the encapsulation of cationic species by a supramolecular metallocage. Importantly, for the sake of future modeling experiments on such kinds of systems, here, the best results were obtained when deriving the force field parameters using QM calculation, accounting for solvent effects implicitly. The binding Gibbs energies were calculated to be in very good agreement with the experiment.

The binding energies of different guests to the metallocage were found to correlate very well with the volume of the guest (*V*_guest_) and the *V*_guest_/*V*_cavity_ ratio. Interestingly, these parameters are available with low computational efforts. These correlations showed an outlier among the guests considered. It is the one that does not fit into the cavity, lying partially outside, despite showing good packing predictions. Calculated packing coefficients for the guests are in the range of 0.55–0.63, close to Rebek’s proposal of 0.55, provided that the solvent molecules inside the cavity are included in their evaluation, as revealed by the MD simulations. This work also sustains the validity of Rebek’s rule as a general trend to predict favorable encapsulation and also that atomistic modeling such as the one performed here can provide with information on when this rule reaches its limitations (mainly related to the shape and size host–guest complementarity).

The simulations allowed obtaining the atomic description for the encapsulation process. The encapsulation of the guest into the metallocage takes place in two steps. First, an outer surface ion-pair intermediate forms: the guest faces one of the tetrahedron’s sides of the host, which turns concave due to the conformation adopted by the naphthalene-base ligands. Then, entering of the guest into the metallocage is regulated by the rotational movements of three naphthalene ligands of the metallocage; rotation of a naphthalene ligand creates two separate cavities. After the guest passes through the transition state, the metallocage closes the aperture and interacts with the guest by its interior surface. In the guest-encapsulated state, water molecules can be encapsulated along with the guest depending on the guest’s size, shape, and interactions.

Overall, properly derived force field parameters in combination with statistical mechanics methods allow computing host–guest binding energies for SOCs along with providing an atomic description of the encapsulation process. This offers an interesting option for future development in MOC design.

## Supporting Information Available

Data for analyzing the MD trajectories; plots with correlations of Gibbs binding energies; figures of the encapsulation process; derived force field parameters; and Cartesian coordinates for the optimized structures (PDF)

## Notes

Metal Center Parameter Builder;^36^ AMBER 16 Package;^56^ (https://ambermd.org) Gaussian09 Program, Revision D.01;^48^ (https://gaussian.com); the WHAM Method^64^ (http://membrane.urmc.rochester.edu/wordpress/?page_id=126)

## Abbreviations

DFT: density functional theory
QM: quantum mechanics
B3LYP-D3: dispersion-corrected Becke three-parameter Lee–Yang–Parr exchange–correlation functional
SMD: solvent model density
PCM: polarizable continuum model
quasi-RRHO: quasi-rigid-rotor-harmonic-oscillator approximation
MD: molecular dynamics
MCPB.py: Python-based metal center parameter builder program
RESP: restrained electrostatic potential method
WHAM: weighted histogram analysis method
