# Supplementary material for: Modeling Kinetics and Thermodynamics of Guest Encapsulation into the [M4L6]12– Supramolecular Organometallic Cage
Source: J Chem Inf Model. 2021 Sep 10;61(9):4370–81. doi: 10.1021/acs.jcim.1c00348 (PMC8479806; doi:10.1021/acs.jcim.1c00348)
Supplement: Supplementary file 1 — ci1c00348_si_001.pdf [file ci1c00348_si_001.pdf]

**Supporting Information**

# Modeling Kinetics and Thermodynamics of Guest Encapsulation into $[M_4L_6]^{12-}$ Supramolecular Organometallic Cage

Gantulga Norjmaa,[a] Pietro Vidossich,[b] Jean-Didier Maréchal[a]* and Gregori Ujaque[a]*

[a]*Departament de Química and Centro de Innovación en Química Avanzada (ORFEO-CINQA), Universitat Autònoma de Barcelona, Cerdanyola del Valles, 08193 Barcelona, Catalonia, Spain*
[b] *Laboratory of Molecular Modeling and Drug Discovery, Istituto Italiano di Tecnologia, Via Morego 30, 16163, Genova, Italy*

Contents:

1. Behavior of the metallocage 1 in solution …… S2
2. Correlations for binding energies …… S3
3. Snapshots of the APR simulations …… S5
4. Analysis of the encapsulation process …… S6
5. APR simulation detail and setup …… S8
6. Force field parameters and charges derived in implicit water solvent and in gas phase …… S10
7. Cartesian coordinates of the metallocage and $NEt_4^+$ encapsulated in the metallocage optimized at DFT level …… S26

## 1. Behavior of the metallocage 1 in solution

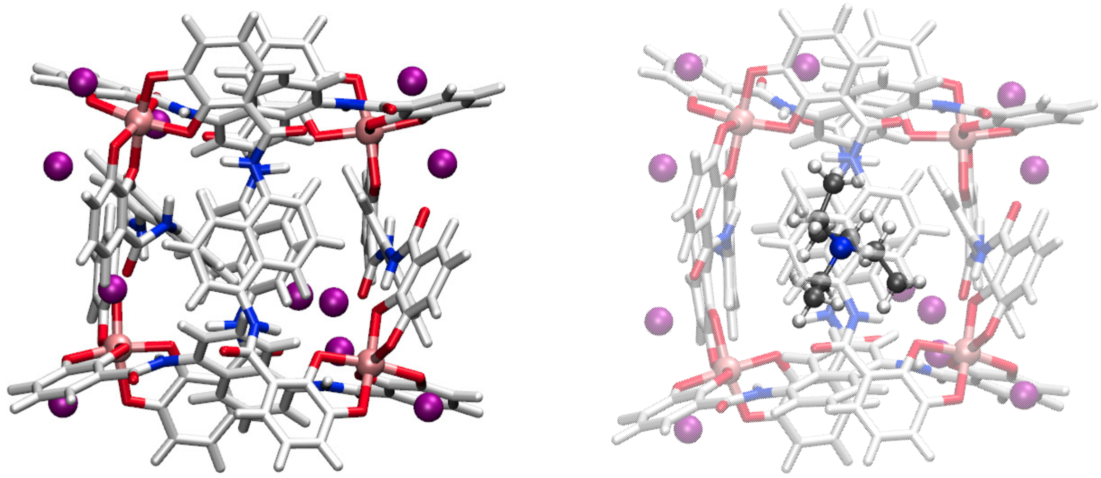

(a) (b)

**Figure S1**. Optimized geometries of (a) metallocage, **1**, and (b) $NEt_4^+$ encapsulated in the metallocage with implicit solvent. In both cases, 11 $K^+$ ions (purple spheres) were added explicitly in order to neutralize the system.

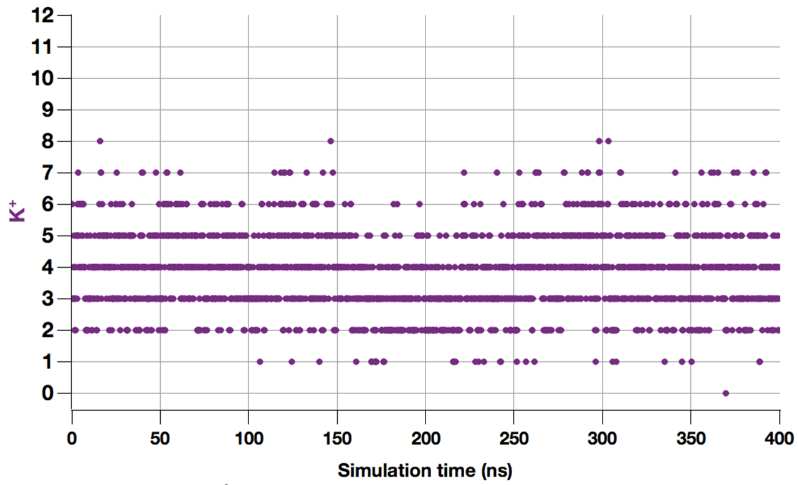


**Figure S2**. The number of $K^+$ < 11 Å from the center of mass of the metallocage **1** during classical molecular dynamic simulation.

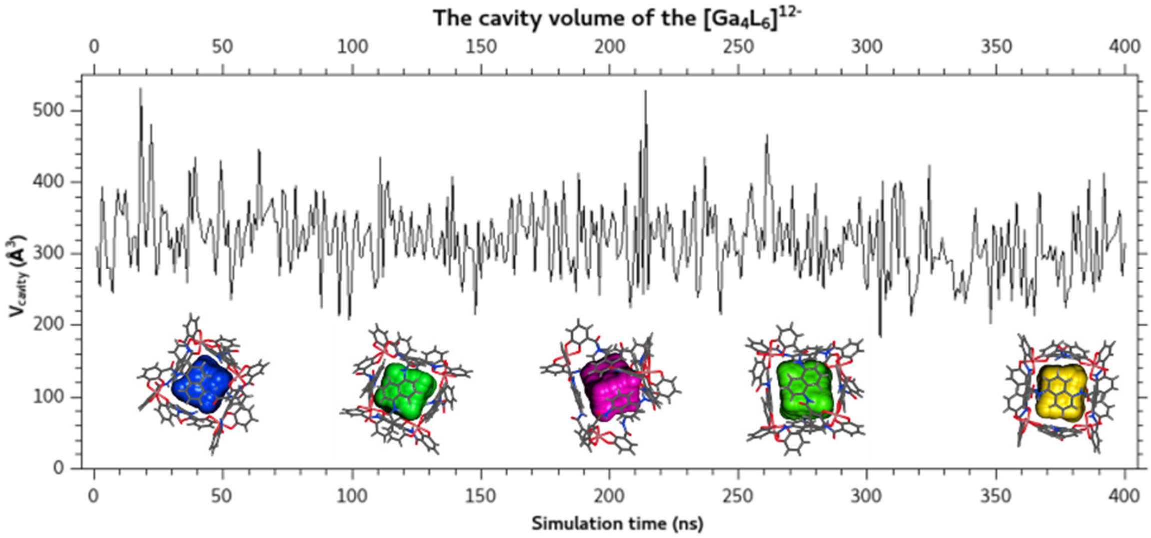


**Figure S3**. Cavity volume of the **2** ⊂ **1** system in water solvent.

## 2. Correlations for binding energies

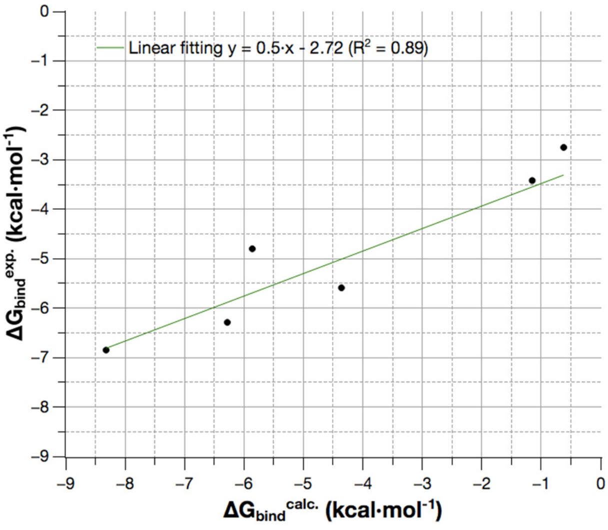


**Figure S4**. Plot of the computed vs experimental binding Gibbs energies.

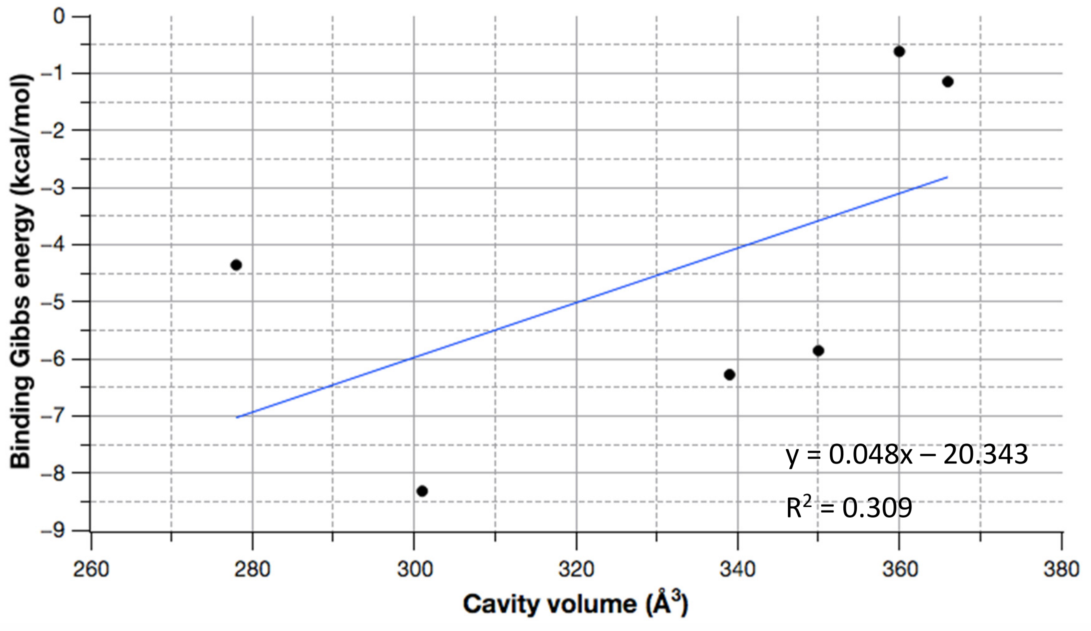


**Figure S5.** Correlation between binding Gibbs energy and the cavity volume of the metallocage of the host-guest complexes.

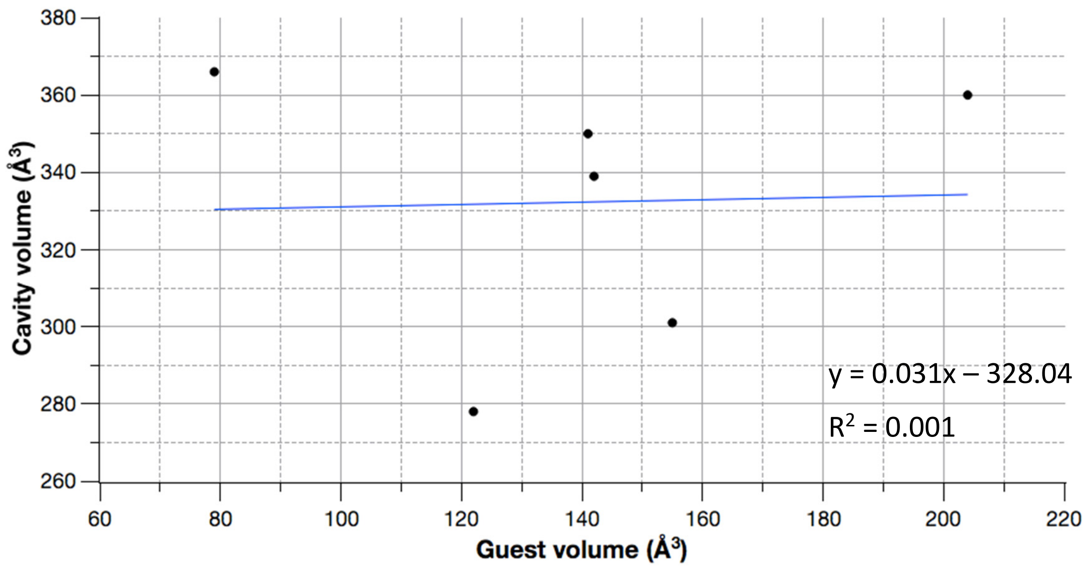


**Figure S6**. Correlation between binding Gibbs energy and the guest volume of the metallocage of the host-guest complexes.

**Table S1**. Evaluation of the average number of water molecules inside the cavity along with the substrate, $V_{cavity}$ and packing coefficients for the guests+solvent encapsulated system.

| Host-guest complexes | | $V_{guest}$ | 200ns of classical plain MDs | | |
|---|---|---|---|---|---|
| | | | H2O inside | V cavity | PC % |
| **2 in 1** | $[Et_4N]^+$ | 142.2 | 2 | **324** | **56%** |
| **3 in 1** | $[Me_4N]^+$ | 78.61 | 4 | **279** | **56%** |
| **4 in 1** | $[Et_4P]^+$ | 154.6 | 2 | **306** | **63%** |
| **5 in 1** | $[5spiro]^+$ | 122.4 | 3 | **297** | **61%** |
| **6 in 1** | $[Pr_4N]^+$ | 204 | 0 | 456 | 45% |
| **7 in 1** | $[Me_2Pr_2N]^+$ | 141.3 | 2 | **325** | **55%** |

## 3. Snapshots of the APR simulations

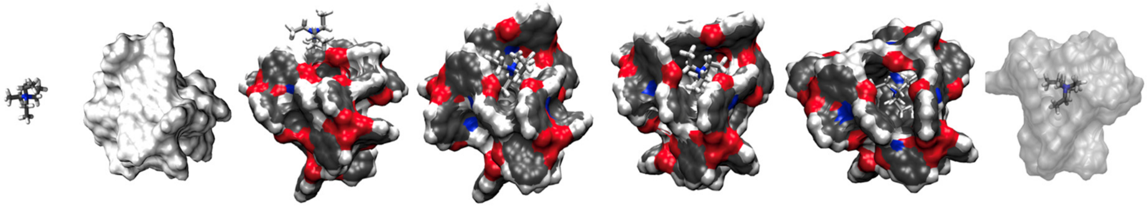

**Figure S7**. MD snapshots from APR simulations of **2** ⊂ **1**.

## 4. Analysis of the encapsulation process

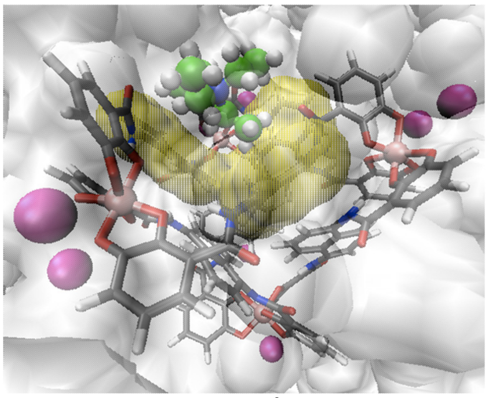

(a) COM distance: 6.6 Å (ion-pair intermediate)

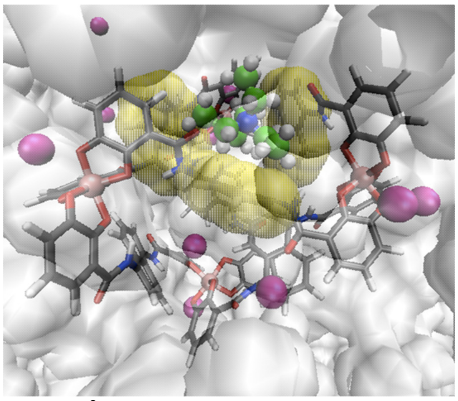

(b) 3.9 Å (before TS for encapsulation)

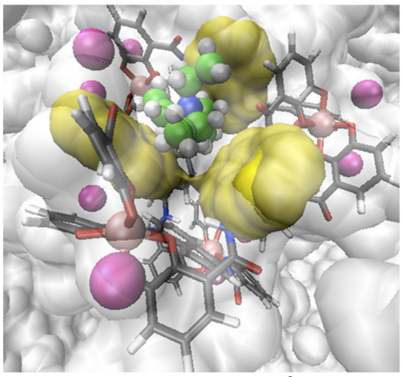

(c) COM distance: 3.3 Å (TS region)

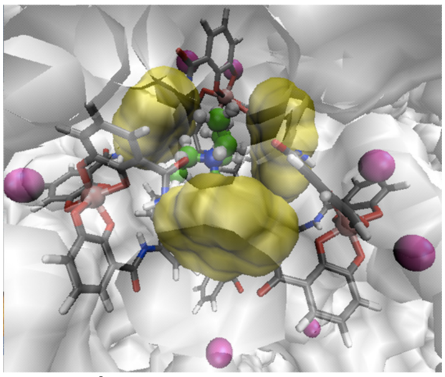

(d) 3.1 Å (after TS for encapsulation)

**Figure S8**. Encapsulation event of **2** into the metallocage **1** in water solvent. $K^+$ counter ions are in purple sphere and explicit solvent water molecules are in white surface.

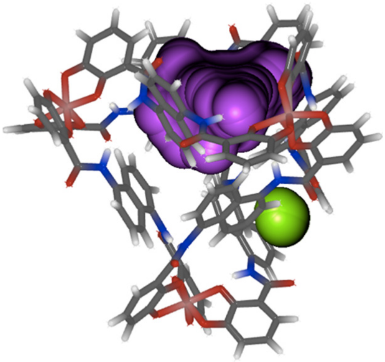

Cavity volume: 233 Å$^3$ / 15 Å$^3$
Packing coefficient: 61%
Encapsulated system: Guest

**(a) before TS**

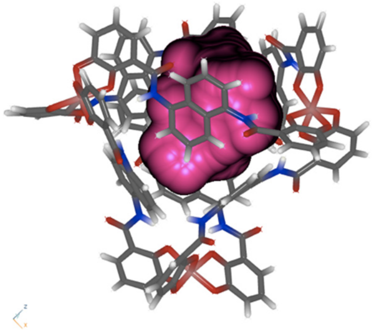

Cavity volume: 346 Å$^3$
Packing coefficient: 41%
Encapsulated system: guest

**(b) TS region**

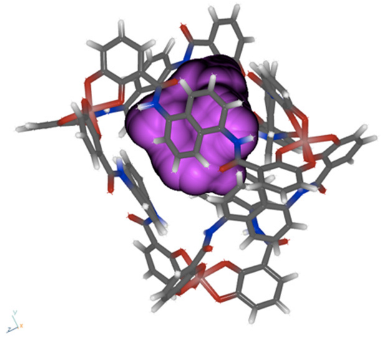

Cavity volume: 238 Å$^3$
Packing coefficient: 59%
Encapsulated system: guest

**(c) after TS region**

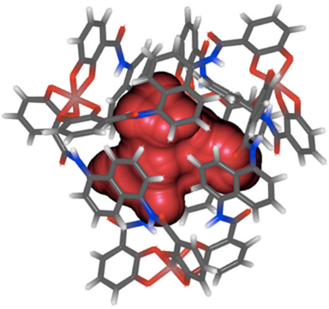

Cavity volume: 272 Å$^3$
Packing coefficient: 57%
Encapsulated system: guest/$H_2O$

**(d) encapsulated state**

**Figure S9**. Cavity volumes and packing coefficients of the metallocage during the encapsulation process.

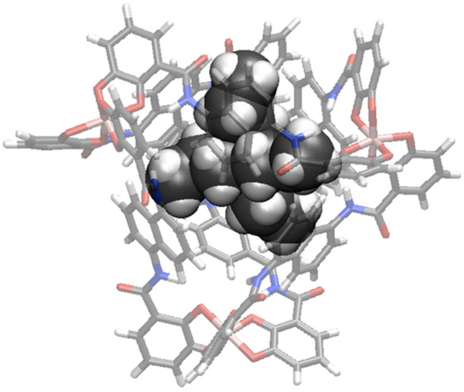

**Figure S10.** Snapshot of the most populated structure of the encapsulated state of **6** $\subset$ **1** complex from APR simulations for binding free energy calculations

**Table S2**. Comparison of calculated ΔΔG.

| Guest relative to $NEt_4^+$ | APR | Experiment | FEP | Full QM |
|---|---|---|---|---|
| | ΔΔG | ΔΔG | ΔΔG | ΔΔE |
| $NMe_4^+$ | 5.13 ± 0.99 | 2.87 ± 0.01 | 6.30 ± 0.05 | 15.9 |
| $PEt_4^+$ | -2.04 ± 0.84 | -0.64 ± 0.04 | -0.27 ± 0.01 | -1.6 |
| $NPr_4^+$ | 5.66 ± 0.92 | 3.46 ± 0.04 | 5.07 ± 0.10 | -0.3 |
| $NMe_2Pr_2^+$ | 0.42 ± 1.12 | 1.41 ± 0.04 | 3.19 ± 0.11 | 4.9 |

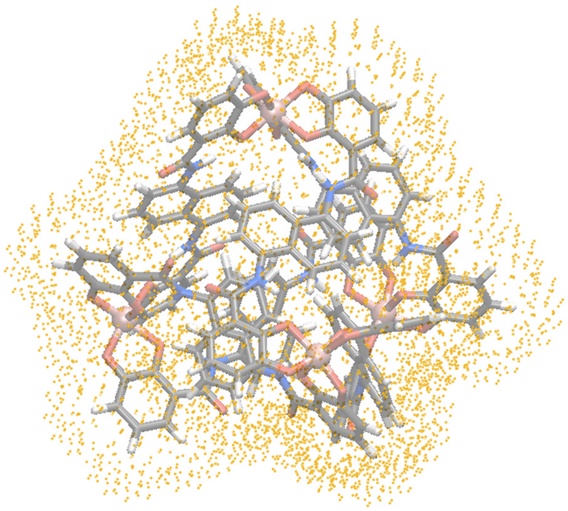

**Figure S11**. Calculated ESP centers (yellow dots) obtained from QM ESP calculation.

## 5. APR simulation detail and setup

MD simulations in each APR window:
1. Minimizing 50000 cycles,
2. Running 1ps NVT at 10 K,
3. Heating the system from 10 K to 298.15 K in 100 ps,
4. Equilibrating the system under constant pressure, 50 NPT cycle,
5. NPT Production from 2.5 ns to 25 ns depending on the standard error of the mean (SEM) of the restraint forces, the SEM threshold is 0.100.

The cutoff for non-bonding interactions: 9 Å
The approach employed for computing the electrostatic forces and potential: The Particle Mesh Ewald (PME) method
The type of thermostat: Langevin
The type of barostat: Monte Carlo
The time step: 4 fs (hydrogen mass repartitioning was used)

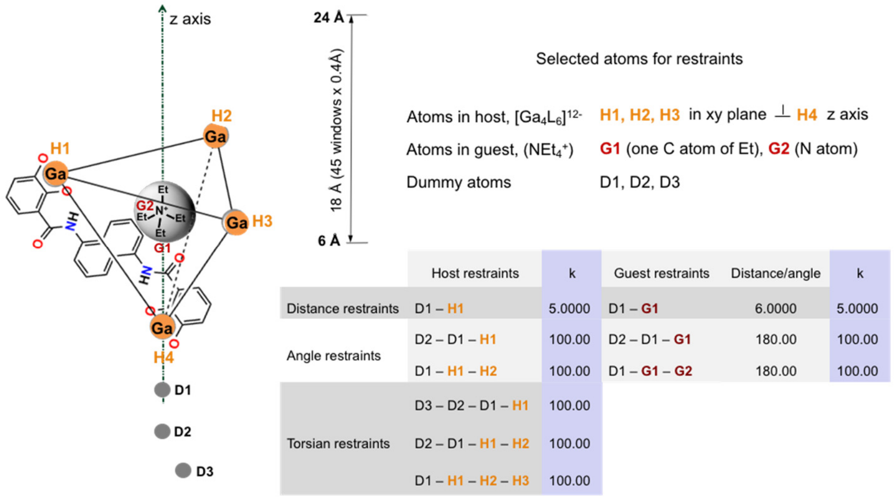


**Figure S12**. Schematic representation of the APR simulations in this study.

**Table S3**. Comparison between calculated binding Gibbs energies of the $NEt_4^+$ in the metallocage obtained using atomic charges derived with implicit solvent and without implicit solvent.

| Atomic charges of $[Ga_4L_6]^{12-}$ and $NEt_4^+$ | | |
|---|---|---|
| Geometry optimization in | implicit solvent | implicit solvent |
| Charge derivation in | implicit solvent | vacuum |
| Calculated $\Delta G^o_{bind}$ | -6.3 ± 0.6 | 2.7 ± 0.5 |
| Experimental $\Delta G^o_{bind}$ | -6.2 ± 0.01 | |

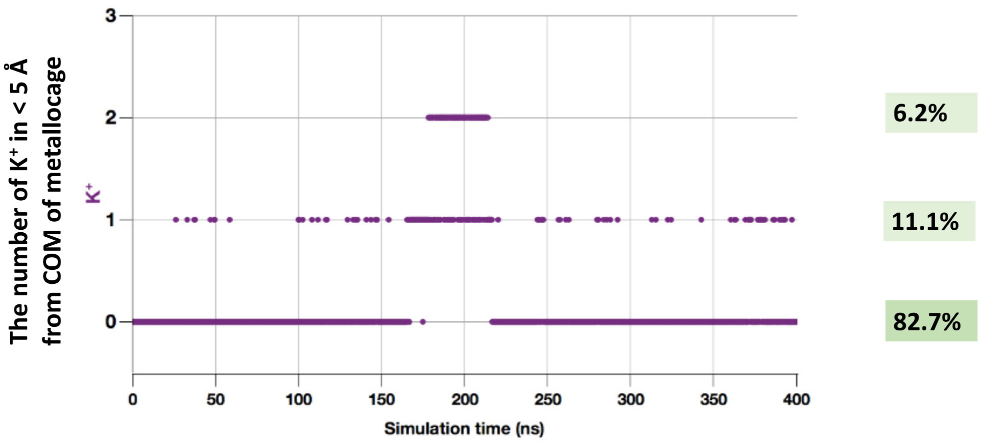


**Figure S13**. The number of $K^+$ in less than 5 Å from the center of mass of the metallocage during 400 ns classical molecular dynamic simulation of the metallocage.

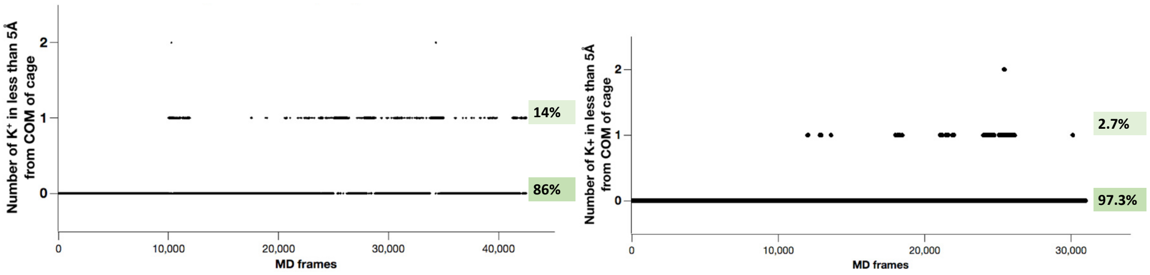


(a) (b)

**Figure S14**. The number of $K^+$ in less than 5 Å from the center of mass of the metallocage (a) during umbrella sampling simulations and (b) during the APR simulations.

## 6. Force field parameters and charges derived in implicit water solvent

BOND

| | | | |
|---|---|---|---|
| A1-M4 | 52.7 | 1.9974 | Created by Seminario method using MCPB.py |
| A2-M4 | 24.6 | 2.0712 | Created by Seminario method using MCPB.py |
| A3-M4 | 46.8 | 2.0187 | Created by Seminario method using MCPB.py |
| A4-M4 | 46.9 | 2.0131 | Created by Seminario method using MCPB.py |
| A5-M4 | 40.9 | 2.0296 | Created by Seminario method using MCPB.py |
| A6-M4 | 60.4 | 1.9850 | Created by Seminario method using MCPB.py |
| Y1-M1 | 50.2 | 2.0040 | Created by Seminario method using MCPB.py |
| Y2-M1 | 44.0 | 2.0236 | Created by Seminario method using MCPB.py |
| Y3-M1 | 43.7 | 2.0256 | Created by Seminario method using MCPB.py |
| Y4-M1 | 57.5 | 1.9869 | Created by Seminario method using MCPB.py |
| Y5-M1 | 51.3 | 1.9990 | Created by Seminario method using MCPB.py |
| Y6-M1 | 31.0 | 2.0520 | Created by Seminario method using MCPB.py |
| Y7-M2 | 28.2 | 2.0631 | Created by Seminario method using MCPB.py |
| Y8-M2 | 57.5 | 1.9891 | Created by Seminario method using MCPB.py |
| Y9-M2 | 28.2 | 2.0633 | Created by Seminario method using MCPB.py |
| Z1-M2 | 60.6 | 1.9835 | Created by Seminario method using MCPB.py |
| Z2-M2 | 48.2 | 2.0048 | Created by Seminario method using MCPB.py |
| Z3-M2 | 52.8 | 2.0065 | Created by Seminario method using MCPB.py |
| Z4-M3 | 22.2 | 2.0778 | Created by Seminario method using MCPB.py |
| Z5-M3 | 51.0 | 2.0026 | Created by Seminario method using MCPB.py |
| Z6-M3 | 46.7 | 2.0180 | Created by Seminario method using MCPB.py |
| Z7-M3 | 41.7 | 2.0263 | Created by Seminario method using MCPB.py |
| Z8-M3 | 65.9 | 1.9725 | Created by Seminario method using MCPB.py |
| Z9-M3 | 41.5 | 2.0321 | Created by Seminario method using MCPB.py |
| ca-A1 | 598.1 | 1.2358 | SOURCE4_SOURCE5 17 0.0088 |

ANGL

| | | | |
|---|---|---|---|
| A1-M4-A2 | 85.30 | 81.38 | Created by Seminario method using MCPB.py |
| A1-M4-A3 | 33.20 | 95.49 | Created by Seminario method using MCPB.py |

| | | | |
|---|---|---|---|
| A1-M4-A4 | 60.35 | 89.89 | Created by Seminario method using MCPB.py |
| A1-M4-A5 | 65.00 | 170.39 | Created by Seminario method using MCPB.py |
| A1-M4-A6 | 46.37 | 94.74 | Created by Seminario method using MCPB.py |
| A2-M4-A3 | 46.08 | 83.68 | Created by Seminario method using MCPB.py |
| A2-M4-A4 | 85.81 | 161.51 | Created by Seminario method using MCPB.py |
| A2-M4-A5 | 46.96 | 90.09 | Created by Seminario method using MCPB.py |
| A2-M4-A6 | 49.11 | 95.05 | Created by Seminario method using MCPB.py |
| A3-M4-A4 | 74.59 | 80.95 | Created by Seminario method using MCPB.py |
| A3-M4-A5 | 48.24 | 87.89 | Created by Seminario method using MCPB.py |
| A3-M4-A6 | 55.36 | 169.38 | Created by Seminario method using MCPB.py |
| A4-M4-A5 | 51.64 | 99.53 | Created by Seminario method using MCPB.py |
| A4-M4-A6 | 68.53 | 101.93 | Created by Seminario method using MCPB.py |
| A5-M4-A6 | 70.35 | 81.56 | Created by Seminario method using MCPB.py |
| Y1-M1-Y2 | 76.55 | 81.72 | Created by Seminario method using MCPB.py |
| Y1-M1-Y3 | 20.08 | 170.54 | Created by Seminario method using MCPB.py |
| Y1-M1-Y4 | 67.84 | 96.08 | Created by Seminario method using MCPB.py |
| Y1-M1-Y5 | 39.55 | 95.47 | Created by Seminario method using MCPB.py |
| Y1-M1-Y6 | 32.02 | 91.28 | Created by Seminario method using MCPB.py |
| Y2-M1-Y3 | 45.05 | 89.22 | Created by Seminario method using MCPB.py |
| Y2-M1-Y4 | 32.10 | 98.02 | Created by Seminario method using MCPB.py |
| Y2-M1-Y5 | 61.22 | 167.06 | Created by Seminario method using MCPB.py |
| Y2-M1-Y6 | 46.72 | 85.64 | Created by Seminario method using MCPB.py |
| Y3-M1-Y4 | 73.41 | 82.43 | Created by Seminario method using MCPB.py |
| Y3-M1-Y5 | 21.45 | 93.97 | Created by Seminario method using MCPB.py |
| Y3-M1-Y6 | 40.16 | 90.73 | Created by Seminario method using MCPB.py |
| Y4-M1-Y5 | 37.28 | 94.82 | Created by Seminario method using MCPB.py |
| Y4-M1-Y6 | 42.68 | 172.17 | Created by Seminario method using MCPB.py |
| Y5-M1-Y6 | 81.09 | 81.79 | Created by Seminario method using MCPB.py |
| Y7-M2-Y8 | 67.89 | 80.83 | Created by Seminario method using MCPB.py |
| Y7-M2-Y9 | 49.28 | 88.39 | Created by Seminario method using MCPB.py |
| Y7-M2-Z1 | 62.05 | 169.59 | Created by Seminario method using MCPB.py |
| Y7-M2-Z2 | 42.05 | 97.99 | Created by Seminario method using MCPB.py |
| Y7-M2-Z3 | 26.10 | 86.04 | Created by Seminario method using MCPB.py |
| Y8-M2-Y9 | 42.08 | 92.20 | Created by Seminario method using MCPB.py |
| Y8-M2-Z1 | 35.35 | 95.26 | Created by Seminario method using MCPB.py |
| Y8-M2-Z2 | 67.37 | 101.06 | Created by Seminario method using MCPB.py |
| Y8-M2-Z3 | 31.38 | 166.77 | Created by Seminario method using MCPB.py |
| Y9-M2-Z1 | 76.01 | 82.10 | Created by Seminario method using MCPB.py |
| Y9-M2-Z2 | 58.48 | 166.03 | Created by Seminario method using MCPB.py |
| Y9-M2-Z3 | 37.98 | 85.86 | Created by Seminario method using MCPB.py |
| Z1-M2-Z2 | 52.17 | 92.21 | Created by Seminario method using MCPB.py |
| Z1-M2-Z3 | 27.43 | 97.42 | Created by Seminario method using MCPB.py |
| Z2-M2-Z3 | 72.57 | 82.22 | Created by Seminario method using MCPB.py |
| Z4-M3-Z5 | 78.09 | 80.16 | Created by Seminario method using MCPB.py |
| Z4-M3-Z6 | 42.67 | 82.80 | Created by Seminario method using MCPB.py |
| Z4-M3-Z7 | 78.68 | 160.54 | Created by Seminario method using MCPB.py |
| Z4-M3-Z8 | 36.45 | 98.03 | Created by Seminario method using MCPB.py |
| Z4-M3-Z9 | 47.53 | 97.04 | Created by Seminario method using MCPB.py |

Z5-M3-Z6 37.52 95.08 Created by Seminario method using MCPB.py
Z5-M3-Z7 55.98 90.12 Created by Seminario method using MCPB.py
Z5-M3-Z8 63.21 94.85 Created by Seminario method using MCPB.py
Z5-M3-Z9 36.09 175.87 Created by Seminario method using MCPB.py
Z6-M3-Z7 76.87 81.31 Created by Seminario method using MCPB.py
Z6-M3-Z8 47.46 170.03 Created by Seminario method using MCPB.py
Z6-M3-Z9 45.11 87.55 Created by Seminario method using MCPB.py
Z7-M3-Z8 35.88 99.57 Created by Seminario method using MCPB.py
Z7-M3-Z9 33.09 93.43 Created by Seminario method using MCPB.py
Z8-M3-Z9 74.00 82.49 Created by Seminario method using MCPB.py
ca-A1-M4 35.55 111.15 Created by Seminario method using MCPB.py
ca-A2-M4 44.38 109.50 Created by Seminario method using MCPB.py
ca-A3-M4 43.98 108.91 Created by Seminario method using MCPB.py
ca-A4-M4 43.95 108.23 Created by Seminario method using MCPB.py
ca-A5-M4 42.66 109.02 Created by Seminario method using MCPB.py
ca-A6-M4 47.78 109.36 Created by Seminario method using MCPB.py
ca-Y1-M1 43.33 108.83 Created by Seminario method using MCPB.py
ca-Y2-M1 44.20 109.03 Created by Seminario method using MCPB.py
ca-Y3-M1 46.94 111.35 Created by Seminario method using MCPB.py
ca-Y4-M1 50.55 111.61 Created by Seminario method using MCPB.py
ca-Y5-M1 45.06 111.07 Created by Seminario method using MCPB.py
ca-Y6-M1 46.19 109.81 Created by Seminario method using MCPB.py
ca-Y7-M2 49.70 108.22 Created by Seminario method using MCPB.py
ca-Y8-M2 44.57 109.14 Created by Seminario method using MCPB.py
ca-Y9-M2 55.01 111.07 Created by Seminario method using MCPB.py
ca-Z1-M2 52.88 112.47 Created by Seminario method using MCPB.py
ca-Z2-M2 54.33 111.17 Created by Seminario method using MCPB.py
ca-Z3-M2 53.80 112.08 Created by Seminario method using MCPB.py
ca-Z4-M3 39.92 106.51 Created by Seminario method using MCPB.py
ca-Z5-M3 40.58 108.54 Created by Seminario method using MCPB.py
ca-Z6-M3 41.09 110.04 Created by Seminario method using MCPB.py
ca-Z7-M3 51.86 109.08 Created by Seminario method using MCPB.py
ca-Z8-M3 48.76 111.98 Created by Seminario method using MCPB.py
ca-Z9-M3 57.49 110.93 Created by Seminario method using MCPB.py
ca-ca-A1 71.37 123.26 SOURCE4_SOURCE5 35 1.2620

Charges:

```
 1 C1    3.8930   3.0180   0.2580 ca    1 BOX   -0.118210
 2 H1    4.4740   2.6430   1.0920 ha    1 BOX    0.097843
 3 C2    2.3130   3.8290  -1.9420 ca    1 BOX    0.014595
 4 C3    3.7300   2.2060  -0.8420 ca    1 BOX   -0.204358
 5 H2    4.1880   1.2260  -0.8590 ha    1 BOX    0.157942
 6 C4    2.9470   2.5980  -1.9450 ca    1 BOX   -0.138191
 7 H3    2.8030   1.9140  -2.7660 ha    1 BOX    0.146863
 8 C5    3.3310   4.3250   0.2700 ca    1 BOX    0.051206
 9 C6    2.5510   4.7550  -0.8610 ca    1 BOX    0.051206
10 C7    3.5440   5.2520   1.3540 ca    1 BOX    0.014595
```

| | | | | | | | |
|---|---|---|---|---|---|---|---|
| 11 | C8 | 2.0710 | 6.0910 | -0.9060 | ca | 1 BOX | -0.118210 |
| 12 | H4 | 1.5190 | 6.4550 | -1.7640 | ha | 1 BOX | 0.097843 |
| 13 | C9 | 3.0470 | 6.5440 | 1.2680 | ca | 1 BOX | -0.138191 |
| 14 | H5 | 3.2280 | 7.2430 | 2.0710 | ha | 1 BOX | 0.146863 |
| 15 | C10 | 2.3370 | 6.9590 | 0.1260 | ca | 1 BOX | -0.204358 |
| 16 | H6 | 1.9840 | 7.9830 | 0.0700 | ha | 1 BOX | 0.157942 |
| 17 | C11 | 4.3760 | 5.3340 | 3.7190 | c | 1 BOX | 0.423034 |
| 18 | C12 | 5.3670 | 4.6870 | 4.6270 | ca | 1 BOX | -0.071397 |
| 19 | C13 | 6.0570 | 3.4930 | 4.3140 | ca | 1 BOX | 0.064252 |
| 20 | C14 | 5.6700 | 5.3560 | 5.8390 | ca | 1 BOX | -0.200037 |
| 21 | H7 | 5.1230 | 6.2620 | 6.0790 | ha | 1 BOX | 0.123842 |
| 22 | C15 | 7.1210 | 3.0430 | 5.1630 | ca | 1 BOX | 0.222567 |
| 23 | C16 | 7.3960 | 3.7310 | 6.3420 | ca | 1 BOX | -0.191262 |
| 24 | H8 | 8.1960 | 3.3700 | 6.9840 | ha | 1 BOX | 0.119344 |
| 25 | C17 | 6.6570 | 4.8800 | 6.6850 | ca | 1 BOX | -0.254700 |
| 26 | H9 | 6.8820 | 5.4040 | 7.6110 | ha | 1 BOX | 0.154515 |
| 27 | C18 | 1.1600 | 3.6940 | -4.1700 | c | 1 BOX | 0.423034 |
| 28 | C19 | 0.1520 | 4.4240 | -4.9920 | ca | 1 BOX | -0.071397 |
| 29 | C20 | 0.0170 | 4.0370 | -6.3480 | ca | 1 BOX | -0.200037 |
| 30 | H10 | 0.6220 | 3.2120 | -6.7090 | ha | 1 BOX | 0.123842 |
| 31 | C21 | -0.6330 | 5.4970 | -4.5060 | ca | 1 BOX | 0.064252 |
| 32 | C22 | -0.8550 | 4.7000 | -7.1950 | ca | 1 BOX | -0.254700 |
| 33 | H11 | -0.9440 | 4.3940 | -8.2340 | ha | 1 BOX | 0.154515 |
| 34 | C23 | -1.5340 | 6.1810 | -5.3910 | ca | 1 BOX | 0.222567 |
| 35 | C24 | -1.6320 | 5.7750 | -6.7180 | ca | 1 BOX | -0.191262 |
| 36 | H12 | -2.3220 | 6.2990 | -7.3770 | ha | 1 BOX | 0.119344 |
| 37 | C25 | 3.4170 | -5.1370 | -1.8140 | ca | 1 BOX | 0.014595 |
| 38 | C26 | 5.7400 | -3.5740 | -1.4760 | ca | 1 BOX | -0.118210 |
| 39 | H13 | 6.6340 | -2.9780 | -1.3420 | ha | 1 BOX | 0.097843 |
| 40 | C27 | 5.8220 | -4.8520 | -1.9810 | ca | 1 BOX | -0.204358 |
| 41 | H14 | 6.7870 | -5.2660 | -2.2610 | ha | 1 BOX | 0.157942 |
| 42 | C28 | 4.6590 | -5.6270 | -2.1740 | ca | 1 BOX | -0.138191 |
| 43 | H15 | 4.7330 | -6.6090 | -2.6270 | ha | 1 BOX | 0.146863 |
| 44 | C29 | 4.4830 | -3.0380 | -1.0880 | ca | 1 BOX | 0.051206 |
| 45 | C30 | 3.3000 | -3.8460 | -1.2040 | ca | 1 BOX | 0.051206 |
| 46 | C31 | 4.3610 | -1.7270 | -0.5340 | ca | 1 BOX | 0.014595 |
| 47 | C32 | 2.0710 | -3.3600 | -0.6800 | ca | 1 BOX | -0.118210 |
| 48 | H16 | 1.1780 | -3.9720 | -0.7520 | ha | 1 BOX | 0.097843 |
| 49 | C33 | 3.1630 | -1.3110 | 0.0160 | ca | 1 BOX | -0.138191 |
| 50 | H17 | 3.0980 | -0.3360 | 0.4890 | ha | 1 BOX | 0.146863 |
| 51 | C34 | 2.0230 | -2.1390 | -0.0400 | ca | 1 BOX | -0.204358 |
| 52 | H18 | 1.1030 | -1.7870 | 0.4100 | ha | 1 BOX | 0.157942 |
| 53 | C35 | 6.1990 | -0.3350 | -1.4740 | c | 1 BOX | 0.423034 |
| 54 | C36 | 7.1980 | 0.7060 | -1.0980 | ca | 1 BOX | -0.071397 |
| 55 | C37 | 7.2740 | 1.2580 | 0.2000 | ca | 1 BOX | 0.064252 |
| 56 | C38 | 8.3270 | 2.1730 | 0.5440 | ca | 1 BOX | 0.222567 |
| 57 | C39 | 9.2170 | 2.5730 | -0.4520 | ca | 1 BOX | -0.191262 |
| 58 | H19 | 10.0080 | 3.2750 | -0.1960 | ha | 1 BOX | 0.119344 |

| | | | | | | | |
|---|---|---|---|---|---|---|---|
| 59 | C40 | 9.1010 | 2.0680 | -1.7650 | ca | 1 BOX | -0.254700 |
| 60 | H20 | 9.8030 | 2.3980 | -2.5270 | ha | 1 BOX | 0.154515 |
| 61 | C41 | 8.1170 | 1.1450 | -2.0820 | ca | 1 BOX | -0.200037 |
| 62 | H21 | 8.0410 | 0.7340 | -3.0840 | ha | 1 BOX | 0.123842 |
| 63 | C42 | 2.1280 | -7.2340 | -2.0180 | c | 1 BOX | 0.423034 |
| 64 | C43 | 1.0720 | -7.8760 | -2.8450 | ca | 1 BOX | -0.071397 |
| 65 | C44 | 0.9870 | -9.2920 | -2.8130 | ca | 1 BOX | -0.200037 |
| 66 | H22 | 1.5870 | -9.8290 | -2.0860 | ha | 1 BOX | 0.123842 |
| 67 | C45 | 0.1840 | -9.9740 | -3.7100 | ca | 1 BOX | -0.254700 |
| 68 | H23 | 0.1210 | -11.0590 | -3.6720 | ha | 1 BOX | 0.154515 |
| 69 | C46 | -0.5270 | -9.2710 | -4.7050 | ca | 1 BOX | -0.191262 |
| 70 | H24 | -1.1160 | -9.8040 | -5.4480 | ha | 1 BOX | 0.119344 |
| 71 | C47 | -0.4700 | -7.8810 | -4.7690 | ca | 1 BOX | 0.222567 |
| 72 | C48 | 0.2710 | -7.1610 | -3.7680 | ca | 1 BOX | 0.064252 |
| 73 | C49 | -0.2680 | 1.6540 | -1.6690 | ca | 1 BOX | -0.204358 |
| 74 | H25 | 0.1720 | 2.3030 | -0.9230 | ha | 1 BOX | 0.157942 |
| 75 | C50 | 0.4430 | 0.5060 | -2.0730 | ca | 1 BOX | -0.138191 |
| 76 | H26 | 1.4030 | 0.2880 | -1.6310 | ha | 1 BOX | 0.146863 |
| 77 | C51 | -1.4910 | 1.9650 | -2.2170 | ca | 1 BOX | -0.118210 |
| 78 | H27 | -2.0100 | 2.8610 | -1.8960 | ha | 1 BOX | 0.097843 |
| 79 | C52 | -0.0730 | -0.3370 | -3.0410 | ca | 1 BOX | 0.014595 |
| 80 | C53 | -2.0900 | 1.0880 | -3.1630 | ca | 1 BOX | 0.051206 |
| 81 | C54 | -1.3950 | -0.1040 | -3.5690 | ca | 1 BOX | 0.051206 |
| 82 | C55 | -3.3850 | 1.3570 | -3.7220 | ca | 1 BOX | 0.014595 |
| 83 | C56 | -2.0450 | -1.0120 | -4.4530 | ca | 1 BOX | -0.118210 |
| 84 | H28 | -1.5550 | -1.9210 | -4.7790 | ha | 1 BOX | 0.097843 |
| 85 | C57 | -3.9940 | 0.4320 | -4.5520 | ca | 1 BOX | -0.138191 |
| 86 | H29 | -4.9780 | 0.6380 | -4.9540 | ha | 1 BOX | 0.146863 |
| 87 | C58 | -3.3240 | -0.7580 | -4.9010 | ca | 1 BOX | -0.204358 |
| 88 | H30 | -3.8130 | -1.4690 | -5.5600 | ha | 1 BOX | 0.157942 |
| 89 | C59 | -5.2980 | 2.8640 | -3.2160 | c | 1 BOX | 0.423034 |
| 90 | C60 | -5.6550 | 4.2950 | -3.0180 | ca | 1 BOX | -0.071397 |
| 91 | C61 | -7.0300 | 4.6000 | -2.8500 | ca | 1 BOX | -0.200037 |
| 92 | H31 | -7.7450 | 3.7850 | -2.8840 | ha | 1 BOX | 0.123842 |
| 93 | C62 | -7.4510 | 5.9020 | -2.6470 | ca | 1 BOX | -0.254700 |
| 94 | H32 | -8.5080 | 6.1220 | -2.5200 | ha | 1 BOX | 0.154515 |
| 95 | C63 | -6.5110 | 6.9510 | -2.6070 | ca | 1 BOX | -0.191262 |
| 96 | H33 | -6.8300 | 7.9790 | -2.4520 | ha | 1 BOX | 0.119344 |
| 97 | C64 | -5.1520 | 6.6940 | -2.7670 | ca | 1 BOX | 0.222567 |
| 98 | C65 | -4.7020 | 5.3420 | -2.9660 | ca | 1 BOX | 0.064252 |
| 99 | C66 | 2.0190 | -1.4860 | -3.7480 | c | 1 BOX | 0.423034 |
| 100 | C67 | 2.4980 | -2.6570 | -4.5380 | ca | 1 BOX | -0.071397 |
| 101 | C68 | 1.6310 | -3.5540 | -5.2010 | ca | 1 BOX | 0.064252 |
| 102 | C69 | 3.8980 | -2.8610 | -4.6290 | ca | 1 BOX | -0.200037 |
| 103 | H34 | 4.5550 | -2.1690 | -4.1160 | ha | 1 BOX | 0.123842 |
| 104 | C70 | 4.4110 | -3.9400 | -5.3270 | ca | 1 BOX | -0.254700 |
| 105 | H35 | 5.4860 | -4.0960 | -5.3710 | ha | 1 BOX | 0.154515 |
| 106 | C71 | 2.1680 | -4.6820 | -5.9100 | ca | 1 BOX | 0.222567 |

| | | | | | | | | |
|---|---|---|---|---|---|---|---|---|
| 107 | C72 | 3.5490 | -4.8590 | -5.9600 | ca | 1 | BOX | -0.191262 |
| 108 | H36 | 3.9470 | -5.7190 | -6.4930 | ha | 1 | BOX | 0.119344 |
| 109 | C73 | -1.5300 | -2.4660 | -0.4230 | ca | 1 | BOX | -0.204358 |
| 110 | H37 | -0.7970 | -1.7590 | -0.7930 | ha | 1 | BOX | 0.157942 |
| 111 | C74 | -1.5700 | -2.7800 | 0.9540 | ca | 1 | BOX | -0.138191 |
| 112 | H38 | -0.8490 | -2.3430 | 1.6390 | ha | 1 | BOX | 0.146863 |
| 113 | C75 | -2.4240 | -3.0450 | -1.2950 | ca | 1 | BOX | -0.118210 |
| 114 | H39 | -2.3820 | -2.7890 | -2.3480 | ha | 1 | BOX | 0.097843 |
| 115 | C76 | -2.5340 | -3.6340 | 1.4510 | ca | 1 | BOX | 0.014595 |
| 116 | C77 | -3.4320 | -3.9280 | -0.8190 | ca | 1 | BOX | 0.051206 |
| 117 | C78 | -3.5220 | -4.2010 | 0.5870 | ca | 1 | BOX | 0.051206 |
| 118 | C79 | -4.4010 | -4.5200 | -1.6960 | ca | 1 | BOX | 0.014595 |
| 119 | C80 | -4.6060 | -4.9750 | 1.0790 | ca | 1 | BOX | -0.118210 |
| 120 | H40 | -4.7040 | -5.1360 | 2.1460 | ha | 1 | BOX | 0.097843 |
| 121 | C81 | -5.4510 | -5.2580 | -1.1810 | ca | 1 | BOX | -0.138191 |
| 122 | H41 | -6.1810 | -5.6910 | -1.8550 | ha | 1 | BOX | 0.146863 |
| 123 | C82 | -5.5560 | -5.4680 | 0.2110 | ca | 1 | BOX | -0.204358 |
| 124 | H42 | -6.3980 | -6.0350 | 0.5980 | ha | 1 | BOX | 0.157942 |
| 125 | C83 | -5.1070 | -4.1980 | -4.0690 | c | 1 | BOX | 0.423034 |
| 126 | C84 | -4.5450 | -4.1500 | -5.4480 | ca | 1 | BOX | -0.071397 |
| 127 | C85 | -3.1800 | -4.3930 | -5.7370 | ca | 1 | BOX | 0.064252 |
| 128 | C86 | -5.4330 | -3.8610 | -6.5130 | ca | 1 | BOX | -0.200037 |
| 129 | H43 | -6.4730 | -3.6630 | -6.2780 | ha | 1 | BOX | 0.123842 |
| 130 | C87 | -4.9830 | -3.8320 | -7.8220 | ca | 1 | BOX | -0.254700 |
| 131 | H44 | -5.6710 | -3.6000 | -8.6310 | ha | 1 | BOX | 0.154515 |
| 132 | C88 | -3.6340 | -4.1180 | -8.1190 | ca | 1 | BOX | -0.191262 |
| 133 | H45 | -3.2800 | -4.1180 | -9.1470 | ha | 1 | BOX | 0.119344 |
| 134 | C89 | -2.7320 | -4.4140 | -7.1000 | ca | 1 | BOX | 0.222567 |
| 135 | C90 | -2.3630 | -5.0150 | 3.5050 | c | 1 | BOX | 0.423034 |
| 136 | C91 | -2.4660 | -4.9600 | 4.9930 | ca | 1 | BOX | -0.071397 |
| 137 | C92 | -2.2120 | -6.1350 | 5.7430 | ca | 1 | BOX | -0.200037 |
| 138 | H46 | -1.9000 | -7.0310 | 5.2170 | ha | 1 | BOX | 0.123842 |
| 139 | C93 | -2.8500 | -3.7890 | 5.6820 | ca | 1 | BOX | 0.064252 |
| 140 | C94 | -2.3670 | -6.1390 | 7.1200 | ca | 1 | BOX | -0.254700 |
| 141 | H47 | -2.1630 | -7.0440 | 7.6870 | ha | 1 | BOX | 0.154515 |
| 142 | C95 | -2.8130 | -4.9840 | 7.8010 | ca | 1 | BOX | -0.191262 |
| 143 | H48 | -2.9680 | -5.0030 | 8.8770 | ha | 1 | BOX | 0.119344 |
| 144 | C96 | -3.0770 | -3.8100 | 7.1000 | ca | 1 | BOX | 0.222567 |
| 145 | C97 | -0.5890 | 2.5330 | 1.5940 | ca | 1 | BOX | -0.138191 |
| 146 | H49 | 0.4240 | 2.9050 | 1.6950 | ha | 1 | BOX | 0.146863 |
| 147 | C98 | -0.8790 | 1.1770 | 1.8630 | ca | 1 | BOX | -0.204358 |
| 148 | H50 | -0.0710 | 0.5130 | 2.1410 | ha | 1 | BOX | 0.157942 |
| 149 | C99 | -1.5820 | 3.3740 | 1.1390 | ca | 1 | BOX | 0.014595 |
| 150 | C100 | -2.1650 | 0.6960 | 1.7760 | ca | 1 | BOX | -0.118210 |
| 151 | H51 | -2.3710 | -0.3430 | 2.0090 | ha | 1 | BOX | 0.097843 |
| 152 | C101 | -2.9190 | 2.8880 | 0.9380 | ca | 1 | BOX | 0.051206 |
| 153 | C102 | -3.2180 | 1.5380 | 1.3230 | ca | 1 | BOX | 0.051206 |
| 154 | C103 | -3.9420 | 3.6870 | 0.3590 | ca | 1 | BOX | -0.118210 |

| | | | | | | | |
|---|---|---|---|---|---|---|---|
| 155 | H52 | -3.7340 | 4.7030 | 0.0470 | ha | 1 BOX | 0.097843 |
| 156 | C104 | -4.5700 | 1.0740 | 1.2120 | ca | 1 BOX | 0.014595 |
| 157 | C105 | -5.2050 | 3.1670 | 0.1710 | ca | 1 BOX | -0.204358 |
| 158 | H53 | -5.9760 | 3.7760 | -0.2910 | ha | 1 BOX | 0.157942 |
| 159 | C106 | -5.5310 | 1.8680 | 0.6080 | ca | 1 BOX | -0.138191 |
| 160 | H54 | -6.5450 | 1.5030 | 0.5050 | ha | 1 BOX | 0.146863 |
| 161 | C107 | -0.7900 | 5.7000 | 1.5690 | c | 1 BOX | 0.423034 |
| 162 | C108 | -0.8490 | 7.0690 | 0.9740 | ca | 1 BOX | -0.071397 |
| 163 | C109 | -1.4030 | 7.3180 | -0.3020 | ca | 1 BOX | 0.064252 |
| 164 | C110 | -0.2280 | 8.1360 | 1.6630 | ca | 1 BOX | -0.200037 |
| 165 | H55 | 0.1800 | 7.9470 | 2.6510 | ha | 1 BOX | 0.123842 |
| 166 | C111 | -0.0960 | 9.3800 | 1.0650 | ca | 1 BOX | -0.254700 |
| 167 | H56 | 0.3810 | 10.1950 | 1.6040 | ha | 1 BOX | 0.154515 |
| 168 | C112 | -1.1620 | 8.5640 | -0.9680 | ca | 1 BOX | 0.222567 |
| 169 | C113 | -0.5290 | 9.5870 | -0.2630 | ca | 1 BOX | -0.191262 |
| 170 | H57 | -0.3750 | 10.5460 | -0.7520 | ha | 1 BOX | 0.119344 |
| 171 | C114 | -5.9080 | -1.0050 | 1.4750 | c | 1 BOX | 0.423034 |
| 172 | C115 | -6.3130 | -1.9910 | 2.5110 | ca | 1 BOX | -0.071397 |
| 173 | C116 | -7.1910 | -3.0280 | 2.1090 | ca | 1 BOX | -0.200037 |
| 174 | H58 | -7.5040 | -3.0620 | 1.0710 | ha | 1 BOX | 0.123842 |
| 175 | C117 | -5.8910 | -1.9200 | 3.8620 | ca | 1 BOX | 0.064252 |
| 176 | C118 | -7.6110 | -3.9940 | 3.0050 | ca | 1 BOX | -0.254700 |
| 177 | H59 | -8.2740 | -4.7920 | 2.6800 | ha | 1 BOX | 0.154515 |
| 178 | C119 | -6.3190 | -2.9410 | 4.7820 | ca | 1 BOX | 0.222567 |
| 179 | C120 | -7.1660 | -3.9560 | 4.3420 | ca | 1 BOX | -0.191262 |
| 180 | H60 | -7.4780 | -4.7160 | 5.0550 | ha | 1 BOX | 0.119344 |
| 181 | C121 | 1.6320 | -2.5640 | 3.8390 | ca | 1 BOX | -0.138191 |
| 182 | H61 | 1.9770 | -3.5650 | 3.6270 | ha | 1 BOX | 0.146863 |
| 183 | C122 | 0.3040 | -2.3490 | 4.2570 | ca | 1 BOX | -0.204358 |
| 184 | H62 | -0.3660 | -3.1970 | 4.3330 | ha | 1 BOX | 0.157942 |
| 185 | C123 | 2.5040 | -1.4930 | 3.7140 | ca | 1 BOX | 0.014595 |
| 186 | C124 | -0.1490 | -1.0910 | 4.5890 | ca | 1 BOX | -0.118210 |
| 187 | H63 | -1.1840 | -0.9820 | 4.8870 | ha | 1 BOX | 0.097843 |
| 188 | C125 | 2.0420 | -0.1510 | 3.9740 | ca | 1 BOX | 0.051206 |
| 189 | C126 | 0.7120 | 0.0350 | 4.4840 | ca | 1 BOX | 0.051206 |
| 190 | C127 | 2.8510 | 0.9890 | 3.7080 | ca | 1 BOX | -0.118210 |
| 191 | H64 | 3.8330 | 0.8870 | 3.2610 | ha | 1 BOX | 0.097843 |
| 192 | C128 | 0.2720 | 1.3700 | 4.8030 | ca | 1 BOX | 0.014595 |
| 193 | C129 | 2.3770 | 2.2510 | 3.9880 | ca | 1 BOX | -0.204358 |
| 194 | H65 | 2.9920 | 3.1120 | 3.7620 | ha | 1 BOX | 0.157942 |
| 195 | C130 | 1.1060 | 2.4510 | 4.5600 | ca | 1 BOX | -0.138191 |
| 196 | H66 | 0.7680 | 3.4530 | 4.7760 | ha | 1 BOX | 0.146863 |
| 197 | C131 | -1.8470 | 2.5980 | 5.3410 | c | 1 BOX | 0.423034 |
| 198 | C132 | -3.1790 | 2.3900 | 5.9790 | ca | 1 BOX | -0.071397 |
| 199 | C133 | -3.6840 | 1.1230 | 6.3480 | ca | 1 BOX | 0.064252 |
| 200 | C134 | -4.9840 | 1.0140 | 6.9460 | ca | 1 BOX | 0.222567 |
| 201 | C135 | -5.7310 | 2.1660 | 7.1750 | ca | 1 BOX | -0.191262 |
| 202 | H67 | -6.7130 | 2.0720 | 7.6320 | ha | 1 BOX | 0.119344 |

| | | | | | | | | |
|---|---|---|---|---|---|---|---|---|
| 203 | C136 | -5.2210 | 3.4290 | 6.8120 | ca | 1 | BOX | -0.254700 |
| 204 | H68 | -5.8170 | 4.3190 | 6.9960 | ha | 1 | BOX | 0.154515 |
| 205 | C137 | -3.9740 | 3.5380 | 6.2190 | ca | 1 | BOX | -0.200037 |
| 206 | H69 | -3.5810 | 4.5070 | 5.9310 | ha | 1 | BOX | 0.123842 |
| 207 | C138 | 4.4880 | -2.7010 | 2.7840 | c | 1 | BOX | 0.423034 |
| 208 | C139 | 5.9510 | -2.5280 | 2.5800 | ca | 1 | BOX | -0.071397 |
| 209 | C140 | 6.6670 | -1.3590 | 2.9350 | ca | 1 | BOX | 0.064252 |
| 210 | C141 | 8.0490 | -1.2430 | 2.5610 | ca | 1 | BOX | 0.222567 |
| 211 | C142 | 8.6830 | -2.2980 | 1.9100 | ca | 1 | BOX | -0.191262 |
| 212 | H70 | 9.7290 | -2.1870 | 1.6320 | ha | 1 | BOX | 0.119344 |
| 213 | C143 | 7.9740 | -3.4750 | 1.5980 | ca | 1 | BOX | -0.254700 |
| 214 | H71 | 8.4790 | -4.2890 | 1.0860 | ha | 1 | BOX | 0.154515 |
| 215 | C144 | 6.6320 | -3.5820 | 1.9200 | ca | 1 | BOX | -0.200037 |
| 216 | H72 | 6.0690 | -4.4690 | 1.6500 | ha | 1 | BOX | 0.123842 |
| 217 | N1 | 1.4180 | 4.2450 | -2.9410 | n | 1 | BOX | -0.205011 |
| 218 | H73 | 0.7560 | 4.9860 | -2.6950 | hn | 1 | BOX | 0.188275 |
| 219 | N2 | 4.3200 | 4.8210 | 2.4450 | n | 1 | BOX | -0.205011 |
| 220 | H74 | 4.8710 | 3.9670 | 2.3360 | hn | 1 | BOX | 0.188275 |
| 221 | N3 | 2.2370 | -5.8690 | -2.1040 | n | 1 | BOX | -0.205011 |
| 222 | H75 | 1.5330 | -5.4150 | -2.6960 | hn | 1 | BOX | 0.188275 |
| 223 | N4 | 5.4810 | -0.8660 | -0.4360 | n | 1 | BOX | -0.205011 |
| 224 | H76 | 5.5990 | -0.3600 | 0.4500 | hn | 1 | BOX | 0.188275 |
| 225 | N5 | -4.8940 | -0.1540 | 1.8290 | n | 1 | BOX | -0.205011 |
| 226 | H77 | -4.5550 | -0.2820 | 2.7880 | hn | 1 | BOX | 0.188275 |
| 227 | N6 | -1.3170 | 4.7130 | 0.7820 | n | 1 | BOX | -0.205011 |
| 228 | H78 | -1.7540 | 5.0610 | -0.0780 | hn | 1 | BOX | 0.188275 |
| 229 | N7 | -2.6010 | -3.8410 | 2.8530 | n | 1 | BOX | -0.205011 |
| 230 | H79 | -2.7710 | -3.0310 | 3.4580 | hn | 1 | BOX | 0.188275 |
| 231 | N8 | -4.1850 | -4.3700 | -3.0770 | n | 1 | BOX | -0.205011 |
| 232 | H80 | -3.2170 | -4.4320 | -3.4110 | hn | 1 | BOX | 0.188275 |
| 233 | N9 | 0.6620 | -1.4300 | -3.5350 | n | 1 | BOX | -0.205011 |
| 234 | H81 | 0.1550 | -2.1840 | -4.0030 | hn | 1 | BOX | 0.188275 |
| 235 | N10 | -1.0110 | 1.5090 | 5.3550 | n | 1 | BOX | -0.205011 |
| 236 | H82 | -1.4680 | 0.6640 | 5.7030 | hn | 1 | BOX | 0.188275 |
| 237 | N11 | -3.9740 | 2.6070 | -3.4470 | n | 1 | BOX | -0.205011 |
| 238 | H83 | -3.3650 | 3.4270 | -3.3650 | hn | 1 | BOX | 0.188275 |
| 239 | N12 | 3.8580 | -1.6450 | 3.3870 | n | 1 | BOX | -0.205011 |
| 240 | H84 | 4.4950 | -0.8730 | 3.6090 | hn | 1 | BOX | 0.188275 |
| 241 | O1 | 1.2880 | -5.5260 | -6.4790 | Y1 | 1 | BOX | -0.594030 |
| 242 | O2 | 0.3000 | -3.4420 | -5.2040 | Y2 | 1 | BOX | -0.406087 |
| 243 | O3 | -2.2530 | -4.6310 | -4.8090 | Y3 | 1 | BOX | -0.406087 |
| 244 | O4 | -1.4410 | -4.7230 | -7.3240 | Y4 | 1 | BOX | -0.594030 |
| 245 | O5 | -1.0670 | -7.1610 | -5.7340 | Y5 | 1 | BOX | -0.594030 |
| 246 | O6 | 0.1920 | -5.8350 | -3.8120 | Y6 | 1 | BOX | -0.406087 |
| 247 | O7 | -0.6210 | 5.9270 | -3.2440 | Y7 | 1 | BOX | -0.406087 |
| 248 | O8 | -2.2570 | 7.1910 | -4.8650 | Y8 | 1 | BOX | -0.594030 |
| 249 | O9 | -3.3900 | 5.1640 | -3.0940 | Y9 | 1 | BOX | -0.406087 |
| 250 | O10 | -4.2220 | 7.6640 | -2.7360 | Z1 | 1 | BOX | -0.594030 |

| | | | | | | | |
|---|---|---|---|---|---|---|---|
| 251 O11 | -1.5740 | 8.6700 | -2.2480 Z2 | 1 BOX | -0.594030 |
| 252 O12 | -2.1350 | 6.4310 | -0.9720 Z3 | 1 BOX | -0.406087 |
| 253 O13 | 6.1400 | -0.2970 | 3.5600 Z4 | 1 BOX | -0.406087 |
| 254 O14 | 8.6560 | -0.0760 | 2.8330 Z5 | 1 BOX | -0.594030 |
| 255 O15 | 6.4230 | 0.9550 | 1.1750 Z6 | 1 BOX | -0.406087 |
| 256 O16 | 8.3950 | 2.5780 | 1.8240 Z7 | 1 BOX | -0.594030 |
| 257 O17 | 7.8170 | 1.9630 | 4.7590 Z8 | 1 BOX | -0.594030 |
| 258 O18 | 5.8060 | 2.7470 | 3.2380 Z9 | 1 BOX | -0.406087 |
| 259 O19 | -5.8760 | -2.8610 | 6.0460 A1 | 1 BOX | -0.594030 |
| 260 O20 | -5.1110 | -0.9610 | 4.3590 A2 | 1 BOX | -0.406087 |
| 261 O21 | -3.0730 | -2.6240 | 5.0850 A3 | 1 BOX | -0.406087 |
| 262 O22 | -3.5530 | -2.6780 | 7.6570 A4 | 1 BOX | -0.594030 |
| 263 O23 | -3.0360 | -0.0290 | 6.1610 A5 | 1 BOX | -0.406087 |
| 264 O24 | -5.4160 | -0.2270 | 7.2440 A6 | 1 BOX | -0.594030 |
| 265 O25 | -6.4410 | -0.9680 | 0.3480 o | 1 BOX | -0.610825 |
| 266 O26 | -6.3290 | -4.0870 | -3.8410 o | 1 BOX | -0.610825 |
| 267 O27 | -6.1560 | 1.9580 | -3.1790 o | 1 BOX | -0.610825 |
| 268 O28 | 1.7580 | 2.6910 | -4.6070 o | 1 BOX | -0.610825 |
| 269 O29 | 2.8090 | -0.6200 | -3.3260 o | 1 BOX | -0.610825 |
| 270 O30 | 6.0570 | -0.7120 | -2.6540 o | 1 BOX | -0.610825 |
| 271 O31 | 3.6860 | 6.3030 | 4.0900 o | 1 BOX | -0.610825 |
| 272 O32 | 3.8870 | -3.7280 | 2.4100 o | 1 BOX | -0.610825 |
| 273 O33 | -2.0900 | -6.0690 | 2.8920 o | 1 BOX | -0.610825 |
| 274 O34 | -0.2710 | 5.4760 | 2.6810 o | 1 BOX | -0.610825 |
| 275 O35 | 2.9150 | -7.9070 | -1.3200 o | 1 BOX | -0.610825 |
| 276 O36 | -1.5200 | 3.6980 | 4.8580 o | 1 BOX | -0.610825 |
| 277 GA1 | -0.5120 | -5.2420 | -5.6460 M1 | 1 BOX | 0.689487 |
| 278 GA2 | -2.3770 | 6.9510 | -2.8940 M2 | 1 BOX | 0.689487 |
| 279 GA3 | 7.2580 | 1.3520 | 2.9680 M3 | 1 BOX | 0.689487 |
| 280 GA4 | -4.3730 | -1.5550 | 6.2010 M4 | 1 BOX | 0.689487 |

Force field parameters and charges derived in gas phase

BOND

| | | | |
|---|---|---|---|
| A1-M4 | 45.0 | 2.0010 | Created by Seminario method using MCPB.py |
| A2-M4 | 15.0 | 2.0988 | Created by Seminario method using MCPB.py |
| A3-M4 | 14.2 | 2.0806 | Created by Seminario method using MCPB.py |
| A4-M4 | 46.6 | 1.9992 | Created by Seminario method using MCPB.py |
| A5-M4 | 5.6 | 2.1491 | Created by Seminario method using MCPB.py |
| A6-M4 | 44.8 | 2.0010 | Created by Seminario method using MCPB.py |
| Y1-M1 | 48.3 | 1.9980 | Created by Seminario method using MCPB.py |
| Y2-M1 | 15.5 | 2.0804 | Created by Seminario method using MCPB.py |
| Y3-M1 | 8.0 | 2.1338 | Created by Seminario method using MCPB.py |
| Y4-M1 | 52.3 | 1.9895 | Created by Seminario method using MCPB.py |
| Y5-M1 | 48.9 | 1.9969 | Created by Seminario method using MCPB.py |
| Y6-M1 | 4.5 | 2.1585 | Created by Seminario method using MCPB.py |
| Y7-M2 | 6.3 | 2.1486 | Created by Seminario method using MCPB.py |
| Y8-M2 | 44.0 | 2.0037 | Created by Seminario method using MCPB.py |

Y9-M2    8.9    2.1328      Created by Seminario method using MCPB.py
Z1-M2   48.7    1.9918      Created by Seminario method using MCPB.py
Z2-M2   45.8    1.9987      Created by Seminario method using MCPB.py
Z3-M2   21.8    2.0441      Created by Seminario method using MCPB.py
Z4-M3   12.1    2.1109      Created by Seminario method using MCPB.py
Z5-M3   43.4    2.0057      Created by Seminario method using MCPB.py
Z6-M3   10.8    2.1242      Created by Seminario method using MCPB.py
Z7-M3   46.8    1.9995      Created by Seminario method using MCPB.py
Z8-M3   47.2    2.0009      Created by Seminario method using MCPB.py
Z9-M3   12.0    2.1180      Created by Seminario method using MCPB.py
ca-A1  598.1    1.2358         SOURCE4_SOURCE5      17  0.0088

ANGL
A1-M4-A2   116.15      80.77    Created by Seminario method using MCPB.py
A1-M4-A3    54.66      92.38    Created by Seminario method using MCPB.py
A1-M4-A4    70.38      93.10    Created by Seminario method using MCPB.py
A1-M4-A5    89.64     168.75    Created by Seminario method using MCPB.py
A1-M4-A6    88.89      89.31    Created by Seminario method using MCPB.py
A2-M4-A3    61.57      96.81    Created by Seminario method using MCPB.py
A2-M4-A4    94.45     173.51    Created by Seminario method using MCPB.py
A2-M4-A5    64.27      96.62    Created by Seminario method using MCPB.py
A2-M4-A6    51.98      90.64    Created by Seminario method using MCPB.py
A3-M4-A4   121.35      81.23    Created by Seminario method using MCPB.py
A3-M4-A5    54.76      98.80    Created by Seminario method using MCPB.py
A3-M4-A6    94.73     172.53    Created by Seminario method using MCPB.py
A4-M4-A5    54.79      89.81    Created by Seminario method using MCPB.py
A4-M4-A6    79.77      91.42    Created by Seminario method using MCPB.py
A5-M4-A6    93.71      79.75    Created by Seminario method using MCPB.py
Y1-M1-Y2   121.57      81.57    Created by Seminario method using MCPB.py
Y1-M1-Y3    55.98     172.02    Created by Seminario method using MCPB.py
Y1-M1-Y4    72.22      91.77    Created by Seminario method using MCPB.py
Y1-M1-Y5    92.58      90.83    Created by Seminario method using MCPB.py
Y1-M1-Y6    59.44      87.47    Created by Seminario method using MCPB.py
Y2-M1-Y3    74.43      99.37    Created by Seminario method using MCPB.py
Y2-M1-Y4    54.16      91.28    Created by Seminario method using MCPB.py
Y2-M1-Y5   108.94     171.92    Created by Seminario method using MCPB.py
Y2-M1-Y6    51.72      97.32    Created by Seminario method using MCPB.py
Y3-M1-Y4    98.87      80.29    Created by Seminario method using MCPB.py
Y3-M1-Y5    65.73      88.55    Created by Seminario method using MCPB.py
Y3-M1-Y6    63.72     100.23    Created by Seminario method using MCPB.py
Y4-M1-Y5    84.37      91.69    Created by Seminario method using MCPB.py
Y4-M1-Y6    65.36     171.16    Created by Seminario method using MCPB.py
Y5-M1-Y6    90.80      79.51    Created by Seminario method using MCPB.py
Y7-M2-Y8    95.16      79.46    Created by Seminario method using MCPB.py
Y7-M2-Y9    63.48     100.54    Created by Seminario method using MCPB.py
Y7-M2-Z1    69.27     170.18    Created by Seminario method using MCPB.py
Y7-M2-Z2    42.20      86.63    Created by Seminario method using MCPB.py
Y7-M2-Z3    66.25      92.87    Created by Seminario method using MCPB.py

| | | | |
|---|---|---|---|
| Y8-M2-Y9 | 64.08 | 90.03 | Created by Seminario method using MCPB.py |
| Y8-M2-Z1 | 86.64 | 90.75 | Created by Seminario method using MCPB.py |
| Y8-M2-Z2 | 93.78 | 91.84 | Created by Seminario method using MCPB.py |
| Y8-M2-Z3 | 117.75 | 170.78 | Created by Seminario method using MCPB.py |
| Y9-M2-Z1 | 100.54 | 80.09 | Created by Seminario method using MCPB.py |
| Y9-M2-Z2 | 56.04 | 172.81 | Created by Seminario method using MCPB.py |
| Y9-M2-Z3 | 76.68 | 96.46 | Created by Seminario method using MCPB.py |
| Z1-M2-Z2 | 68.34 | 92.94 | Created by Seminario method using MCPB.py |
| Z1-M2-Z3 | 63.23 | 96.80 | Created by Seminario method using MCPB.py |
| Z2-M2-Z3 | 122.74 | 82.53 | Created by Seminario method using MCPB.py |
| Z4-M3-Z5 | 112.58 | 80.39 | Created by Seminario method using MCPB.py |
| Z4-M3-Z6 | 58.53 | 96.35 | Created by Seminario method using MCPB.py |
| Z4-M3-Z7 | 106.35 | 169.34 | Created by Seminario method using MCPB.py |
| Z4-M3-Z8 | 57.07 | 92.68 | Created by Seminario method using MCPB.py |
| Z4-M3-Z9 | 66.05 | 98.87 | Created by Seminario method using MCPB.py |
| Z5-M3-Z6 | 59.82 | 90.71 | Created by Seminario method using MCPB.py |
| Z5-M3-Z7 | 89.53 | 89.59 | Created by Seminario method using MCPB.py |
| Z5-M3-Z8 | 74.00 | 90.36 | Created by Seminario method using MCPB.py |
| Z5-M3-Z9 | 83.15 | 170.32 | Created by Seminario method using MCPB.py |
| Z6-M3-Z7 | 105.38 | 80.02 | Created by Seminario method using MCPB.py |
| Z6-M3-Z8 | 91.88 | 170.96 | Created by Seminario method using MCPB.py |
| Z6-M3-Z9 | 68.21 | 98.96 | Created by Seminario method using MCPB.py |
| Z7-M3-Z8 | 86.91 | 91.00 | Created by Seminario method using MCPB.py |
| Z7-M3-Z9 | 60.35 | 91.62 | Created by Seminario method using MCPB.py |
| Z8-M3-Z9 | 109.04 | 80.02 | Created by Seminario method using MCPB.py |
| ca-A1-M4 | 55.32 | 111.39 | Created by Seminario method using MCPB.py |
| ca-A2-M4 | 48.52 | 108.72 | Created by Seminario method using MCPB.py |
| ca-A3-M4 | 46.98 | 110.68 | Created by Seminario method using MCPB.py |
| ca-A4-M4 | 57.93 | 112.89 | Created by Seminario method using MCPB.py |
| ca-A5-M4 | 59.65 | 109.91 | Created by Seminario method using MCPB.py |
| ca-A6-M4 | 55.83 | 114.22 | Created by Seminario method using MCPB.py |
| ca-Y1-M1 | 58.91 | 112.57 | Created by Seminario method using MCPB.py |
| ca-Y2-M1 | 50.12 | 110.10 | Created by Seminario method using MCPB.py |
| ca-Y3-M1 | 57.90 | 108.64 | Created by Seminario method using MCPB.py |
| ca-Y4-M1 | 55.51 | 112.84 | Created by Seminario method using MCPB.py |
| ca-Y5-M1 | 52.71 | 112.72 | Created by Seminario method using MCPB.py |
| ca-Y6-M1 | 59.39 | 108.02 | Created by Seminario method using MCPB.py |
| ca-Y7-M2 | 54.62 | 108.65 | Created by Seminario method using MCPB.py |
| ca-Y8-M2 | 52.19 | 112.76 | Created by Seminario method using MCPB.py |
| ca-Y9-M2 | 55.63 | 108.73 | Created by Seminario method using MCPB.py |
| ca-Z1-M2 | 54.74 | 112.58 | Created by Seminario method using MCPB.py |
| ca-Z2-M2 | 59.83 | 111.73 | Created by Seminario method using MCPB.py |
| ca-Z3-M2 | 54.80 | 110.70 | Created by Seminario method using MCPB.py |
| ca-Z4-M3 | 49.78 | 110.53 | Created by Seminario method using MCPB.py |
| ca-Z5-M3 | 57.76 | 113.47 | Created by Seminario method using MCPB.py |
| ca-Z6-M3 | 53.64 | 108.88 | Created by Seminario method using MCPB.py |
| ca-Z7-M3 | 55.87 | 112.13 | Created by Seminario method using MCPB.py |
| ca-Z8-M3 | 55.77 | 112.04 | Created by Seminario method using MCPB.py |

ca-Z9-M3 52.48 108.98 Created by Seminario method using MCPB.py
ca-ca-A1 71.37 123.26 SOURCE4_SOURCE5 35 1.2620

Charges:

| | | | | | | | | |
|---|---|---|---|---|---|---|---|---|
| 1 | C1 | 5.3900 | 5.8910 | -1.6280 | ca | 1 | BOX | -0.143496 |
| 2 | H1 | 6.2160 | 5.4540 | -1.0780 | ha | 1 | BOX | 0.139616 |
| 3 | C2 | 3.1970 | 7.0220 | -3.0500 | ca | 1 | BOX | 0.393566 |
| 4 | C3 | 5.5500 | 6.3950 | -2.8970 | ca | 1 | BOX | -0.195908 |
| 5 | H2 | 6.5290 | 6.3550 | -3.3770 | ha | 1 | BOX | 0.135251 |
| 6 | C4 | 4.4690 | 6.9560 | -3.6060 | ca | 1 | BOX | -0.181359 |
| 7 | H3 | 4.6230 | 7.3590 | -4.5970 | ha | 1 | BOX | 0.114661 |
| 8 | C5 | 4.1110 | 5.9140 | -1.0180 | ca | 1 | BOX | 0.001430 |
| 9 | C6 | 2.9840 | 6.4760 | -1.7280 | ca | 1 | BOX | 0.001430 |
| 10 | C7 | 3.9270 | 5.3870 | 0.3040 | ca | 1 | BOX | 0.393566 |
| 11 | C8 | 1.7120 | 6.4750 | -1.0910 | ca | 1 | BOX | -0.143496 |
| 12 | H4 | 0.8500 | 6.9220 | -1.5780 | ha | 1 | BOX | 0.139616 |
| 13 | C9 | 2.6640 | 5.3780 | 0.8580 | ca | 1 | BOX | -0.181359 |
| 14 | H5 | 2.5310 | 4.9760 | 1.8590 | ha | 1 | BOX | 0.114661 |
| 15 | C10 | 1.5560 | 5.9220 | 0.1640 | ca | 1 | BOX | -0.195908 |
| 16 | H6 | 0.5800 | 5.9320 | 0.6420 | ha | 1 | BOX | 0.135251 |
| 17 | C11 | 5.8870 | 5.8030 | 1.7010 | c | 1 | BOX | 0.807075 |
| 18 | C12 | 6.8720 | 5.2730 | 2.6950 | ca | 1 | BOX | -0.153938 |
| 19 | C13 | 7.1600 | 3.9040 | 2.9570 | ca | 1 | BOX | 0.213805 |
| 20 | C14 | 7.5330 | 6.2900 | 3.4490 | ca | 1 | BOX | -0.301874 |
| 21 | H7 | 7.2760 | 7.3170 | 3.2080 | ha | 1 | BOX | 0.116258 |
| 22 | C15 | 8.1360 | 3.5930 | 4.0060 | ca | 1 | BOX | 0.263385 |
| 23 | C16 | 8.7410 | 4.6290 | 4.7210 | ca | 1 | BOX | -0.230920 |
| 24 | H8 | 9.4560 | 4.3520 | 5.4980 | ha | 1 | BOX | 0.042985 |
| 25 | C17 | 8.4410 | 5.9810 | 4.4410 | ca | 1 | BOX | -0.198917 |
| 26 | H9 | 8.9250 | 6.7800 | 5.0140 | ha | 1 | BOX | 0.028481 |
| 27 | C18 | 2.2510 | 8.5880 | -4.7380 | c | 1 | BOX | 0.807075 |
| 28 | C19 | 1.0290 | 8.9840 | -5.4920 | ca | 1 | BOX | -0.153938 |
| 29 | C20 | 1.2530 | 9.9470 | -6.5240 | ca | 1 | BOX | -0.301874 |
| 30 | H10 | 2.2760 | 10.2860 | -6.6630 | ha | 1 | BOX | 0.116258 |
| 31 | C21 | -0.2860 | 8.4890 | -5.2650 | ca | 1 | BOX | 0.213805 |
| 32 | C22 | 0.2200 | 10.4180 | -7.3070 | ca | 1 | BOX | -0.198917 |
| 33 | H11 | 0.4180 | 11.1460 | -8.1010 | ha | 1 | BOX | 0.028481 |
| 34 | C23 | -1.3800 | 9.0300 | -6.0800 | ca | 1 | BOX | 0.263385 |
| 35 | C24 | -1.1000 | 9.9600 | -7.0830 | ca | 1 | BOX | -0.230920 |
| 36 | H12 | -1.9390 | 10.3270 | -7.6770 | ha | 1 | BOX | 0.042985 |
| 37 | C25 | 4.7310 | -6.3050 | -2.4680 | ca | 1 | BOX | 0.393566 |
| 38 | C26 | 6.6540 | -4.8240 | -0.9630 | ca | 1 | BOX | -0.143496 |
| 39 | H13 | 7.3780 | -4.2560 | -0.3930 | ha | 1 | BOX | 0.139616 |
| 40 | C27 | 6.7000 | -6.1950 | -1.0270 | ca | 1 | BOX | -0.195908 |
| 41 | H14 | 7.4800 | -6.7360 | -0.4890 | ha | 1 | BOX | 0.135251 |
| 42 | C28 | 5.7610 | -6.9340 | -1.7740 | ca | 1 | BOX | -0.181359 |
| 43 | H15 | 5.8360 | -8.0080 | -1.8270 | ha | 1 | BOX | 0.114661 |

| | | | | | | | |
|---|---|---|---|---|---|---|---|
| 44 C29 | 5.6210 | -4.1260 | -1.6380 ca | 1 BOX | 0.001430 |
| 45 C30 | 4.6240 | -4.8610 | -2.3850 ca | 1 BOX | 0.001430 |
| 46 C31 | 5.5510 | -2.6920 | -1.5660 ca | 1 BOX | 0.393566 |
| 47 C32 | 3.5680 | -4.1310 | -2.9950 ca | 1 BOX | -0.143496 |
| 48 H16 | 2.8110 | -4.6380 | -3.5890 ha | 1 BOX | 0.139616 |
| 49 C33 | 4.4780 | -2.0410 | -2.1420 ca | 1 BOX | -0.181359 |
| 50 H17 | 4.4200 | -0.9580 | -2.0780 ha | 1 BOX | 0.114661 |
| 51 C34 | 3.4890 | -2.7620 | -2.8490 ca | 1 BOX | -0.195908 |
| 52 H18 | 2.6680 | -2.2210 | -3.3120 ha | 1 BOX | 0.135251 |
| 53 C35 | 7.8480 | -1.8350 | -1.4530 c | 1 BOX | 0.807075 |
| 54 C36 | 8.7710 | -0.7960 | -0.9050 ca | 1 BOX | -0.153938 |
| 55 C37 | 8.5140 | 0.0700 | 0.1950 ca | 1 BOX | 0.213805 |
| 56 C38 | 9.5410 | 1.0520 | 0.5560 ca | 1 BOX | 0.263385 |
| 57 C39 | 10.7250 | 1.1200 | -0.1820 ca | 1 BOX | -0.230920 |
| 58 H19 | 11.4600 | 1.8700 | 0.1160 ha | 1 BOX | 0.042985 |
| 59 C40 | 10.9580 | 0.2480 | -1.2690 ca | 1 BOX | -0.198917 |
| 60 H20 | 11.8920 | 0.3190 | -1.8370 ha | 1 BOX | 0.028481 |
| 61 C41 | 10.0050 | -0.6870 | -1.6160 ca | 1 BOX | -0.301874 |
| 62 H21 | 10.1490 | -1.3680 | -2.4490 ha | 1 BOX | 0.116258 |
| 63 C42 | 3.9320 | -8.3740 | -3.6240 c | 1 BOX | 0.807075 |
| 64 C43 | 2.7660 | -9.0530 | -4.2560 ca | 1 BOX | -0.153938 |
| 65 C44 | 2.9440 | -10.4620 | -4.4270 ca | 1 BOX | -0.301874 |
| 66 H22 | 3.8830 | -10.8820 | -4.0800 ha | 1 BOX | 0.116258 |
| 67 C45 | 1.9700 | -11.2500 | -5.0000 ca | 1 BOX | -0.198917 |
| 68 H23 | 2.1230 | -12.3310 | -5.1020 ha | 1 BOX | 0.028481 |
| 69 C46 | 0.7680 | -10.6620 | -5.4560 ca | 1 BOX | -0.230920 |
| 70 H24 | -0.0150 | -11.2560 | -5.9290 ha | 1 BOX | 0.042985 |
| 71 C47 | 0.5460 | -9.2890 | -5.3320 ca | 1 BOX | 0.263385 |
| 72 C48 | 1.5530 | -8.4390 | -4.6830 ca | 1 BOX | 0.213805 |
| 73 C49 | -1.7160 | 0.5070 | -4.1680 ca | 1 BOX | -0.195908 |
| 74 H25 | -1.7510 | 0.5920 | -3.0850 ha | 1 BOX | 0.135251 |
| 75 C50 | -1.1060 | -0.6270 | -4.7480 ca | 1 BOX | -0.181359 |
| 76 H26 | -0.6680 | -1.3910 | -4.1110 ha | 1 BOX | 0.114661 |
| 77 C51 | -2.2480 | 1.5090 | -4.9500 ca | 1 BOX | -0.143496 |
| 78 H27 | -2.7110 | 2.3750 | -4.4870 ha | 1 BOX | 0.139616 |
| 79 C52 | -1.0380 | -0.7810 | -6.1210 ca | 1 BOX | 0.393566 |
| 80 C53 | -2.2170 | 1.4050 | -6.3660 ca | 1 BOX | 0.001430 |
| 81 C54 | -1.6270 | 0.2280 | -6.9670 ca | 1 BOX | 0.001430 |
| 82 C55 | -2.7990 | 2.4160 | -7.2160 ca | 1 BOX | 0.393566 |
| 83 C56 | -1.6920 | 0.0740 | -8.3770 ca | 1 BOX | -0.143496 |
| 84 H28 | -1.2880 | -0.8220 | -8.8260 ha | 1 BOX | 0.139616 |
| 85 C57 | -2.7840 | 2.2330 | -8.5890 ca | 1 BOX | -0.181359 |
| 86 H29 | -3.2310 | 2.9910 | -9.2190 ha | 1 BOX | 0.114661 |
| 87 C58 | -2.2540 | 1.0570 | -9.1580 ca | 1 BOX | -0.195908 |
| 88 H30 | -2.2890 | 0.9310 | -10.2410 ha | 1 BOX | 0.135251 |
| 89 C59 | -4.6510 | 4.0480 | -7.1250 c | 1 BOX | 0.807075 |
| 90 C60 | -5.2380 | 5.2310 | -6.4360 ca | 1 BOX | -0.153938 |
| 91 C61 | -6.5800 | 5.5270 | -6.8250 ca | 1 BOX | -0.301874 |

| | | | | | | | |
|---|---|---|---|---|---|---|---|
| 92 | H31 | -7.0340 | 4.8680 | -7.5590 | ha | 1 BOX | 0.116258 |
| 93 | C62 | -7.2690 | 6.5910 | -6.2840 | ca | 1 BOX | -0.198917 |
| 94 | H32 | -8.3040 | 6.7880 | -6.5850 | ha | 1 BOX | 0.028481 |
| 95 | C63 | -6.6410 | 7.4260 | -5.3330 | ca | 1 BOX | -0.230920 |
| 96 | H33 | -7.1600 | 8.2780 | -4.8910 | ha | 1 BOX | 0.042985 |
| 97 | C64 | -5.3270 | 7.1930 | -4.9240 | ca | 1 BOX | 0.263385 |
| 98 | C65 | -4.5860 | 6.0530 | -5.4740 | ca | 1 BOX | 0.213805 |
| 99 | C66 | 0.5820 | -1.9980 | -7.5680 | c | 1 BOX | 0.807075 |
| 100 | C67 | 1.0830 | -3.3420 | -7.9860 | ca | 1 BOX | -0.153938 |
| 101 | C68 | 0.7870 | -4.5810 | -7.3560 | ca | 1 BOX | 0.213805 |
| 102 | C69 | 1.9030 | -3.3200 | -9.1520 | ca | 1 BOX | -0.301874 |
| 103 | H34 | 2.1210 | -2.3460 | -9.5780 | ha | 1 BOX | 0.116258 |
| 104 | C70 | 2.3860 | -4.4850 | -9.7120 | ca | 1 BOX | -0.198917 |
| 105 | H35 | 3.0140 | -4.4460 | -10.6080 | ha | 1 BOX | 0.028481 |
| 106 | C71 | 1.2820 | -5.8080 | -7.9760 | ca | 1 BOX | 0.263385 |
| 107 | C72 | 2.0690 | -5.7300 | -9.1280 | ca | 1 BOX | -0.230920 |
| 108 | H36 | 2.4290 | -6.6680 | -9.5520 | ha | 1 BOX | 0.042985 |
| 109 | C73 | -2.7780 | -3.5690 | 0.4920 | ca | 1 BOX | -0.195908 |
| 110 | H37 | -2.4120 | -2.6180 | 0.1170 | ha | 1 BOX | 0.135251 |
| 111 | C74 | -2.9990 | -3.7180 | 1.8780 | ca | 1 BOX | -0.181359 |
| 112 | H38 | -2.7910 | -2.8900 | 2.5500 | ha | 1 BOX | 0.114661 |
| 113 | C75 | -3.0060 | -4.6100 | -0.3810 | ca | 1 BOX | -0.143496 |
| 114 | H39 | -2.8360 | -4.4820 | -1.4450 | ha | 1 BOX | 0.139616 |
| 115 | C76 | -3.4690 | -4.9080 | 2.4100 | ca | 1 BOX | 0.393566 |
| 116 | C77 | -3.4910 | -5.8540 | 0.1000 | ca | 1 BOX | 0.001430 |
| 117 | C78 | -3.7520 | -6.0070 | 1.5160 | ca | 1 BOX | 0.001430 |
| 118 | C79 | -3.7780 | -6.9500 | -0.7940 | ca | 1 BOX | 0.393566 |
| 119 | C80 | -4.3360 | -7.2160 | 1.9750 | ca | 1 BOX | -0.143496 |
| 120 | H40 | -4.5610 | -7.3260 | 3.0260 | ha | 1 BOX | 0.139616 |
| 121 | C81 | -4.2920 | -8.1250 | -0.2750 | ca | 1 BOX | -0.181359 |
| 122 | H41 | -4.5130 | -8.9380 | -0.9560 | ha | 1 BOX | 0.114661 |
| 123 | C82 | -4.5880 | -8.2450 | 1.0970 | ca | 1 BOX | -0.195908 |
| 124 | H42 | -5.0240 | -9.1750 | 1.4660 | ha | 1 BOX | 0.135251 |
| 125 | C83 | -4.6070 | -7.2220 | -3.0920 | c | 1 BOX | 0.807075 |
| 126 | C84 | -4.3450 | -7.1150 | -4.5550 | ca | 1 BOX | -0.153938 |
| 127 | C85 | -3.0990 | -6.8170 | -5.1780 | ca | 1 BOX | 0.213805 |
| 128 | C86 | -5.4920 | -7.3880 | -5.3630 | ca | 1 BOX | -0.301874 |
| 129 | H43 | -6.4190 | -7.5990 | -4.8400 | ha | 1 BOX | 0.116258 |
| 130 | C87 | -5.4250 | -7.3850 | -6.7390 | ca | 1 BOX | -0.198917 |
| 131 | H44 | -6.3220 | -7.5800 | -7.3360 | ha | 1 BOX | 0.028481 |
| 132 | C88 | -4.1940 | -7.1250 | -7.3810 | ca | 1 BOX | -0.230920 |
| 133 | H45 | -4.1030 | -7.1270 | -8.4680 | ha | 1 BOX | 0.042985 |
| 134 | C89 | -3.0390 | -6.8600 | -6.6430 | ca | 1 BOX | 0.263385 |
| 135 | C90 | -3.2750 | -5.9070 | 4.7000 | c | 1 BOX | 0.807075 |
| 136 | C91 | -3.6290 | -5.7090 | 6.1400 | ca | 1 BOX | -0.153938 |
| 137 | C92 | -3.4250 | -6.8580 | 6.9590 | ca | 1 BOX | -0.301874 |
| 138 | H46 | -3.0030 | -7.7340 | 6.4760 | ha | 1 BOX | 0.116258 |
| 139 | C93 | -4.1500 | -4.5200 | 6.7160 | ca | 1 BOX | 0.213805 |

| | | | | | | | |
|---|---|---|---|---|---|---|---|
| 140 | C94 | -3.7570 | -6.8540 | 8.2980 | ca | 1 BOX | -0.198917 |
| 141 | H47 | -3.5940 | -7.7480 | 8.9090 | ha | 1 BOX | 0.028481 |
| 142 | C95 | -4.3050 | -5.6910 | 8.8840 | ca | 1 BOX | -0.230920 |
| 143 | H48 | -4.5810 | -5.6600 | 9.9400 | ha | 1 BOX | 0.042985 |
| 144 | C96 | -4.5120 | -4.5320 | 8.1310 | ca | 1 BOX | 0.263385 |
| 145 | C97 | -3.3160 | 2.9650 | 0.0420 | ca | 1 BOX | -0.181359 |
| 146 | H49 | -2.7140 | 2.9960 | -0.8630 | ha | 1 BOX | 0.114661 |
| 147 | C98 | -3.7980 | 1.7290 | 0.5280 | ca | 1 BOX | -0.195908 |
| 148 | H50 | -3.5690 | 0.8200 | -0.0230 | ha | 1 BOX | 0.135251 |
| 149 | C99 | -3.5810 | 4.1480 | 0.7070 | ca | 1 BOX | 0.393566 |
| 150 | C100 | -4.5390 | 1.6600 | 1.6870 | ca | 1 BOX | -0.143496 |
| 151 | H51 | -4.9100 | 0.7080 | 2.0540 | ha | 1 BOX | 0.139616 |
| 152 | C101 | -4.3820 | 4.1130 | 1.9070 | ca | 1 BOX | 0.001430 |
| 153 | C102 | -4.8530 | 2.8430 | 2.4080 | ca | 1 BOX | 0.001430 |
| 154 | C103 | -4.7730 | 5.2990 | 2.5810 | ca | 1 BOX | -0.143496 |
| 155 | H52 | -4.4390 | 6.2550 | 2.2000 | ha | 1 BOX | 0.139616 |
| 156 | C104 | -5.6720 | 2.8120 | 3.5900 | ca | 1 BOX | 0.393566 |
| 157 | C105 | -5.5640 | 5.2360 | 3.7060 | ca | 1 BOX | -0.195908 |
| 158 | H53 | -5.8520 | 6.1550 | 4.2160 | ha | 1 BOX | 0.135251 |
| 159 | C106 | -6.0030 | 3.9970 | 4.2180 | ca | 1 BOX | -0.181359 |
| 160 | H54 | -6.6380 | 3.9550 | 5.0960 | ha | 1 BOX | 0.114661 |
| 161 | C107 | -2.3380 | 6.2970 | 0.8000 | c | 1 BOX | 0.807075 |
| 162 | C108 | -2.0630 | 7.5810 | 0.0860 | ca | 1 BOX | -0.153938 |
| 163 | C109 | -2.2690 | 7.8160 | -1.2970 | ca | 1 BOX | 0.213805 |
| 164 | C110 | -1.6390 | 8.6470 | 0.9280 | ca | 1 BOX | -0.301874 |
| 165 | H55 | -1.4610 | 8.4100 | 1.9720 | ha | 1 BOX | 0.116258 |
| 166 | C111 | -1.4770 | 9.9250 | 0.4320 | ca | 1 BOX | -0.198917 |
| 167 | H56 | -1.1460 | 10.7320 | 1.0910 | ha | 1 BOX | 0.028481 |
| 168 | C112 | -2.1280 | 9.1740 | -1.8040 | ca | 1 BOX | 0.263385 |
| 169 | C113 | -1.7310 | 10.1900 | -0.9310 | ca | 1 BOX | -0.230920 |
| 170 | H57 | -1.6180 | 11.1930 | -1.3460 | ha | 1 BOX | 0.042985 |
| 171 | C114 | -7.5770 | 1.4160 | 4.2540 | c | 1 BOX | 0.807075 |
| 172 | C115 | -8.0540 | 0.1280 | 4.8380 | ca | 1 BOX | -0.153938 |
| 173 | C116 | -9.4540 | -0.1030 | 4.6930 | ca | 1 BOX | -0.301874 |
| 174 | H58 | -10.0230 | 0.6520 | 4.1600 | ha | 1 BOX | 0.116258 |
| 175 | C117 | -7.2500 | -0.8230 | 5.5240 | ca | 1 BOX | 0.213805 |
| 176 | C118 | -10.0520 | -1.2370 | 5.2040 | ca | 1 BOX | -0.198917 |
| 177 | H59 | -11.1250 | -1.4050 | 5.0630 | ha | 1 BOX | 0.028481 |
| 178 | C119 | -7.9050 | -2.0030 | 6.0930 | ca | 1 BOX | 0.263385 |
| 179 | C120 | -9.2770 | -2.1870 | 5.9060 | ca | 1 BOX | -0.230920 |
| 180 | H60 | -9.7250 | -3.0860 | 6.3310 | ha | 1 BOX | 0.042985 |
| 181 | C121 | 2.0810 | -0.2520 | 4.7760 | ca | 1 BOX | -0.181359 |
| 182 | H61 | 2.5270 | -0.2700 | 3.7850 | ha | 1 BOX | 0.114661 |
| 183 | C122 | 0.6840 | -0.1090 | 4.9160 | ca | 1 BOX | -0.195908 |
| 184 | H62 | 0.0730 | 0.0010 | 4.0240 | ha | 1 BOX | 0.135251 |
| 185 | C123 | 2.9020 | -0.3880 | 5.8830 | ca | 1 BOX | 0.393566 |
| 186 | C124 | 0.0840 | -0.1270 | 6.1570 | ca | 1 BOX | -0.143496 |
| 187 | H63 | -0.9930 | -0.0220 | 6.2370 | ha | 1 BOX | 0.139616 |

| | | | | | | | | |
|---|---|---|---|---|---|---|---|---|
| 188 | C125 | 2.3110 | -0.3480 | 7.1980 | ca | 1 | BOX | 0.001430 |
| 189 | C126 | 0.8730 | -0.2510 | 7.3310 | ca | 1 | BOX | 0.001430 |
| 190 | C127 | 3.1120 | -0.3530 | 8.3700 | ca | 1 | BOX | -0.143496 |
| 191 | H64 | 4.1870 | -0.3800 | 8.2700 | ha | 1 | BOX | 0.139616 |
| 192 | C128 | 0.2850 | -0.2450 | 8.6520 | ca | 1 | BOX | 0.393566 |
| 193 | C129 | 2.5200 | -0.3380 | 9.6110 | ca | 1 | BOX | -0.195908 |
| 194 | H65 | 3.1440 | -0.3540 | 10.5060 | ha | 1 | BOX | 0.135251 |
| 195 | C130 | 1.1180 | -0.3130 | 9.7590 | ca | 1 | BOX | -0.181359 |
| 196 | H66 | 0.6760 | -0.3000 | 10.7440 | ha | 1 | BOX | 0.114661 |
| 197 | C131 | -1.7390 | 0.5080 | 9.8750 | c | 1 | BOX | 0.807075 |
| 198 | C132 | -3.2240 | 0.4520 | 9.9700 | ca | 1 | BOX | -0.153938 |
| 199 | C133 | -4.1030 | -0.2320 | 9.0810 | ca | 1 | BOX | 0.213805 |
| 200 | C134 | -5.5380 | -0.2320 | 9.3980 | ca | 1 | BOX | 0.263385 |
| 201 | C135 | -6.0020 | 0.4640 | 10.5170 | ca | 1 | BOX | -0.230920 |
| 202 | H67 | -7.0760 | 0.4470 | 10.7050 | ha | 1 | BOX | 0.042985 |
| 203 | C136 | -5.1110 | 1.1570 | 11.3670 | ca | 1 | BOX | -0.198917 |
| 204 | H68 | -5.4980 | 1.7040 | 12.2350 | ha | 1 | BOX | 0.028481 |
| 205 | C137 | -3.7590 | 1.1460 | 11.1000 | ca | 1 | BOX | -0.301874 |
| 206 | H69 | -3.0480 | 1.6620 | 11.7370 | ha | 1 | BOX | 0.116258 |
| 207 | C138 | 5.0960 | -1.5330 | 6.1640 | c | 1 | BOX | 0.807075 |
| 208 | C139 | 6.5140 | -1.5860 | 5.7060 | ca | 1 | BOX | -0.153938 |
| 209 | C140 | 7.0900 | -0.8080 | 4.6630 | ca | 1 | BOX | 0.213805 |
| 210 | C141 | 8.5040 | -1.0160 | 4.3450 | ca | 1 | BOX | 0.263385 |
| 211 | C142 | 9.2460 | -1.9490 | 5.0750 | ca | 1 | BOX | -0.230920 |
| 212 | H70 | 10.2960 | -2.0730 | 4.8070 | ha | 1 | BOX | 0.042985 |
| 213 | C143 | 8.6520 | -2.7090 | 6.1070 | ca | 1 | BOX | -0.198917 |
| 214 | H71 | 9.2510 | -3.4420 | 6.6600 | ha | 1 | BOX | 0.028481 |
| 215 | C144 | 7.3180 | -2.5350 | 6.4090 | ca | 1 | BOX | -0.301874 |
| 216 | H72 | 6.8260 | -3.1130 | 7.1850 | ha | 1 | BOX | 0.116258 |
| 217 | N1 | 2.1240 | 7.5970 | -3.7780 | n | 1 | BOX | -0.990800 |
| 218 | H73 | 1.1650 | 7.2710 | -3.6350 | hn | 1 | BOX | 0.582649 |
| 219 | N2 | 5.0500 | 4.9080 | 1.0620 | n | 1 | BOX | -0.990800 |
| 220 | H74 | 5.1550 | 3.9240 | 1.3090 | hn | 1 | BOX | 0.582649 |
| 221 | N3 | 3.7860 | -7.0570 | -3.2120 | n | 1 | BOX | -0.990800 |
| 222 | H75 | 2.8970 | -6.6360 | -3.4870 | hn | 1 | BOX | 0.582649 |
| 223 | N4 | 6.5750 | -1.9350 | -0.9120 | n | 1 | BOX | -0.990800 |
| 224 | H76 | 6.3360 | -1.2340 | -0.2080 | hn | 1 | BOX | 0.582649 |
| 225 | N5 | -6.2130 | 1.5880 | 4.0910 | n | 1 | BOX | -0.990800 |
| 226 | H77 | -5.6180 | 0.8570 | 4.4830 | hn | 1 | BOX | 0.582649 |
| 227 | N6 | -3.1370 | 5.3740 | 0.1410 | n | 1 | BOX | -0.990800 |
| 228 | H78 | -3.1920 | 5.5050 | -0.8720 | hn | 1 | BOX | 0.582649 |
| 229 | N7 | -3.7380 | -4.9530 | 3.8060 | n | 1 | BOX | -0.990800 |
| 230 | H79 | -4.0520 | -4.0880 | 4.2560 | hn | 1 | BOX | 0.582649 |
| 231 | N8 | -3.6130 | -6.8310 | -2.2050 | n | 1 | BOX | -0.990800 |
| 232 | H80 | -2.7390 | -6.5100 | -2.6250 | hn | 1 | BOX | 0.582649 |
| 233 | N9 | -0.4550 | -1.9680 | -6.6470 | n | 1 | BOX | -0.990800 |
| 234 | H81 | -0.6020 | -2.8500 | -6.1480 | hn | 1 | BOX | 0.582649 |
| 235 | N10 | -1.1220 | -0.1520 | 8.8220 | n | 1 | BOX | -0.990800 |

| | | | | | | | |
|---|---|---|---|---|---|---|---|
| 236 H82 | -1.7630 | -0.6020 | 8.1640 | hn | 1 | BOX | 0.582649 |
| 237 N11 | -3.4310 | 3.5660 | -6.6690 | n | 1 | BOX | -0.990800 |
| 238 H83 | -3.0020 | 4.1050 | -5.9140 | hn | 1 | BOX | 0.582649 |
| 239 N12 | 4.3060 | -0.4970 | 5.6790 | n | 1 | BOX | -0.990800 |
| 240 H84 | 4.7220 | 0.0020 | 4.8870 | hn | 1 | BOX | 0.582649 |
| 241 O1 | 0.9590 | -6.9500 | -7.4200 | Y1 | 1 | BOX | -0.496622 |
| 242 O2 | 0.0840 | -4.7240 | -6.2440 | Y2 | 1 | BOX | -0.547732 |
| 243 O3 | -1.9760 | -6.5240 | -4.5560 | Y3 | 1 | BOX | -0.547732 |
| 244 O4 | -1.8810 | -6.6380 | -7.2130 | Y4 | 1 | BOX | -0.496622 |
| 245 O5 | -0.5440 | -8.7150 | -5.7690 | Y5 | 1 | BOX | -0.496622 |
| 246 O6 | 1.2550 | -7.1660 | -4.5680 | Y6 | 1 | BOX | -0.547732 |
| 247 O7 | -0.6170 | 7.5890 | -4.3720 | Y7 | 1 | BOX | -0.547732 |
| 248 O8 | -2.5920 | 8.6020 | -5.8300 | Y8 | 1 | BOX | -0.496622 |
| 249 O9 | -3.3580 | 5.8920 | -5.0350 | Y9 | 1 | BOX | -0.547732 |
| 250 O10 | -4.7130 | 7.9530 | -4.0510 | Z1 | 1 | BOX | -0.496622 |
| 251 O11 | -2.4000 | 9.3890 | -3.0720 | Z2 | 1 | BOX | -0.496622 |
| 252 O12 | -2.6020 | 6.8840 | -2.1790 | Z3 | 1 | BOX | -0.547732 |
| 253 O13 | 6.4560 | 0.0940 | 3.9400 | Z4 | 1 | BOX | -0.547732 |
| 254 O14 | 9.0230 | -0.3070 | 3.3770 | Z5 | 1 | BOX | -0.496622 |
| 255 O15 | 7.4270 | 0.0710 | 0.9370 | Z6 | 1 | BOX | -0.547732 |
| 256 O16 | 9.2960 | 1.8440 | 1.5710 | Z7 | 1 | BOX | -0.496622 |
| 257 O17 | 8.3970 | 2.3290 | 4.2360 | Z8 | 1 | BOX | -0.496622 |
| 258 O18 | 6.6350 | 2.8700 | 2.3330 | Z9 | 1 | BOX | -0.547732 |
| 259 O19 | -7.1590 | -2.8510 | 6.7590 | A1 | 1 | BOX | -0.496622 |
| 260 O20 | -5.9500 | -0.7250 | 5.7190 | A2 | 1 | BOX | -0.547732 |
| 261 O21 | -4.3440 | -3.3830 | 6.0730 | A3 | 1 | BOX | -0.547732 |
| 262 O22 | -5.0260 | -3.4370 | 8.6400 | A4 | 1 | BOX | -0.496622 |
| 263 O23 | -3.7450 | -0.8830 | 7.9980 | A5 | 1 | BOX | -0.547732 |
| 264 O24 | -6.3390 | -0.8920 | 8.6040 | A6 | 1 | BOX | -0.496622 |
| 265 O25 | -8.3780 | 2.2950 | 3.8920 | o | 1 | BOX | -0.677284 |
| 266 O26 | -5.6970 | -7.6490 | -2.6700 | o | 1 | BOX | -0.677284 |
| 267 O27 | -5.2490 | 3.4930 | -8.0640 | o | 1 | BOX | -0.677284 |
| 268 O28 | 3.3480 | 9.1330 | -4.9780 | o | 1 | BOX | -0.677284 |
| 269 O29 | 1.0130 | -0.9550 | -8.0770 | o | 1 | BOX | -0.677284 |
| 270 O30 | 8.1900 | -2.5840 | -2.3820 | o | 1 | BOX | -0.677284 |
| 271 O31 | 5.7900 | 7.0190 | 1.4680 | o | 1 | BOX | -0.677284 |
| 272 O32 | 4.6440 | -2.3520 | 6.9790 | o | 1 | BOX | -0.677284 |
| 273 O33 | -2.6600 | -6.9110 | 4.3150 | o | 1 | BOX | -0.677284 |
| 274 O34 | -1.9610 | 6.0990 | 1.9610 | o | 1 | BOX | -0.677284 |
| 275 O35 | 4.9990 | -8.9980 | -3.4520 | o | 1 | BOX | -0.677284 |
| 276 O36 | -1.0720 | 1.1190 | 10.7330 | o | 1 | BOX | -0.677284 |
| 277 GA1 | -0.3600 | -6.7330 | -5.9340 | M1 | 1 | BOX | 0.839067 |
| 278 GA2 | -2.7400 | 7.6830 | -4.0560 | M2 | 1 | BOX | 0.839067 |
| 279 GA3 | 7.8150 | 1.1550 | 2.7230 | M3 | 1 | BOX | 0.839067 |
| 280 GA4 | -5.4000 | -2.0400 | 7.2610 | M4 | 1 | BOX | 0.839067 |

#

**7. Cartesian coordinates:**

#
$NEt_4^+$ encapsulated in the metallocage (neutral complex with $K^+$ counter ions)

320 atoms

| | | | |
|---|---|---|---|
| C | 0.406481 | -1.268382 | 5.839094 |
| H | 0.611211 | -2.330805 | 5.909344 |
| C | -0.047501 | 1.520207 | 5.754510 |
| C | 1.376728 | -0.378129 | 6.241795 |
| H | 2.326579 | -0.747956 | 6.610860 |
| C | 1.167531 | 1.013803 | 6.184148 |
| H | 1.958909 | 1.689095 | 6.472151 |
| C | -0.850929 | -0.795243 | 5.370779 |
| C | -1.100607 | 0.622364 | 5.348816 |
| C | -1.889832 | -1.691491 | 4.921930 |
| C | -2.375670 | 1.088263 | 4.917917 |
| H | -2.591570 | 2.145947 | 4.820643 |
| C | -3.115972 | -1.189486 | 4.513678 |
| H | -3.886081 | -1.864779 | 4.170136 |
| C | -3.351605 | 0.197293 | 4.528126 |
| H | -4.312547 | 0.569790 | 4.199452 |
| C | -2.477027 | -4.117589 | 5.019310 |
| C | -1.854838 | -5.471412 | 5.080822 |
| C | -0.465907 | -5.710929 | 4.973289 |
| C | -2.729199 | -6.570065 | 5.270999 |
| H | -3.792177 | -6.372328 | 5.352619 |
| C | 0.032932 | -7.053651 | 5.072062 |
| C | -0.859702 | -8.107188 | 5.253972 |
| H | -0.465870 | -9.119021 | 5.317781 |
| C | -2.242299 | -7.863490 | 5.353119 |
| H | -2.925968 | -8.695840 | 5.496646 |
| C | 0.314788 | 3.931634 | 6.348405 |
| C | -0.312273 | 5.278287 | 6.197419 |
| C | 0.386051 | 6.376228 | 6.756244 |
| H | 1.346721 | 6.191956 | 7.223835 |
| C | -1.560471 | 5.508101 | 5.574332 |
| C | -0.142887 | 7.655659 | 6.705351 |
| H | 0.409106 | 8.489953 | 7.129278 |
| C | -2.117851 | 6.827695 | 5.555534 |
| C | -1.397012 | 7.883073 | 6.111101 |
| H | -1.823752 | 8.882605 | 6.074524 |
| C | 4.566316 | -1.527685 | -1.234412 |
| C | 3.851782 | -2.803329 | 1.181439 |
| H | 3.511152 | -3.319321 | 2.073198 |
| C | 3.841747 | -3.488669 | -0.014361 |
| H | 3.552886 | -4.532099 | -0.030485 |
| C | 4.208597 | -2.866050 | -1.223669 |
| H | 4.203644 | -3.430571 | -2.144284 |

| | | | |
|---|---|---|---|
| C | 4.226149 | -1.429160 | 1.220288 |
| C | 4.537532 | -0.760240 | -0.012729 |
| C | 4.305498 | -0.680733 | 2.450226 |
| C | 4.815221 | 0.635681 | 0.014623 |
| H | 4.958386 | 1.203738 | -0.896905 |
| C | 4.617374 | 0.667103 | 2.432550 |
| H | 4.646881 | 1.225633 | 3.356414 |
| C | 4.852596 | 1.320696 | 1.209388 |
| H | 5.041421 | 2.385227 | 1.205217 |
| C | 4.675673 | -1.124259 | 4.873431 |
| C | 4.385609 | -2.110878 | 5.955653 |
| C | 3.660264 | -3.308249 | 5.757195 |
| C | 3.455762 | -4.212932 | 6.849873 |
| C | 3.951536 | -3.881909 | 8.109619 |
| H | 3.780655 | -4.569510 | 8.934622 |
| C | 4.656739 | -2.680813 | 8.307628 |
| H | 5.030487 | -2.436169 | 9.298055 |
| C | 4.876575 | -1.813287 | 7.250267 |
| H | 5.420110 | -0.886493 | 7.395892 |
| C | 5.627834 | -1.410786 | -3.496720 |
| C | 6.184313 | -0.419816 | -4.462639 |
| C | 6.548110 | -0.899326 | -5.744393 |
| H | 6.457532 | -1.961372 | -5.943792 |
| C | 6.981150 | -0.027855 | -6.730647 |
| H | 7.246789 | -0.406974 | -7.713647 |
| C | 7.060686 | 1.352710 | -6.472822 |
| H | 7.376438 | 2.046649 | -7.248435 |
| C | 6.736705 | 1.860343 | -5.214352 |
| C | 6.326487 | 0.956646 | -4.180436 |
| C | 1.747809 | 5.788289 | 3.215660 |
| H | 1.548750 | 6.199388 | 4.200961 |
| C | 3.082980 | 5.508049 | 2.852603 |
| H | 3.886850 | 5.727237 | 3.539740 |
| C | 0.705909 | 5.590397 | 2.336431 |
| H | -0.299400 | 5.864602 | 2.637808 |
| C | 3.368747 | 4.984707 | 1.601325 |
| C | 0.953633 | 5.026728 | 1.053382 |
| C | 2.295831 | 4.659757 | 0.695034 |
| C | -0.094060 | 4.781881 | 0.100137 |
| C | 2.525897 | 3.978337 | -0.530039 |
| H | 3.512402 | 3.632731 | -0.809444 |
| C | 0.170321 | 4.088502 | -1.064602 |
| H | -0.654849 | 3.823078 | -1.710493 |
| C | 1.479052 | 3.678233 | -1.370231 |
| H | 1.666504 | 3.103624 | -2.264492 |
| C | -2.273222 | 5.816409 | -0.470166 |
| C | -3.520491 | 6.348117 | 0.138035 |
| C | -4.408884 | 7.069339 | -0.697683 |

| | | | |
|---|---|---|---|
| H | -4.126695 | 7.238904 | -1.731192 |
| C | -5.620556 | 7.531651 | -0.212548 |
| H | -6.293698 | 8.083233 | -0.863316 |
| C | -5.995535 | 7.279466 | 1.121626 |
| H | -6.955404 | 7.620235 | 1.503042 |
| C | -5.143956 | 6.587369 | 1.979562 |
| C | -3.870358 | 6.137174 | 1.492064 |
| C | 5.871634 | 4.804020 | 1.780423 |
| C | 7.068230 | 4.610973 | 0.906885 |
| C | 7.006835 | 4.154361 | -0.431694 |
| C | 8.324873 | 4.952390 | 1.457864 |
| H | 8.357473 | 5.295565 | 2.486250 |
| C | 9.484412 | 4.878690 | 0.701068 |
| H | 10.440087 | 5.152342 | 1.139262 |
| C | 8.204307 | 4.117532 | -1.217318 |
| C | 9.426629 | 4.467376 | -0.642761 |
| H | 10.324857 | 4.419438 | -1.253970 |
| C | 3.125559 | -1.413111 | -6.056834 |
| H | 4.126024 | -1.564691 | -6.445791 |
| C | 2.185876 | -2.461233 | -6.152419 |
| H | 2.462023 | -3.393884 | -6.628453 |
| C | 2.784877 | -0.195822 | -5.507354 |
| H | 3.518808 | 0.601893 | -5.461522 |
| C | 0.897744 | -2.289875 | -5.681487 |
| C | 1.483003 | 0.003239 | -4.973256 |
| C | 0.515143 | -1.061594 | -5.041038 |
| C | 1.083697 | 1.239534 | -4.366866 |
| C | -0.777849 | -0.869243 | -4.475078 |
| H | -1.528395 | -1.648131 | -4.527978 |
| C | -0.153478 | 1.354639 | -3.759455 |
| H | -0.413092 | 2.271816 | -3.250838 |
| C | -1.085147 | 0.301054 | -3.815144 |
| H | -2.047890 | 0.423187 | -3.337722 |
| C | 1.552237 | 3.603063 | -4.827946 |
| C | 2.527546 | 4.706062 | -4.698729 |
| C | 3.783727 | 4.556128 | -4.063907 |
| C | 2.179220 | 5.963185 | -5.257330 |
| H | 1.214897 | 6.066682 | -5.743527 |
| C | 3.061763 | 7.027547 | -5.202575 |
| H | 2.787721 | 7.985875 | -5.635287 |
| C | 4.327410 | 6.874975 | -4.599512 |
| H | 5.028284 | 7.706001 | -4.564732 |
| C | 4.704877 | 5.658869 | -4.038047 |
| C | 0.029707 | -4.626481 | -5.739983 |
| C | -1.219945 | -5.399821 | -5.976537 |
| C | -1.094988 | -6.768605 | -6.333650 |
| H | -0.100272 | -7.187162 | -6.464170 |
| C | -2.505751 | -4.825250 | -5.876115 |

| | | | |
|---|---|---|---|
| C | -2.221249 | -7.533625 | -6.597621 |
| H | -2.115503 | -8.577369 | -6.880316 |
| C | -3.503851 | -6.954664 | -6.536324 |
| H | -4.388950 | -7.543862 | -6.764506 |
| C | -3.662576 | -5.609529 | -6.205498 |
| C | -4.472400 | -0.317275 | 0.567353 |
| H | -4.441939 | -0.885425 | 1.491965 |
| C | -4.121874 | -0.928173 | -0.657778 |
| H | -3.811930 | -1.964822 | -0.662074 |
| C | -4.852898 | 1.011091 | 0.596563 |
| C | -4.169431 | -0.218840 | -1.839804 |
| H | -3.922299 | -0.729083 | -2.764468 |
| C | -4.848965 | 1.803426 | -0.598151 |
| C | -4.518537 | 1.162507 | -1.843726 |
| C | -5.163199 | 3.188775 | -0.577907 |
| H | -5.399903 | 3.677995 | 0.360677 |
| C | -4.567658 | 1.951280 | -3.044139 |
| C | -5.173586 | 3.913119 | -1.749736 |
| H | -5.422353 | 4.967840 | -1.730330 |
| C | -4.891412 | 3.295984 | -2.985519 |
| H | -4.971378 | 3.865307 | -3.904701 |
| C | -6.277626 | 1.216788 | 2.597325 |
| C | -6.511802 | 2.007650 | 3.835187 |
| C | -5.682035 | 3.084195 | 4.227492 |
| C | -7.615525 | 1.658891 | 4.650362 |
| H | -8.240775 | 0.826107 | 4.346433 |
| C | -7.896028 | 2.365355 | 5.808528 |
| H | -8.747169 | 2.087913 | 6.424054 |
| C | -5.993768 | 3.827074 | 5.414073 |
| C | -7.087832 | 3.455371 | 6.191799 |
| H | -7.309773 | 4.024032 | 7.092363 |
| C | -3.745428 | 1.806004 | -5.388931 |
| C | -3.806564 | 0.943536 | -6.597722 |
| C | -3.632658 | 1.563646 | -7.864932 |
| H | -3.515216 | 2.643753 | -7.906896 |
| C | -4.050887 | -0.446353 | -6.532762 |
| C | -3.692650 | 0.812503 | -9.029756 |
| H | -3.570057 | 1.295831 | -9.995173 |
| C | -4.148971 | -1.210836 | -7.748100 |
| C | -3.945410 | -0.572963 | -8.970974 |
| H | -4.007495 | -1.162026 | -9.882841 |
| C | 0.252186 | -4.376342 | -1.608771 |
| H | 1.291910 | -4.288479 | -1.908242 |
| C | -0.764802 | -3.790440 | -2.391327 |
| H | -0.495169 | -3.263281 | -3.296183 |
| C | -0.065896 | -5.037841 | -0.436932 |
| C | -2.087011 | -3.880055 | -2.010750 |
| H | -2.840473 | -3.416646 | -2.636714 |

| | | | |
|---|---|---|---|
| C | -1.417797 | -5.083468 | 0.036929 |
| C | -2.448831 | -4.525389 | -0.794358 |
| C | -1.756629 | -5.620280 | 1.309162 |
| H | -0.985136 | -6.027000 | 1.954284 |
| C | -3.814576 | -4.628480 | -0.351465 |
| C | -3.066674 | -5.608943 | 1.736004 |
| H | -3.317353 | -6.000009 | 2.716277 |
| C | -4.102544 | -5.134506 | 0.903879 |
| H | -5.130393 | -5.196669 | 1.235277 |
| C | -6.054379 | -3.728134 | -1.004143 |
| C | -6.883907 | -3.420256 | -2.201530 |
| C | -6.335269 | -3.218244 | -3.486564 |
| C | -7.204067 | -2.922616 | -4.593150 |
| C | -8.575252 | -2.801861 | -4.372355 |
| H | -9.220211 | -2.567785 | -5.216077 |
| C | -9.117432 | -2.999960 | -3.087747 |
| H | -10.191722 | -2.931586 | -2.940033 |
| C | -8.288160 | -3.307896 | -2.019840 |
| H | -8.709012 | -3.537158 | -1.044241 |
| C | 1.878824 | -6.515728 | -0.145781 |
| C | 2.902389 | -6.985946 | 0.822658 |
| C | 2.981914 | -6.503090 | 2.148977 |
| C | 3.999139 | -7.008953 | 3.025586 |
| C | 4.910150 | -7.953066 | 2.555096 |
| H | 5.675556 | -8.326107 | 3.232570 |
| C | 4.832424 | -8.417796 | 1.226523 |
| H | 5.547948 | -9.155275 | 0.873705 |
| C | 3.846276 | -7.942982 | 0.377918 |
| H | 3.778704 | -8.299245 | -0.644330 |
| N | -0.312486 | 2.901692 | 5.686234 |
| H | -1.089585 | 3.208818 | 5.101975 |
| N | -1.603051 | -3.065034 | 4.905561 |
| H | -0.624953 | -3.352675 | 4.828219 |
| N | 4.985321 | -0.870880 | -2.403650 |
| H | 5.050815 | 0.145082 | -2.372121 |
| N | 4.071314 | -1.364457 | 3.659375 |
| H | 3.450498 | -2.174927 | 3.652525 |
| N | -4.370374 | 1.311533 | -4.282917 |
| H | -4.623626 | 0.323428 | -4.364679 |
| N | -5.236179 | 1.618732 | 1.816125 |
| H | -4.749883 | 2.462153 | 2.136456 |
| N | -0.093611 | -3.272141 | -5.864143 |
| H | -1.065658 | -2.958892 | -5.908246 |
| N | 1.948950 | 2.361029 | -4.420311 |
| H | 2.910436 | 2.292334 | -4.072109 |
| N | 4.671443 | 4.787347 | 1.119439 |
| H | 4.764336 | 4.539060 | 0.134359 |
| N | -4.823239 | -4.260007 | -1.261739 |

| | | | |
|---|---|---|---|
| H | -4.571355 | -4.225313 | -2.250987 |
| N | -1.392649 | 5.232946 | 0.395478 |
| H | -1.711223 | 5.217970 | 1.368185 |
| N | 0.953466 | -5.637979 | 0.333685 |
| H | 1.038189 | -5.407720 | 1.328759 |
| O | 8.081733 | 3.695781 | -2.489222 |
| O | 5.886666 | 3.737408 | -1.039143 |
| O | 4.188162 | 3.423459 | -3.489991 |
| O | 5.904294 | 5.452463 | -3.464488 |
| O | 6.785609 | 3.163100 | -4.916400 |
| O | 6.103946 | 1.488329 | -2.964065 |
| O | -2.303576 | 4.536608 | 5.011114 |
| O | -3.322343 | 6.981387 | 4.987891 |
| O | -3.074752 | 5.515754 | 2.370872 |
| O | -5.453703 | 6.310976 | 3.255139 |
| O | -5.174050 | 4.853726 | 5.726690 |
| O | -4.601914 | 3.474703 | 3.551213 |
| O | 2.158514 | -5.586039 | 2.658055 |
| O | 4.032070 | -6.507303 | 4.276616 |
| O | 3.164801 | -3.682078 | 4.564792 |
| O | 2.787139 | -5.346909 | 6.597397 |
| O | 1.359985 | -7.232486 | 4.977427 |
| O | 0.442517 | -4.742204 | 4.788689 |
| O | -4.434714 | -2.512157 | -7.634841 |
| O | -4.223094 | -1.102095 | -5.387325 |
| O | -2.715404 | -3.559432 | -5.512275 |
| O | -4.850682 | -4.992249 | -6.173812 |
| O | -5.029615 | -3.305130 | -3.745549 |
| O | -6.637920 | -2.771108 | -5.796695 |
| O | -3.170640 | 2.919755 | -5.414408 |
| O | 0.404638 | 3.788471 | -5.314498 |
| O | -2.042625 | 5.914213 | -1.696834 |
| O | 1.323385 | 3.763634 | 7.054435 |
| O | 5.974337 | 5.002825 | 3.003361 |
| O | 5.442927 | -0.165604 | 5.061182 |
| O | -3.710195 | -3.964169 | 5.097444 |
| O | 1.864859 | -6.916356 | -1.331518 |
| O | 1.109660 | -5.192195 | -5.463863 |
| O | -7.006158 | 0.248647 | 2.283124 |
| O | 5.718763 | -2.634622 | -3.689077 |
| O | -6.479270 | -3.515872 | 0.152628 |
| Ga | 6.136105 | 3.510434 | -3.033566 |
| Ga | -3.963446 | 5.286090 | 4.134215 |
| Ga | 2.287192 | -5.488418 | 4.658551 |
| Ga | -4.640538 | -3.041445 | -5.689978 |
| N | 0.144874 | 0.116862 | 1.135170 |
| C | -1.171678 | 0.579038 | 1.751749 |
| H | -1.910496 | -0.179820 | 1.498485 |

| | | | |
|---|---|---|---|
| H | -1.018033 | 0.552475 | 2.826977 |
| C | 0.621690 | -1.123594 | 1.891001 |
| H | 1.620858 | -1.339286 | 1.518391 |
| H | 0.709209 | -0.826379 | 2.935568 |
| C | -0.264112 | -2.350787 | 1.762808 |
| H | -0.342624 | -2.690029 | 0.729912 |
| H | -1.268080 | -2.195796 | 2.164179 |
| H | 0.204822 | -3.152349 | 2.343840 |
| C | -1.663408 | 1.953697 | 1.327106 |
| H | -1.910276 | 2.011019 | 0.264345 |
| H | -0.956394 | 2.746427 | 1.569958 |
| H | -2.572702 | 2.154745 | 1.897747 |
| C | 1.219058 | 1.196620 | 1.258829 |
| H | 0.924982 | 1.992734 | 0.578133 |
| H | 2.137708 | 0.752251 | 0.880100 |
| C | 1.445407 | 1.740895 | 2.661419 |
| H | 2.239416 | 2.490067 | 2.591230 |
| H | 1.777592 | 0.974818 | 3.363711 |
| H | 0.562390 | 2.233980 | 3.070855 |
| C | -0.085734 | -0.180300 | -0.347296 |
| H | -0.924917 | -0.876847 | -0.396346 |
| H | -0.407731 | 0.761636 | -0.794560 |
| C | 1.117938 | -0.733198 | -1.097664 |
| H | 0.786087 | -0.970426 | -2.112281 |
| H | 1.505747 | -1.653967 | -0.655630 |
| H | 1.930605 | -0.007154 | -1.174923 |
| K | -0.691760 | 1.983946 | -7.135697 |
| K | 5.784811 | -4.516742 | 3.633564 |
| K | -5.915299 | 7.481917 | 5.659444 |
| K | -7.754018 | -0.973639 | 0.080290 |
| K | 0.337959 | -7.086737 | -3.452386 |
| K | -3.462615 | 3.447851 | 7.336373 |
| K | -1.865627 | 4.896617 | -4.108136 |
| K | 8.376182 | 5.304391 | -4.693153 |
| K | -6.555179 | -4.161499 | -8.142938 |
| K | 7.989871 | 1.066682 | -1.053439 |
| K | 3.486381 | -7.989809 | 6.480901 |

Metallocage (with 11 $K^+$ counter ions)

291 atoms

| | | | |
|---|---|---|---|
| C | 0.523963 | -1.204699 | 5.989125 |
| H | 0.672772 | -2.273763 | 6.076401 |
| C | 0.167263 | 1.593481 | 5.890522 |
| C | 1.403247 | -0.358779 | 6.625055 |
| H | 2.237149 | -0.770254 | 7.183266 |
| C | 1.239352 | 1.039833 | 6.571880 |

| | | | |
|---|---|---|---|
| H | 1.937533 | 1.683877 | 7.085455 |
| C | -0.561466 | -0.681043 | 5.235544 |
| C | -0.748383 | 0.742799 | 5.168881 |
| C | -1.501564 | -1.539001 | 4.559028 |
| C | -1.848931 | 1.252190 | 4.419165 |
| H | -2.001155 | 2.318429 | 4.286974 |
| C | -2.567762 | -1.002042 | 3.860920 |
| H | -3.259663 | -1.657596 | 3.347479 |
| C | -2.732565 | 0.395824 | 3.796420 |
| H | -3.554750 | 0.809522 | 3.227160 |
| C | -2.226234 | -3.893367 | 4.904011 |
| C | -1.683351 | -5.220509 | 5.318469 |
| C | -0.303348 | -5.492682 | 5.445679 |
| C | -2.608530 | -6.247788 | 5.625502 |
| H | -3.667687 | -6.032025 | 5.534075 |
| C | 0.145461 | -6.790925 | 5.856763 |
| C | -0.795313 | -7.774267 | 6.148731 |
| H | -0.449646 | -8.757812 | 6.458414 |
| C | -2.172640 | -7.497825 | 6.034897 |
| H | -2.894501 | -8.276121 | 6.267063 |
| C | 0.251321 | 3.900896 | 6.869169 |
| C | -0.363427 | 5.254133 | 6.735681 |
| C | 0.149816 | 6.269934 | 7.579783 |
| H | 0.990939 | 6.030221 | 8.220867 |
| C | -1.456273 | 5.552276 | 5.887179 |
| C | -0.407597 | 7.537043 | 7.589921 |
| H | 0.003503 | 8.308898 | 8.234839 |
| C | -2.047405 | 6.857296 | 5.929380 |
| C | -1.512121 | 7.830471 | 6.769758 |
| H | -1.966590 | 8.818594 | 6.777674 |
| C | 4.159223 | -2.130566 | -1.562370 |
| C | 3.880386 | -3.128007 | 1.064197 |
| H | 3.743620 | -3.521857 | 2.064354 |
| C | 4.670211 | -3.781817 | 0.144052 |
| H | 5.185768 | -4.697956 | 0.413566 |
| C | 4.844447 | -3.266377 | -1.156908 |
| H | 5.549067 | -3.740026 | -1.829940 |
| C | 3.193492 | -1.939672 | 0.695186 |
| C | 3.259176 | -1.466984 | -0.658089 |
| C | 2.481102 | -1.146665 | 1.651496 |
| C | 2.501142 | -0.316543 | -1.027793 |
| H | 2.521065 | 0.032008 | -2.053167 |
| C | 1.752803 | -0.047585 | 1.263535 |
| H | 1.219130 | 0.525405 | 2.011944 |
| C | 1.746115 | 0.359252 | -0.092006 |
| H | 1.174221 | 1.234064 | -0.381125 |
| C | 3.778412 | -1.007588 | 3.687460 |
| C | 4.068551 | -1.662661 | 4.992112 |

| | | | |
|---|---|---|---|
| C | 3.660944 | -2.989969 | 5.240651 |
| C | 3.802246 | -3.568460 | 6.542582 |
| C | 4.437567 | -2.817860 | 7.535435 |
| H | 4.561763 | -3.253029 | 8.524248 |
| C | 4.909790 | -1.518043 | 7.262396 |
| H | 5.400650 | -0.955121 | 8.051418 |
| C | 4.726791 | -0.941353 | 6.011411 |
| H | 5.046543 | 0.076906 | 5.813179 |
| C | 5.065350 | -2.039819 | -3.870520 |
| C | 5.825085 | -1.083565 | -4.704512 |
| C | 6.165220 | -1.485129 | -6.019837 |
| H | 5.870463 | -2.476129 | -6.349161 |
| C | 6.824472 | -0.619389 | -6.874483 |
| H | 7.070498 | -0.930275 | -7.886123 |
| C | 7.168475 | 0.677297 | -6.441931 |
| H | 7.669791 | 1.371204 | -7.112860 |
| C | 6.866379 | 1.100789 | -5.149948 |
| C | 6.197318 | 0.206829 | -4.247872 |
| C | 0.996629 | 3.147524 | 2.633089 |
| H | 0.553110 | 2.558412 | 3.424325 |
| C | 2.380984 | 3.027138 | 2.391569 |
| H | 2.985602 | 2.360612 | 2.996611 |
| C | 0.201468 | 3.953999 | 1.849090 |
| H | -0.861908 | 4.002833 | 2.057979 |
| C | 2.953546 | 3.697472 | 1.331216 |
| C | 0.764949 | 4.696557 | 0.773376 |
| C | 2.167417 | 4.552849 | 0.488777 |
| C | -0.007237 | 5.608787 | -0.028498 |
| C | 2.741185 | 5.268468 | -0.596258 |
| H | 3.796586 | 5.168965 | -0.823748 |
| C | 0.594716 | 6.295573 | -1.073599 |
| H | 0.019473 | 7.008723 | -1.649236 |
| C | 1.964186 | 6.114703 | -1.355031 |
| H | 2.410572 | 6.671659 | -2.171554 |
| C | -2.366077 | 6.319586 | -0.423463 |
| C | -3.620097 | 6.671717 | 0.288003 |
| C | -4.618653 | 7.347532 | -0.457794 |
| H | -4.412932 | 7.590462 | -1.494444 |
| C | -5.829722 | 7.687266 | 0.119309 |
| H | -6.585653 | 8.206628 | -0.463404 |
| C | -6.089992 | 7.357956 | 1.462815 |
| H | -7.041437 | 7.612303 | 1.924429 |
| C | -5.131143 | 6.701889 | 2.231728 |
| C | -3.868201 | 6.353533 | 1.643707 |
| C | 5.392239 | 3.644961 | 1.846894 |
| C | 6.729755 | 3.469592 | 1.196974 |
| C | 6.886881 | 3.058175 | -0.147122 |
| C | 7.887977 | 3.753321 | 1.959269 |

| | | | |
|---|---|---|---|
| H | 7.765454 | 4.063135 | 2.991680 |
| C | 9.147979 | 3.656289 | 1.388577 |
| H | 10.029827 | 3.883485 | 1.981462 |
| C | 8.186255 | 2.980897 | -0.741860 |
| C | 9.301585 | 3.278766 | 0.039288 |
| H | 10.290548 | 3.210174 | -0.408090 |
| C | 2.875667 | -0.233847 | -5.953124 |
| H | 3.796167 | -0.193811 | -6.525063 |
| C | 2.032704 | -1.357232 | -6.083896 |
| H | 2.304870 | -2.160913 | -6.756624 |
| C | 2.553532 | 0.796952 | -5.096805 |
| H | 3.231927 | 1.632579 | -4.970620 |
| C | 0.848549 | -1.438989 | -5.370766 |
| C | 1.344619 | 0.745982 | -4.345813 |
| C | 0.457831 | -0.377203 | -4.485515 |
| C | 0.983729 | 1.789961 | -3.430682 |
| C | -0.752821 | -0.392426 | -3.733748 |
| H | -1.459597 | -1.206326 | -3.848056 |
| C | -0.160991 | 1.706241 | -2.673120 |
| H | -0.369967 | 2.471863 | -1.929536 |
| C | -1.038986 | 0.612590 | -2.836943 |
| H | -1.938830 | 0.563645 | -2.239606 |
| C | 1.841793 | 3.950341 | -4.160062 |
| C | 3.066561 | 4.770538 | -4.231879 |
| C | 4.306230 | 4.287245 | -3.751886 |
| C | 2.987318 | 6.086247 | -4.751021 |
| H | 2.031966 | 6.445892 | -5.120160 |
| C | 4.104912 | 6.904529 | -4.749397 |
| H | 4.038603 | 7.917541 | -5.137049 |
| C | 5.333554 | 6.441759 | -4.229856 |
| H | 6.203665 | 7.094399 | -4.208915 |
| C | 5.453715 | 5.147702 | -3.726833 |
| C | 0.346621 | -3.861355 | -5.522298 |
| C | -0.768401 | -4.831818 | -5.652395 |
| C | -0.450137 | -6.171835 | -5.984287 |
| H | 0.594615 | -6.452060 | -6.072235 |
| C | -2.124005 | -4.455462 | -5.522805 |
| C | -1.455819 | -7.092347 | -6.231073 |
| H | -1.205313 | -8.117544 | -6.490359 |
| C | -2.809379 | -6.703025 | -6.171510 |
| H | -3.601336 | -7.415660 | -6.390978 |
| C | -3.159632 | -5.396487 | -5.837286 |
| C | -4.547006 | -0.098884 | 0.615534 |
| H | -4.542218 | -0.658897 | 1.544608 |
| C | -4.229054 | -0.742972 | -0.599001 |
| H | -3.968174 | -1.793622 | -0.591628 |
| C | -4.860083 | 1.248660 | 0.632634 |
| C | -4.231525 | -0.044093 | -1.786284 |

| | | | |
|---|---|---|---|
| H | -3.991023 | -0.569990 | -2.703083 |
| C | -4.800376 | 2.029004 | -0.568037 |
| C | -4.492390 | 1.354997 | -1.800426 |
| C | -5.033187 | 3.431682 | -0.573996 |
| H | -5.261167 | 3.950902 | 0.350899 |
| C | -4.447952 | 2.130627 | -3.007465 |
| C | -4.979051 | 4.139442 | -1.755613 |
| H | -5.172187 | 5.205958 | -1.754087 |
| C | -4.699311 | 3.490583 | -2.977620 |
| H | -4.721027 | 4.048769 | -3.907932 |
| C | -6.164012 | 1.410336 | 2.716366 |
| C | -6.342171 | 2.181607 | 3.972590 |
| C | -5.469449 | 3.220286 | 4.369616 |
| C | -7.450151 | 1.859108 | 4.791708 |
| H | -8.108780 | 1.056323 | 4.478126 |
| C | -7.699102 | 2.557940 | 5.961901 |
| H | -8.556367 | 2.302975 | 6.578599 |
| C | -5.743154 | 3.950084 | 5.575314 |
| C | -6.847358 | 3.608281 | 6.355675 |
| H | -7.034463 | 4.165173 | 7.271345 |
| C | -3.432032 | 1.841378 | -5.264668 |
| C | -3.434713 | 0.918786 | -6.430322 |
| C | -3.033602 | 1.432516 | -7.692161 |
| H | -2.829991 | 2.496775 | -7.782968 |
| C | -3.834720 | -0.432747 | -6.331846 |
| C | -3.014061 | 0.616585 | -8.813794 |
| H | -2.720729 | 1.024360 | -9.777326 |
| C | -3.834149 | -1.267859 | -7.503366 |
| C | -3.402597 | -0.734159 | -8.717479 |
| H | -3.396598 | -1.374665 | -9.596243 |
| C | 0.503925 | -3.973106 | -1.195642 |
| H | 1.540396 | -3.787848 | -1.448048 |
| C | -0.526890 | -3.437849 | -1.991199 |
| H | -0.264936 | -2.877704 | -2.879800 |
| C | 0.208888 | -4.710660 | -0.064876 |
| C | -1.849611 | -3.621114 | -1.650215 |
| H | -2.616617 | -3.193016 | -2.285019 |
| C | -1.150539 | -4.849734 | 0.376211 |
| C | -2.195702 | -4.318792 | -0.458203 |
| C | -1.490202 | -5.481317 | 1.604197 |
| H | -0.716899 | -5.888177 | 2.246824 |
| C | -3.562097 | -4.523071 | -0.052543 |
| C | -2.807783 | -5.576666 | 1.994521 |
| H | -3.056613 | -6.045948 | 2.940273 |
| C | -3.849792 | -5.113613 | 1.165280 |
| H | -4.879855 | -5.255919 | 1.463863 |
| C | -5.851698 | -3.763113 | -0.716836 |
| C | -6.700113 | -3.527399 | -1.917538 |

| | | | |
|---|---|---|---|
| C | -6.163738 | -3.281894 | -3.199865 |
| C | -7.044003 | -3.114708 | -4.322570 |
| C | -8.422495 | -3.139470 | -4.117549 |
| H | -9.080082 | -2.998049 | -4.972063 |
| C | -8.953936 | -3.361197 | -2.832089 |
| H | -10.031181 | -3.399497 | -2.695203 |
| C | -8.109637 | -3.559109 | -1.749714 |
| H | -8.515456 | -3.808104 | -0.772649 |
| C | 2.275583 | -6.043806 | 0.133781 |
| C | 3.220793 | -6.660230 | 1.105076 |
| C | 3.081725 | -6.534602 | 2.507042 |
| C | 4.160205 | -6.950363 | 3.361793 |
| C | 5.271232 | -7.588964 | 2.810486 |
| H | 6.062199 | -7.936263 | 3.473195 |
| C | 5.362763 | -7.782945 | 1.416186 |
| H | 6.230651 | -8.288165 | 1.001707 |
| C | 4.364389 | -7.311577 | 0.581072 |
| H | 4.451960 | -7.414436 | -0.495124 |
| N | -0.095073 | 2.981766 | 5.902856 |
| H | -0.704741 | 3.359944 | 5.179456 |
| N | -1.286535 | -2.926543 | 4.640520 |
| H | -0.323980 | -3.251284 | 4.761190 |
| N | 4.429410 | -1.489325 | -2.785251 |
| H | 4.579597 | -0.480470 | -2.707570 |
| N | 2.652297 | -1.441727 | 3.038502 |
| H | 2.270271 | -2.321808 | 3.384901 |
| N | -4.223856 | 1.459839 | -4.225069 |
| H | -4.554646 | 0.494172 | -4.304346 |
| N | -5.224456 | 1.879510 | 1.844801 |
| H | -4.715352 | 2.713977 | 2.151043 |
| N | -0.013454 | -2.541009 | -5.517258 |
| H | -1.023161 | -2.389021 | -5.425748 |
| N | 1.871170 | 2.895580 | -3.296365 |
| H | 2.810767 | 2.680278 | -2.943832 |
| N | 4.316903 | 3.518100 | 1.005051 |
| H | 4.553123 | 3.388811 | 0.017924 |
| N | -4.575933 | -4.178348 | -0.968370 |
| H | -4.316435 | -4.109357 | -1.953803 |
| N | -1.343783 | 5.844082 | 0.347906 |
| H | -1.598703 | 5.706083 | 1.329503 |
| N | 1.235319 | -5.338299 | 0.670353 |
| H | 1.184744 | -5.366031 | 1.692345 |
| O | 8.243015 | 2.619555 | -2.033622 |
| O | 5.859729 | 2.698433 | -0.929955 |
| O | 4.485524 | 3.043532 | -3.312173 |
| O | 6.580311 | 4.645147 | -3.202788 |
| O | 7.155123 | 2.327694 | -4.695567 |
| O | 5.957710 | 0.671692 | -3.015521 |

| | | | |
|---|---|---|---|
| O | -2.025081 | 4.660270 | 5.056220 |
| O | -3.115037 | 7.078732 | 5.146424 |
| O | -2.981589 | 5.733883 | 2.435230 |
| O | -5.329712 | 6.370036 | 3.513813 |
| O | -4.869700 | 4.919492 | 5.908488 |
| O | -4.375257 | 3.576906 | 3.692791 |
| O | 2.019855 | -5.999207 | 3.105318 |
| O | 4.068048 | -6.636351 | 4.667926 |
| O | 3.168673 | -3.780642 | 4.280445 |
| O | 3.319436 | -4.808303 | 6.715982 |
| O | 1.476437 | -6.987696 | 5.920620 |
| O | 0.654602 | -4.596230 | 5.187694 |
| O | -4.253829 | -2.527958 | -7.357774 |
| O | -4.242576 | -0.993867 | -5.193828 |
| O | -2.510305 | -3.235514 | -5.145063 |
| O | -4.428874 | -4.956526 | -5.794141 |
| O | -4.854017 | -3.210136 | -3.439941 |
| O | -6.480789 | -2.934088 | -5.526152 |
| O | -2.756688 | 2.900046 | -5.264805 |
| O | 0.806142 | 4.206490 | -4.833126 |
| O | -2.256503 | 6.459770 | -1.663181 |
| O | 1.006450 | 3.623945 | 7.816277 |
| O | 5.269223 | 3.893108 | 3.058647 |
| O | 4.491978 | -0.090956 | 3.225640 |
| O | -3.449640 | -3.675820 | 4.830085 |
| O | 2.447365 | -6.150915 | -1.100214 |
| O | 1.540425 | -4.226690 | -5.459753 |
| O | -6.862808 | 0.406283 | 2.458898 |
| O | 4.974684 | -3.255813 | -4.138487 |
| O | -6.301077 | -3.599882 | 0.439671 |
| Ga | 6.395811 | 2.638300 | -2.863208 |
| Ga | -3.731632 | 5.400681 | 4.256511 |
| Ga | 2.424364 | -5.478458 | 5.013199 |
| Ga | -4.475557 | -2.978726 | -5.386465 |
| K | -0.184935 | 2.391535 | -6.700787 |
| K | 5.951587 | -4.563916 | 4.017920 |
| K | -5.710014 | 7.509713 | 5.922948 |
| K | -7.681219 | -1.179292 | 0.559498 |
| K | 3.030881 | -5.153012 | -3.505377 |
| K | -3.327431 | 2.830499 | 6.918142 |
| K | -1.618697 | 4.759244 | -3.593188 |
| K | 9.074107 | 4.157802 | -4.141220 |
| K | -6.152892 | -4.377734 | -7.839305 |
| K | 5.545565 | 0.423264 | 0.674284 |
| K | 3.823899 | -7.417848 | 7.262625 |
